# Molecular Profiles of HCV Cirrhotic Tissues Derived in a Panel of Markers with Clinical Utility for Hepatocellular Carcinoma Surveillance

**DOI:** 10.1371/journal.pone.0040275

**Published:** 2012-07-05

**Authors:** Ricardo C. Gehrau, Kellie J. Archer, Valeria R. Mas, Daniel G. Maluf

**Affiliations:** 1 University of Virginia, Department of Surgery, Transplant Division. Charlottesville, Virginia, United States of America; 2 Virginia Commonwealth University, Department of Biostatistics. Richmond, Virginia, United States of America; 3 Massey Cancer Center, Biostatistics Shared Resource. Richmond, Virginia, United States of America; The University of Hong Kong, Hong Kong

## Abstract

**Background:**

Early hepatocellular carcinoma (HCC) detection is difficult because low accuracy of surveillance tests. Genome-wide analyses were performed using HCV-cirrhosis with HCC to identify predictive signatures.

**Methodology/Principal Findings:**

Cirrhotic liver tissue was collected from 107 HCV-infected patients with diagnosis of HCC at pre-transplantation and confirmed in explanted livers. Study groups included: 1) microarray hybridization set (n = 80) including patients without (woHCC = 45) and with (wHCC = 24) HCC, and with incidental HCC (iHCC = 11); 2) independent validation set (n = 27; woHCC = 16, wHCC = 11). Pairwise comparisons were performed using moderated t-test. FDR<1% was considered significant. L_1_-penalized logistic regression model was fit for woHCC and wHCC microarrays, and tested against iHCC. Prediction model genes were validated in independent set by qPCR. The genomic profile was associated with genetic disorders and cancer focused on gene expression, cell cycle and cell death. Molecular profile analysis revealed cell cycle progression and arrest at G2/M, but progressing to mitosis; unregulated DNA damage check-points, and apoptosis. The prediction model included 17 molecules demonstrated 98.6% of accuracy and correctly classified 6 out of 11 undiagnosed iHCC cases. The best model performed even better in the additional independent set.

**Conclusions/Significances:**

The molecular analysis of HCV-cirrhotic tissue conducted to a prediction model with good performance and high potential for HCC surveillance.

## Introduction

Chronic infection with hepatitis C virus (HCV) is considered a major risk for chronic liver failure. Recent epidemiological studies and data from the World Health Organization (WHO; www.who.int) estimated the global prevalence around 160–170 million of people chronically infected with HCV [1]. In the United States (USA), HCV infection is considered the main blood-borne disease with an estimated prevalence range of 1.3–1.9% with 32,973 new reported cases only in 2009 [1]–[3]. But this tendency might have a negative slope for the following years. Recent estimations predicted a decreasing HCV-infection prevalence for several regions, including the USA, of about 22–24% for the 20 year decade [4], [5]. However, chronic liver infection with HCV associates with the development of hepatic malignancies. Hepatocellular carcinoma (HCC) is considered the main primary liver cancer and the third leading cause of death worldwide [6]. Chronic HCV infection is the second most common cause of all HCCs, and the leading etiology in Japan, Egypt, and within the USA [7]–[9]. However, the carcinogenic processes leading to HCC development in HCV-cirrhosis cases are not yet well understood. It has been postulated that direct and indirect interactions between HCV-encoded proteins and host hepatic cells may contribute to the malignant process [10]. In addition to this, the chronic inflammation scenario accompanied by immune-mediated destruction of infected hepatocytes, oxidative stress, virus-induced apoptosis, DNA damage leading to genomic heritable aberrations, and continuous hepatic regeneration processes may also be involved in promoting HCC development within the HCV-cirrhotic background [9], [10].

Liver transplantation (LT) demonstrated to have therapeutic advantages as surgical treatment option of patients with early HCC [11]. Moreover, end stage liver disease due to chronic HCV infection-based cirrhosis is the principal contributor to the LT procedure rates in European countries, Japan, and the USA [11], [12]. Furthermore, the priority allocation system of the United Network for Organ Sharing (UNOS) organization ascribes extra-points to those HCC affected patients who meet currently adopted inclusion criteria [13]–[15]. As a consequence of the poor clinical outcomes of patients with hepatitis C-induced cirrhosis and who are diagnosed with advanced hepatocellular carcinoma stages improved markers for early detection are needed. Nowadays, surveillance for HCC is recommended every 6 to 12 months in patients at high risk, but a strict consensus has not been reached yet [16], [17]. Conventional HCC surveillance tests are based on radiological imaging techniques (US, CT scan, and MRI) and/or serological biomarkers (e.g. alpha-fetoprotein). In spite of recent technological advances, imaging-based methods still unfortunately lack of sufficient accuracy and sensitivity for early diagnosis of small HCC lesions (<2 cm) mainly associated with the nodular cirrhotic liver background [16], [18], [19]. In a similar manner, alpha-fetoprotein demonstrated to be controversial as consequence of its low diagnostic performance as HCC screening biomarker, while other promising circulating molecules need further clinical evaluation [16], [20].

A better understanding of the cirrhotic liver tissue biology in chronically HCV-infected patients might reveal involved carcinogenic promoter mechanisms in HCC development, and thus improving the screening biomarker discovery. Genomic-based approaches using oligonucleotide microarrays represent reliable technology of choice. In this study, a transcriptome -based analysis was performed in HCV-cirrhotic non-tumor tissue from patients with and without HCC aiming to, 1) biologically characterize the molecular events leading to HCC development in the HCV-cirrhotic tissue, 2) identify a multigenic classifier capable of detecting the presence of hepatocellular carcinoma in cirrhotic tissues, and 3) validate the genes included into the identified multigenic classifier in an independent set of patients.

## Materials and Methods

### Patients and Tissue Samples Collection

This study has been approved by the Institutional Review Board at Virginia Commonwealth University, and written informed consent was obtained from all patients. The present study included 107 HCV-cirrhotic patients who underwent LT treatment for end-stage liver disease. All patients were screened for HCC lesions previous to LT surgery by radiological techniques (CT scan and MRI). Histopathological examination was performed by serially sectioning up to 5 mm for each explanted liver. Demographic and clinico-pathological characteristics were collected for all included patients (**Table 1**). Cirrhotic tissue (non-tumor) was obtained from each HCV-cirrhotic liver and snap-frozen in liquid nitrogen or collected in RNAlater™ reagent (Ambion Inc., Austin, TX, USA) following manufacturer instructions, and stored at −80°C until use.

**Table 1:** Demographic and clinicopathological characteristics of HCV-cirrhotic patient groups.

Study sets		Microarray hybridization set				Independent validation set		
HCV-cirrhosis groups		WoHCC(n = 45)	Whcc(n = 24)	iHCC(n = 11)	*p*-value	woHCC (n = 16)	wHCC*(n = 11)	*p*-value
Age (years)	Mean ± SD (range)	50.6±6.2(30–62)	54.6±7.2(43–68)	54.7±7.9(39–65)	0.0349	52.9±6.0 (40–62)	51.8±5.6 (44–64)	0.6296
Gender	Male	37	19	11	0.3148	15	11	0.3003
	Female	8	3	0		1	0	
Race	White	38	18	8	0.6637	11	8	0.8941
	Black/AfricanAmerican	6	2	1		4	2	
	Other	1	2	2		1	1	
Waiting list time in days	Mean ± SD (range)	272±345(2–1580)	355±627(15–2100)	328±474(4–1525)	0.7726	253±389 (61–1408)	218±335 (13–1107)	0.8271
MELD score at LT	Mean ± SD (range)	20.0±7.8(6–39)	23.4±4.4(12–29)	21.6±7.3(13–37)	0.1749	20.2±6.2 (12–36)	23.2±2.1 (22–28)	0.1977
Tumor stage (%)	T1		1 (4.2)	3 (27.3)	0.2297		2 (18.2)	NA
	T2		16 (66.7)	6 (54.5)			9 (81.8)	
	T3		5 (20.8)	1 (9.1)			0 (0.0)	
	T4		2 (8.3)	1 (9.1)			0 (0.0)	
HCC Lesions (%)	Single		9 (37.5)	5 (45.4)	0.6556		7 (63.4)	NA
	Multiple		15 (62.5)	6 (54.6)			4 (36.4)	
Size (cm)	Mean ± SD (n; range)		2.3±1.3(44; 0.4–5.2)	1.3±0.8(20; 0.1–4.0)	0.0040		2.2±1.4(16; 0.7–6.0)	NA

(*)Histopathological examination identified one incidental HCC viable unique lesion (size: 1.7 cm) in the explanted liver from one HCV-cirrhosis case of the independent patients’ set. *P*-values were calculated among HCV-cirrhotic groups for each study set.

### RNA Isolation and Microarray Hybridization

**Figure 1:**
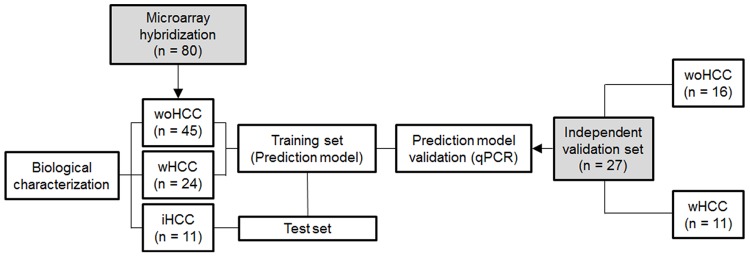
Schematic representation of the study design. HCV-cirrhotic tissue samples were divided in two main groups: 1) microarray hybridization (n = 80), and 2) independent validation set (n = 27), represented in gray squares. Pairwise comparisons among woHCC, wHCC, and iHCC groups of hybridized microarrays were performed for molecular pathway analyses. L_1_-penalized regression model was fit for woHCC and wHCC groups with hybridized microarrays (training set). The iHCC group was the test set for the identified prediction model. The independent set (n = 27) validated the expression of 9 randomly selected genes from the prediction model.

Total RNA was extracted from HCV-cirrhotic liver tissues using TRIzol reagent (Life technologies, Carlsbad, CA, USA) following the Affymetrix GeneChip® Expression Analysis Manual (Affymetrix, Santa Clara, CA, USA) guidelines and recommendations. All isolated RNA samples followed and met purity and integrity quality control criteria previously established in our laboratory [21]. Microarray hybridization was performed for 80 HCV-cirrhotic patients including cases without HCC (woHCC, n = 45), with HCC (wHCC, n = 24), and with incidental HCC (iHCC, n = 11) (**Figure 1** and **Table 1**). Reactions for cDNA synthesis and in vitro transcription for labeled cRNA probe, Affymetrix™ HG-U133A v2.0 GeneChip® microarray hybridization, image generation, and probesets reading process were performed as reported previously [22].

### Microarray Quality Control and Data Analysis

Eighty Affymetrix HG-U133Av2 GeneChips were available from 80 HCV-cirrhotic patients undergoing LT, including 45 woHCC subjects, 11 iHCC subjects, and 24 wHCC subjects. Quality of the hybridized arrays was assessed by examining the average background, scaling factor, percent of probe sets called present by the detection call algorithm, and the 3′:5′ ratio for *GAPDH* and *ACTIN*. Additionally, probe level linear models using affyPLM were fit and plots of the residuals were examined for each hybridized GeneChip. Thereafter, control probe sets and probe sets considered absent in all samples were removed [23], leaving 18,017 probe sets for statistical analysis. For each probe set, a moderated t-test was used to compare cirrhosis woHCC *versus* cirrhosis wHCC or iHCC; cirrhosis woHCC *versus* cirrhosis wHCC; as well as to compare cirrhosis woHCC *versus* cirrhosis with iHCC using the limma Bioconductor package [24], [25] in the R programming environment [26]. To adjust for the multiple hypothesis tests, the *p*-values were used in estimating the false discovery rate (FDR) using the Benjamini and Hochberg method [27]. Probe sets having an FDR<0.01 were considered significant.

### Predictive Class Classifier Analysis

To predict group membership using the gene expression data, an L_1_-penalized logistic regression model was fit using the *glmpath* package in the R programming environment [28] using the subjects with cirrhosis woHCC and those wHCC as the training set. The Akaike Information Criterion (AIC) was used to select the best fitting model. To evaluate model performance, the subjects with iHCC were used as an independent test set (**Figure 1**). Supervised hierarchical clustering analysis using Ward's method was applied to the identified gene expression matrix including all samples from woHCC, wHCC, and iHCC groups. A heatmap was also represented in accordance and together with the obtained sample clusters.

### Gene Ontology and Gene Pathway Analysis

Ingenuity Pathway Analysis (IPA) tool version 9.0 (http://www.ingenuity.com) was applied for gene networks interaction and pathway function analysis. Statistically differentially expressed genes identified from the wHCC *vs.* woHCC, iHCC *vs.* woHCC, and aHCC (wHCC + iHCC) *vs.* woHCC pairwise comparisons were incorporated in a list containing probesets ID, Gene symbol, Entrez gene ID as clone identifier, probeset intensity values for each study population, *p*-value, *q*-value, and fold change values. Identified genes were mapped in the Ingenuity Knowledge Base (genes + endogenous chemicals) as the reference set in IPA.

### Real Time PCR Assays

The expression of genes included into the best fitting prediction model were validated in an independent HCV-cirrhotic tissue sample group (independent validation set, n = 27; **Figure 1**) by real time PCR (qPCR) using Taqman® assays (Applied-Biosystems, Inc.). Clinicopathological characteristics of the independent validation set are described in **Table 1**. The gene list and Taqman® assays used for validation included: *RBPMS* (Hs00199302_m1), *ACSL6* (Hs00362960_m1), *DPP4* (Hs00175210_m1), *CENPN* (Hs00218401_m1), *C2orf43* (Hs00222492_m1), *KNTC1* (Hs00206854_m1), *MLL* (Hs00610538_m1), *SCMH1* (Hs00984275_m1), and *TNS1* (Hs00917032_m1). The qPCR amplification signal for each gene was normalized to the expression of *GAPDH* encoding gene (pre-developed Taqman® assay) as internal standard. Gene expression analysis was performed using delta-Ct value model (average Ct _gene_ – average Ct *_GAPDH_*). Statistical significances between wHCC *vs.* woHCC groups were evaluated by analysis of variance (ANOVA) test. A *p*-value <0.05 was considered statistically significant.

## Results

### Demographic and Clinicopathological Characteristics of Patients Included in the Study

A total of 107 HCV-cirrhotic liver tissue samples from individual patients were included in the study. The study design is described in **Figure 1**.

As it was previously mentioned, the demographic and clinicopathological characteristics of all HCV-cirrhotic patients included in the study and separated by groups are summarized in **Table 1**. All patients in this study cohort were infected with HCV genotype 1. From the group of patients used for microarray hybridization, 24/80 (30.0%) of cases were HCC lesions detected by radiology pre-LT. No HCC lesions were detected in 56 patients (70.0%) at pre-LT stage, but the pathological examination identified incidental HCC lesions in 11 (19.6%) of these cases. The remaining 45 HCV-cirrhosis cases were confirmed as without HCC. Demographically, the HCC patient population was significantly older than those patients without HCC. No statistical differences were found regarding gender and race, time on waiting list, MELD score at LT, and HCC TNM stage at LT. Moreover, most of incidental HCC lesions were small (ranged between 0.1–2.2 cm in size); with only one T4 HCC diffuse lesion was identified in a patient with macroregenerative cirrhosis. Ablation treatment was performed in all HCC patients at pre-LT (combined RFA, TACE).

The independent validation set of patients included 26 cases diagnosed without (n = 16) and with (n = 10) HCC at pre-LT using the conventional diagnosis methods. An incidental HCC lesion (1.7 cm in size) was identified in one patient in the analysis of the explanted liver. However, for analysis purposes, the patient was classified within HCV-cirrhosis with HCC. No significant differences were identified for age, gender, race, waiting time for LT, and MELD score at LT. All patients with HCC were staged as T1/T2 (**Table 1**).

### Differential Gene Expression Analysis and Molecular Pathways Identification

After oligonucleotide microarrays hybridization, control and absent calls were removed and a total of 18,017 probesets (Pset) were available for statistical analysis. Pairwise comparison analyses were performed between: 1) wHCC (n = 24) *vs.* woHCC (n = 45), 2) iHCC (n = 11) *vs.* woHCC (n = 45), and 3) aHCC (n = 35) *vs.* woHCC (n = 45) to identify differentially expressed Psets. From the analysis, the total of significantly and differentially expressed Psets was 1,252 (1,053 genes), 443 (392 genes), and 2,357 (1,945 genes), respectively. The analysis of biological functions was conducted by IPA tool in each of the three performed pairwise comparisons. In all three comparisons the pathway analysis revealed a similar deregulated genomic component, which was strongly associated with genetic disorders, and cancer as principal pathology. Moreover, the most significant molecular functions included gene expression, cell cycle and cell death regulation. In consideration to the similarities of molecular pathways among analyses, the complete molecular biological analysis was performed in the aHCC *vs.* woHCC pairwise comparison due to higher HCV-cirrhosis cases and number of identified genes.

**Figure 2:**
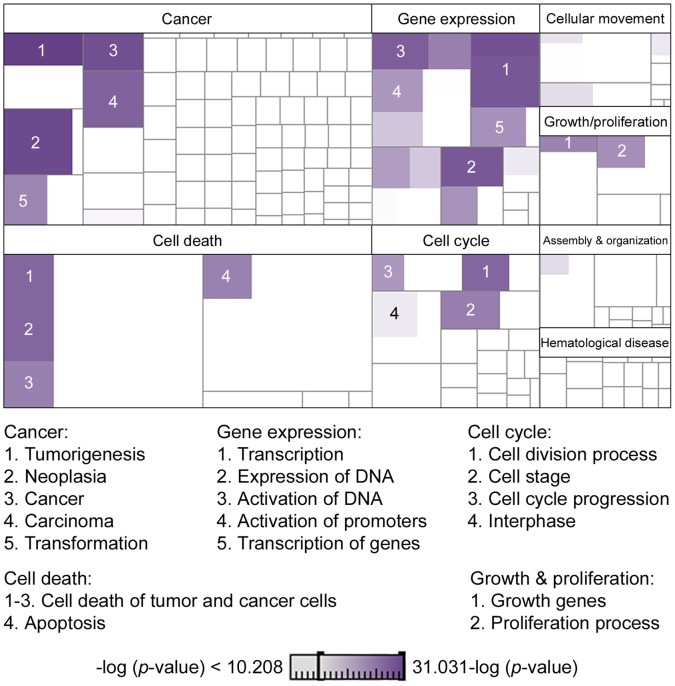
Molecular and cellular functions deregulated in HCV-cirrhosis with HCC. A *p*-value expressed as –log range of 10.2 to 30.0 was applied to identify the most significant cellular functions. Present functions and sub-groups are indicated in purple. Color intensity indicates the statistical significance for differential expressed function as the dark color the most significant. Involved cellular functions were identified using IPA tool.

From the analysis, cancer-related processes (tumorigenesis, carcinoma, neoplasia, and transformation), cell cycle (progression, division, interphase, and stage), cell death (apoptosis), cellular growth and proliferation, and gene expression regulation were the mostly significant (*p*<1.0E-10) associated molecular and cellular functions (**Figure 2**). Gene ontology and molecular pathways identified associated network functions related to cellular assembly and organization; DNA replication, recombination, and repair; genetic disorders, hematological disease, and cancer; and cell cycle control and cell morphology. Top five canonical pathways from a total of 120 identified as significantly (*p*≤0.01) altered between cirrhosis aHCC *vs.* woHCC included PI3K/AKT signaling (*p*  = 3.46E-09), molecular mechanisms of cancer (*p*  = 1.16E-08), PTEN signaling (*p* = 1.04E-07), apoptosis signaling (*p* = 1.64E-07), and chronic myeloid leukemia signaling (*p* = 1.95E-06). The canonical pathways complete list is detailed in **Table S1**.

From the analysis of hepatic disease associated function networks to provide key processes involved in organ detriment, the most significantly identified hepatotoxic alterations included liver necrosis/cell death (*p* = 3.80E-04 - 6.38E-01), HCC (*p* = 1.95E-03 - 4.89E-02), cirrhosis (*p* = 5.59E-03 - 6.66E-02), proliferation (*p* = 1.39E-02 - 5.99E-01), and regeneration of the liver (*p* = 1.69E-02 - 2.62E-01). Genes involved in cirrhosis development (*ABCG2*, *CTNNB1*, *B2M*, *IFNAR1*, *IFNAR2*), and hepatic cholestasis (*CYP7B1*) were found significantly down-regulated in patients without HCC.

### Molecular Mechanisms Associated with HCC Development

To better understand HCC associated molecular events, a detailed gene by gene analysis was performed including those differentially expressed molecules related to cell cycle and apoptosis control. Overall, critical genes encoding for positive cell cycle progression factors (*CCND1*, *CDK1*, *CDK2*, *E2F3*, *EIF4E*, and *MDM2*) were found significantly up-regulated in HCV-cirrhosis with HCC. Conversely, HCV-cirrhotic samples with absence of HCC till conserved high expression levels of genes exerting negative cell cycle regulation (*JUNB*, *CDKN2C*, *LIF*, *FOXO4*, *HMGA1*, *KLF4*, *NFKBIA*, *MYB*, *FZR1*, and *CDK11A/CDK11B*) throughout all cell cycle phases.

**Figure 3:**
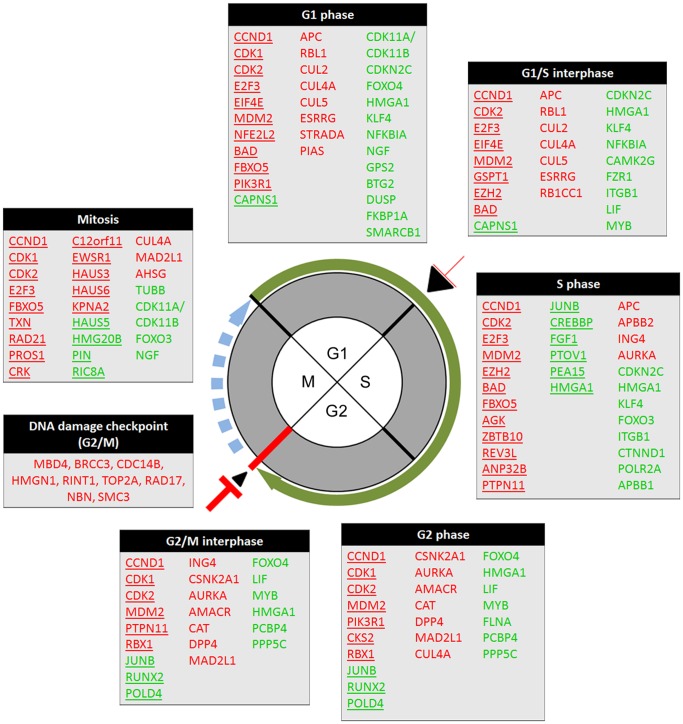
Cell cycle associated genes scheme. The sketch represents the cell cycle phases and interphases. Phases are named inside the main circle. Black bars indicate interphases. The G2/M interphase is indicated by a red bar. Cell cycle progression is illustrated by a round green arrow and potential progression by dashed blue arrow. Cell cycle blockade and progression are indicated by a red T and a black arrow head, respectively, whereas the size and thickness is proportional to the cell cycle regulation. Genes involved in each phase are listed in tables depending on the cell cycle phase. Red: up-regulation, green: down-regulation; genes involved in positive cell cycle progression are underlined.

Then, the genomic profile was analyzed for each cell cycle phase in concordance with each gene function (**Figure 3**). The complete list including all cell cycle-related genes classified by phase with expression, significant, biological role, and cell cycle impact is detailed in **Table S2**. From the analysis, most of genes associated with positive G1 phase and G1/S transition progression were up-regulated while arrest G1 phase-related genes were down-regulated in HCV-cirrhotic tissue with HCC. The expression of G1/S transition involved genes was equally up-and down-regulated. However, the majority of S phase intro-related genes were up-regulated as counterpart of S phase blockade down-regulated molecules in association with HCC development. These observations suggest cell cycle progression throughout initial (G1) and DNA synthesis (S) phases, but with a potential functional G1/S cell cycle checkpoint.

However, genes involved in negative cell cycle regulation at G2 and G2/M were found up-regulated together with G2/M checkpoint DNA damage-inducible genes (**Figure 3**). DNA double-strand break repair by homologous recombination associated genes (*NBS1*, *RAD50*, *RAD 52*, and *RAD54*) were also found to be significantly up-regulated (**Figure S1**). Spindle formation and chromosomal integrity associated genes were significantly up-regulated in favor of mitosis progression and cell proliferation in HCV-cirrhosis associated with HCC independently of the G2/M check-point cell cycle arrest trend.

**Figure 4:**
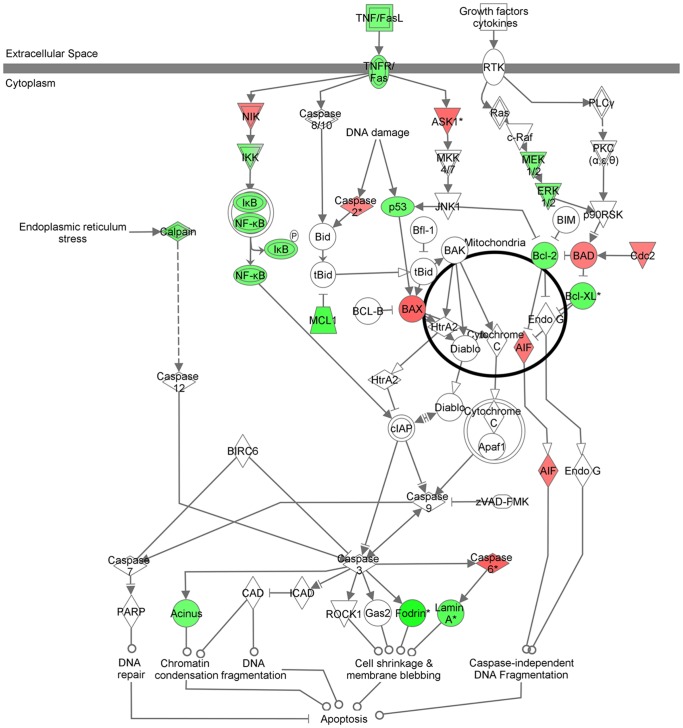
Apoptosis canonical pathway. The differentially expressed molecules are represented by color as up-regulated (red) and down-regulated (green). Color intensity indicates fold change values estimation for each molecule.

Apoptosis canonical pathway analysis revealed a pro-apoptotic trend associated with DNA damage and homeostasis, and may be triggered by caspase-independent DNA fragmentation (**Figure 4**). Pro-apoptotic (*BAX*, *BAD*, *CDC2*, and *AIF*) and anti-apoptotic (*BCL-2*, *BCL-XL*, and *MCL1*) genes mostly related to DNA damage were found to be up and down-regulated, respectively. Interestingly, genes associated with early cell cycle control (G1/S) and apoptosis in liver cells (*TGFβ1*, *EGFR*) were found downregulated in HCV-cirrhosis with HCC. The *TGFβ*1-encoding gene was identified as central down-regulated molecule of the top scored (score: 43) associated network function (**Figure S2**). In addition, *NOTCH1* and *GADD45B* encoding genes were found downregulated when cirrhotic patients develop HCC in concordance with previous reports [29], [30].

HCV-cirrhosis associated with HCC denotes increased cell proliferation over a pro-apoptotic and functional cell cycle checkpoints, but maybe associated not resolved DNA damage and accumulation of chromosomal aberrations leading to HCC development.

### Class Prediction Model for HCV-cirrhosis with HCC as Classification Variable

The study of differentially expressed genes in HCV-cirrhotic tissues may set the basis for the establishment of HCC predictive genomic signatures. To determine the best multigenetic prediction classifier for HCV-cirrhosis with associated HCC an L_1_-penalized logistic regression model was fit including wHCC and woHCC groups (n = 69) as training set (**Figure 1**). The best fitting regression prediction model included 17 significantly and differentially expressed Psets representing unique genes (**Table 2**). Briefly, those genes were found to be associated with cell death, growth and proliferation, tumor morphology, cellular development, cell movement, gene expression, and DNA replication, recombination, and repair. The calculated diagnostic performance of the prediction model displayed an accuracy of 98.6%, with an estimated sensitivity, specificity, positive and negative predictive values of 100%, 95.8%, 97.8%, and 100%, respectively, as illustrated by the receiver operating characteristic curve (**Figure S3**). Following, the prediction model was evaluated against iHCC cases (**Figure 1**). Among the undiagnosed iHCC cases, 54.5% (6/11) were correctly classified as HCV-cirrhotic patients with HCC. These results are of high clinical relevance because iHCC cases are critical since the HCC diagnosis failed by conventional tests at pre-LT stage.

**Table 2:** Best fitting prediction model for HCC detection in HCV-cirrhotic patients.

Probeset ID	Gene symbol	Entrez ID	Gene name
200601_at	ACTN4	81	Actinin, alpha 4
201291_s_at	TOP2A	7153	Topoisomerase (DNA) II alpha 170kDa
201500_s_at	PPP1R11	6992	Protein phosphatase 1, regulatory (inhibitor) subunit 11
201605_x_at	CNN2	1265	Calponin 2
205114_s_at	CCL3	414062	Chemokine (C-C motif) ligand 3
206316_s_at	KNTC1	9735	Kinetochore associated 1
209488_s_at	RBPMS	11030	RNA binding protein with multiple splicing
211207_s_at	ACSL6	23305	Acyl-CoA synthetase long-chain family member 6
211478_s_at	DPP4	1803	Dipeptidyl-peptidase 4
212080_at	MLL	4297	Myeloid/lymphoid or mixed-lineage leukemia (trithorax homolog, Drosophila)
219202_at	RHBDF2	79651	Rhomboid 5 homolog 2 (Drosophila)
220726_at	IGHD6–19	28486	Immuno-globulin heavy diversity 6–19
221216_s_at	SCMH1	22955	Sex comb on midleg homolog 1 (Drosophila)
221748_s_at	TNS1	7145	Tensin 1
222118_at	CENPN	55839	Centromere protein N
222192_s_at	C2orf43	60526	Chromosome 2 open reading frame 43
34406_at	PACS2	23241	Phosphofurin acidic cluster sorting protein 2

**Figure 5:**
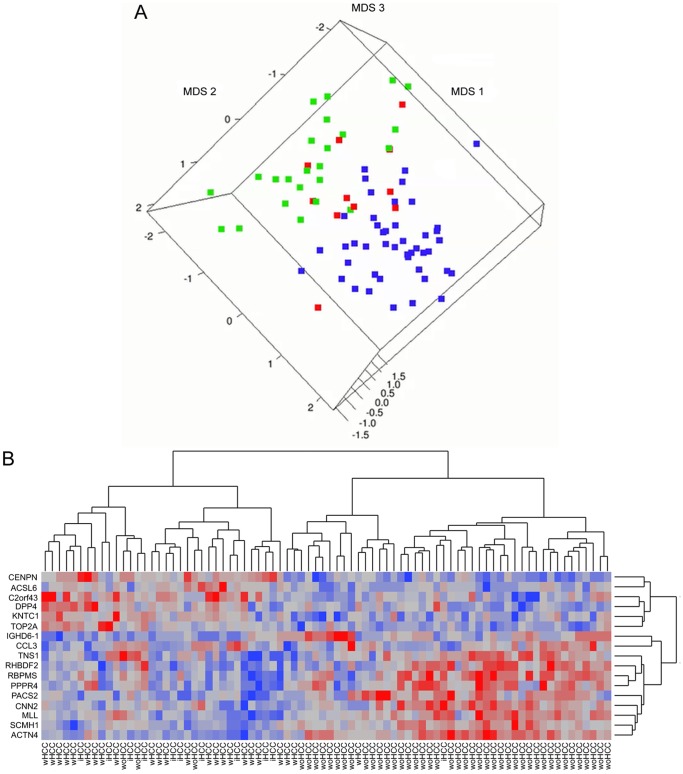
Best fitting prediction model for HCC identification in HCV-cirrhotic patients. L1-penalized regression model identified 17 differentially expressed Psets as listed in **Table 2**. **A)** Three-dimensional plot derived from applying classical multidimensional scaling (MDS) to the gene expression dataset for those genes identified by the L1-penalized regression model. Individual samples are represented by a colored square for wHCC (green), woHCC (blue), and iHCC (red). **B)** Two-way supervised hierarchical clustering and heatmap using ward’s method including all training set samples and Psets identified by the best fitting model.

The dimensionality of the gene expression dataset for those genes identified by the prediction model was reduced using classical multidimensional scaling. The resulting three-dimensional plot showed unique group of wHCC cases including 9 of 11 (81.8%) iHCC cases. Similarly, 39 of 45 (86.7%) of woHCC were separated from the other cases, and 2 of 11 (18.2%) iHCC cases were grouped together with woHCC cases (**Figure 5A**). A 2-D side-scatter graphic is further illustrated for additional details in **Figure S4**. Following, a supervised hierarchical clustering was performed including all Psets from the predictive model (**Figure 5B**). The resulting dendrogram associated the 80 HCV-cirrhotic cases in two well-defined groups in accordance with HCC status.

### Validation of the Prediction Model Genes using QPCR in and Independent Set of Patients

The identified genes from the prediction model were validated in an additional independent sample set of patients using QPCR (**Figure 1**). From the analysis, twelve randomly selected genes from the best LASSO-model were evaluated and nine were also found to be significantly differentially expressed in the independent sample set (**Table 3**). These results indicate a correlating expression trend with respect to microarray assays. Four validated genes (*RBPMS*, *ACSL6*, *DDP4*, and *CENPN*) were also previously identified in a similarly designed pilot study to test the L_1_-penalized logistic regression model algorithm [31].

**Table 3:** Independent validation study of the genes included in the prediction model.

Gene ID	Entrez ID	Gene name	Fold change (wHCC *vs.* woHCC)	*p*-value
ACSL6	23305	Acyl-CoA synthetase long-chain family member 6	2.1	0.004
CENPN	55839	Centromere protein N	1.7	0.005
RBPMS	11030	RNA binding protein with multiple splicing	−1.6	0.032
DPP4	1803	Dipeptidyl-peptidase 4	2.1	0.004
C2orf43	60526	Chromosome 2 open reading frame 43	1.7	0.010
KNTC1	9735	Kinetochore associated 1	1.6	0.047
MLL	4297	Myeloid/lymphoid or mixed-lineage leukemia (trithorax homolog, Drosophila)	−1.7	0.023
SCMH1	22955	Sex comb on midleg homolog 1 (Drosophila)	1.6	0.046
TNS1	7145	Tensin 1	−1.7	0.050

Core analysis was performed to interpret the data set in the context of biological processes, pathways and molecular networks. Molecular pathway analyses (http://www.ingenuity.com) directly associated the nine validated genes with cancer development and cell death. The most significant molecular functions included cell cycle mainly associated with arrest at G2/M transition (*DDP4*), progression (*KNTC1*, *MLL*, *SCMH1*), and mitosis (*CENPN*); and cell-to-cell signaling and interaction (*TNS1*), RNA processing (*RBPMS*), and cell proliferation (*ACSL6*).

**Figure 6:**
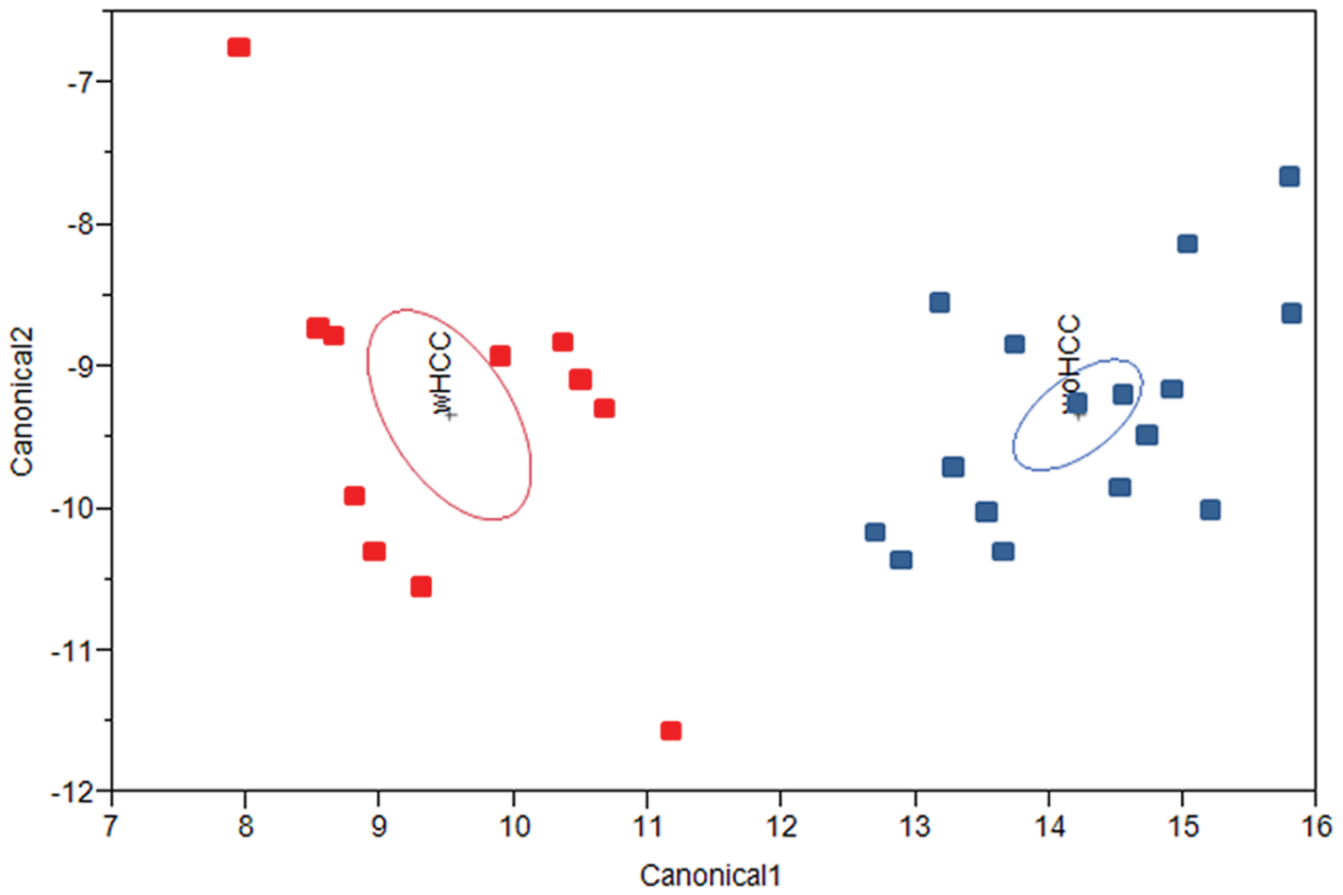
Identification of HCV-cirrhosis with and without HCC by qPCR validated genes. Canonical plot derived from applying quadratic discriminate analysis to nine validated genes from the best L1-penalized regression model in an independent set of HCV-cirrhotic samples. The 95% confidence ellipses of HCV-cirrhosis with (wHCC) and without HCC (woHCC) are illustrated in red and blue ovals, respectively. Individual samples are indicated by red (wHCC) and blue (woHCC) squares.

To visualize how the nine set of validated genes differentiate between HCV-cirrhosis regarding HCC diagnosis, a quadratic discriminate analysis was performed including all samples from the independent set (**Figure 6**). The resulting canonical plot showed two well-defined sets of HCV-cirrhotic samples separated in accordance with HCC diagnosis without miss-classification.

## Discussion

Hepatocellular carcinoma is frequently associated with advanced cirrhosis due to chronic hepatitis C. Progressive liver damage until HCC development in chronic HCV-infected patients encompasses deregulation of molecular pathways triggering malignant transformation. In this sense, the HCV-cirrhotic background has been further considered as a premalignant state [32]. Despite of animal and *in vitro* model establishment difficulties to study HCV infection outcomes, virus protein - host direct and indirect interactions were found thoroughly associated with liver cancer development. In HCV-cirrhotic patients, HCC is the final result of several rounds of hepatocytes destruction and regeneration by virus-induced immune-mediated mechanism and tissue damage associated processes such as oxidative stress, which impact on chromosomal DNA stability [9], [10], [33].

The insight into deregulated molecular mechanisms in HCV-cirrhotic tissue background embodies the understanding of malignant transformation. The present study analyzed the molecular profile associated with HCC development in human liver cirrhotic tissue from patients chronically infected with HCV, and who underwent LT. The microarray-based profiling analysis was found to be clearly associated with a carcinogenic component signed by an altered cell cycle regulation, cell death by apoptosis, cellular growth and proliferation. Similarly pre-cancerous genomic profiles were detected in previous studies focused on gene expression analysis of dysplastic nodules from advanced HCV-cirrhosis, and even in patients infected with HCV genotype 3 [34]–[36].

The study initially aimed to analyze the molecular mechanisms involved in hepatic malignancy development. Regulation of cell cycle progression appears as the principal altered cellular function in HCV-cirrhosis. The recent report from Kannan *et al*., [37] evaluated the HCV impact on cell cycle regulation and postulated a positive cell cycle progression until arrest at G2/M interphase with blockage to mitosis entrance in a HCV-infected hepatoma cell line model. In accordance with these results, the identified expression profile of cell cycle-related genes suggested a similar G2/M blockage trend in HCV-cirrhosis associated to HCC. In addition, the present results also suggested functional cell cycle checking mechanism at G1/S interphase in spite of unrestricted progression until G2/M.

It is well-understood that HCV induces strong cellular oxidative stress, which derive in DNA damage events and provoke accumulation of genomic mutations [38], [39]. Pro-mutagenic DNA lesions due to oxidative stress were reported to be more frequently in chronic HCV-infected patients [40]. From the study results, DNA damage check-point associated genes at G2/M were found significantly up-regulated indicating increased rates of DNA abnormalities in HCV-cirrhotic patients with HCC. Impaired DNA repair mechanisms due to demonstrated interactions with HCV non-structural proteins and prevalence of reactive oxygen and nitrogen species have been proposed as cause of HCC development in chronic HCV infection [38], [39], [41]. For instance, up-regulation of DNA repair genes in HCV-cirrhosis associated with HCC may be in response to a compensatory effect to oxidative stress and virus protein-induced inhibition on the DNA repair pathway.

Hepatocyte apoptosis represents a hallmark of chronic HCV infection in cirrhotic patients. It has been further reflected in the characterized genomic profile of HCV-cirrhosis with HCC. HCV core protein demonstrated to be by itself a pro-apoptotic factor through its interaction with the human myeloid cell factor 1 (Mcl-1) protein mimicking NOXA, the BH3-only member of the Bcl-2 family [42]. From the present study, key anti-apoptotic genes were found down-regulated maybe in response to unrepaired DNA adducts and mitochondrial membrane instability due to permanent oxidative stress induced by HCV. However, the HCV core protein itself was also implicated as tumor initiator inducing proliferation, anti-apoptosis and oxidative stress-related DNA adducts by activation of c-jun and stat-3 [43]. Indeed, the molecular profile proposes mitosis progression and cell proliferation, which has been demonstrated by other report to occur under the mitotic checkpoint inhibition in HCV infected hepatocyte [44]. All of this may allow cell proliferation carrying chromosomal aberrations and promoting a perfect pre-stage scenario for HCC development.

Accurate diagnosis and staging of HCC lesions in HCV-cirrhotic patients is of high importance for treatment and decision taking for surgical procedure. Moreover, appropriate HCC lesion diagnosis and staging allows access of HCV-patients to liver transplantation [12]. As consequence, the identification of suitable markers for early HCC diagnosis may reduce time to transplantation and thereby yield improved patient outcomes. The present study proposed a multigenic prediction model consisting of 17 differentially expressed genes able to accurately predict HCC lesions from the HCV-cirrhotic background with acceptable estimated analytical performance. Current HCC diagnosis and surveillance techniques are based on imaging radiological techniques and serological assays. However, the accurate HCC detection is often in an inverted relationship with the lack in efficiency of radiological imaging-based techniques, which directly correlates with the identification of incidental HCC lesions in explanted livers post-LT. Small tumor lesion size alone or combined with macronodular cirrhosis represent the most common obstacles for conventional imaging techniques [16], [18], [19]. In addition, infrequent HCC lesion variants such as diffuse cirrhosis-like HCC consisting of very small and numerous tumor nodules homogeneously distributed also attempt against to the proper radiological detection since it mimics a cirrhotic background [45]. Hereby, the proposed multigenic classifier was able to diagnosis iHCC lesions in more than 50% of the undiagnosed cases from a group of HCV-cirrhotic patients with iHCC. As consequence, the promising diagnostic power of the predictive gene signature might become of high importance for routine liver biopsies to rule out HCC in HCV-cirrhotic patients and contribute to the diagnostic arsenal.

Despite invasiveness of liver biopsy and the diagnostic limitation, the identified genomic signatures may appear as valuable biomarkers and may provide an extra tool in HCC diagnosis for the clinician.

In summary, the present study provided the genomic profile associated with HCC development in human HCV-infected cirrhotic livers, also corroborating in part previous *in vitro* studies. More importantly, a multigenic predictive classifier with high accuracy is proposed to identify HCC lesions only testing cirrhotic background. Further analyses in prospective patients’ cohorts are required to properly establish the clinical impact of the proposed genomic signature on HCC surveillance. Even more, the associated evaluation with currently recommended methods is crucial to definitely increase the HCC detection diagnostic performance and reduce to null the incidental HCC lesion rates.

## Supporting Information

Figure S1 **DNA double-strand break repair by homologous recombination pathway.** Up-regulated molecules in HCV-cirrhosis samples associated with HCC are colored in red.
(TIF)

Figure S2 **Top associated network functions.** The present associated network function was identified as top with a score of 43. The differentially expressed molecules are represented by color as up-regulated (red) and down-regulated (green). Color intensity indicates fold change values estimation for each molecule. (*) Molecules identified by at least two different Psets.
(TIF)

Figure S3 **Best fitting model Receiver Operating Characteristic (ROC) curve.** The ROC curve was established using samples from wHCC and woHCC groups. The iHCC samples were not included. Percentages for sensitivity (Sens), specificity (Spec), positive predictive value (PV+), and negative predictive value (PV-) are indicated at the top left.
(EPS)

Figure S4 **2D-side scatter plot for best fitting model.** All samples groups from training set were included. A red line divide the graphic to better identified the samples distribution depending on pathological characteristics.
(TIF)

Table S1 **Complete list of canonical pathways differentially expressed between HCV-cirrhotic liver tissues from patients with and without hepatocellular carcinoma.**
(DOCX)

Table S2 **Cell cycle deregulated genes HCV-cirrhotic tissue from patients with hepatocellular carcinoma.**
(DOCX)
