# Supplementary figures and images for: Molecular Profiles of HCV Cirrhotic Tissues Derived in a Panel of Markers with Clinical Utility for Hepatocellular Carcinoma Surveillance
Source: PLoS One. 2012 Jul 5;7(7):e40275. doi: 10.1371/journal.pone.0040275 (PMC3390353; doi:10.1371/journal.pone.0040275)

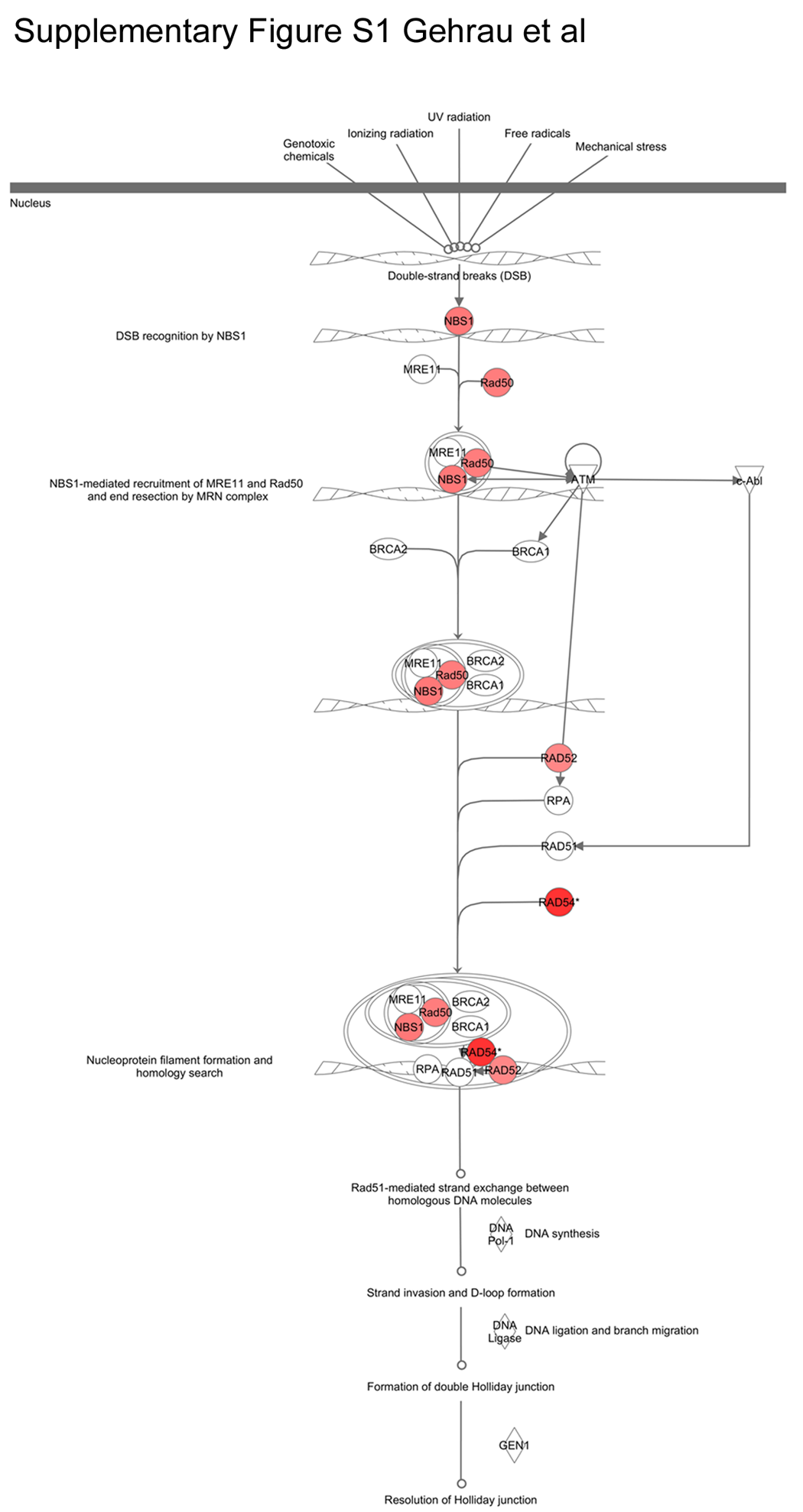

Supplement: Figure S1 — DNA double-strand break repair by homologous recombination pathway. Up-regulated molecules in HCV-cirrhosis samples associated with HCC are colored in red. (TIF) [file pone.0040275.s001.tif]

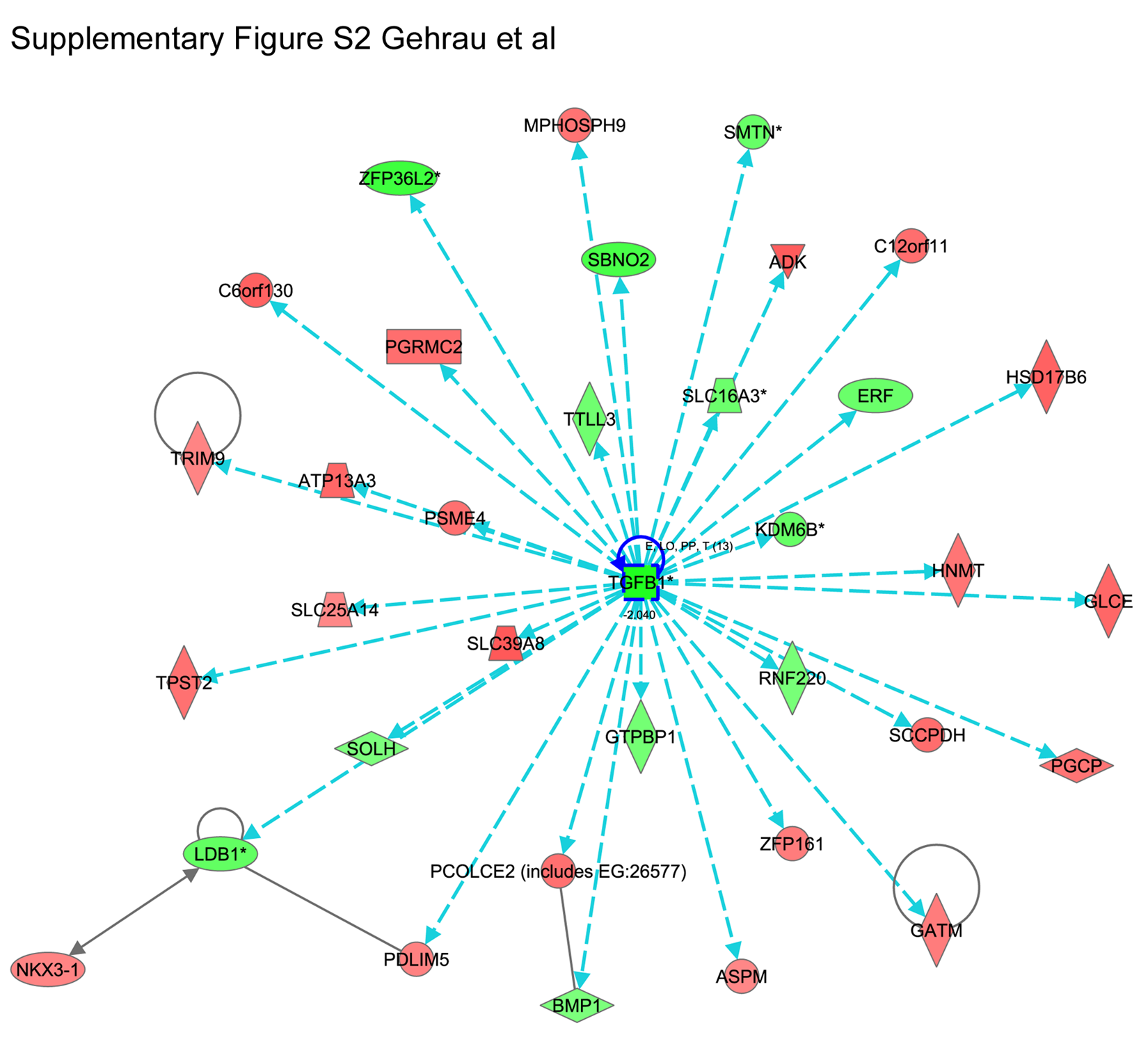

Supplement: Figure S2 — Top associated network functions. The present associated network function was identified as top with a score of 43. The differentially expressed molecules are represented by color as up-regulated (red) and down-regulated (green). Color intensity indicates fold change values estimation for each molecule. (*) Molecules identified by at least two different Psets. (TIF) [file pone.0040275.s002.tif]

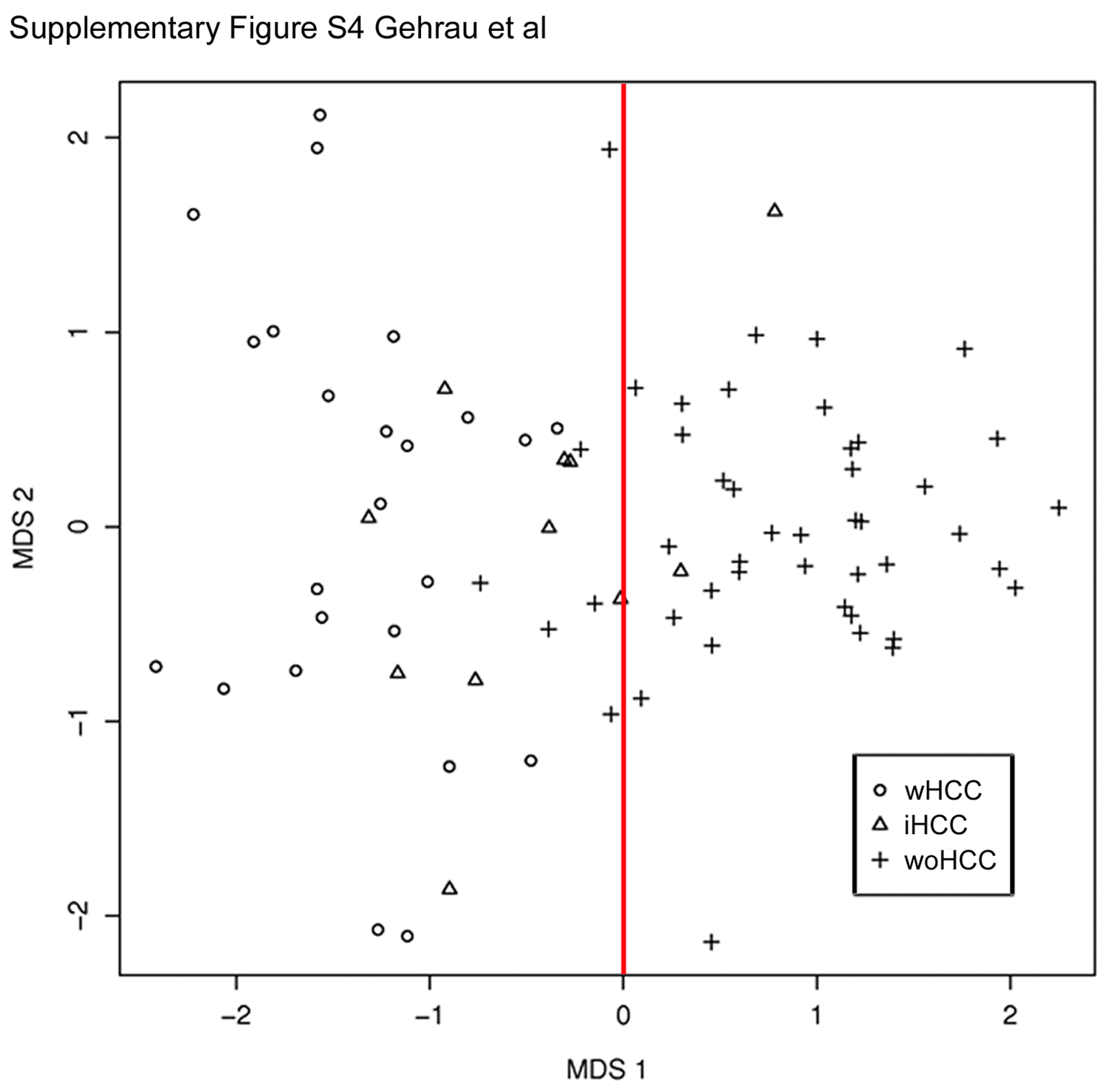

Supplement: Figure S4 — 2D-side scatter plot for best fitting model. All samples groups from training set were included. A red line divide the graphic to better identified the samples distribution depending on pathological characteristics. (TIF) [file pone.0040275.s004.tif]
