# Supplementary material for: Molecular Profiles of HCV Cirrhotic Tissues Derived in a Panel of Markers with Clinical Utility for Hepatocellular Carcinoma Surveillance
Source: PLoS One. 2012 Jul 5;7(7):e40275. doi: 10.1371/journal.pone.0040275 (PMC3390353; doi:10.1371/journal.pone.0040275)
Supplement: Table S1 — Complete list of canonical pathways differentially expressed between HCV-cirrhotic liver tissues from patients with and without hepatocellular carcinoma. (DOCX) [file pone.0040275.s005.docx]

**Supplementary Table S1** Complete list of canonical pathways differentially expressed between HCV-cirrhotic liver tissues from patients with and without HCC

| **Canonical Pathways** | **-log(p-value)** | **Ratio** | **Molecules** |
| --- | --- | --- | --- |
| PI3K/AKT Signaling | 8.46E00 | 2.5E-01 | BAD,PIK3R1,MAPK3,LIMS1,ILK,GSK3A,MAP3K5,CCND1,EIF4E,BCL2,SHC1 (includes EG:20416),IKBKB,HSP90B1,IKBKG,NFKBIA,AKT1,HSP90AB1,MAP2K2,FOXO3,PIK3R2,CTNNB1,MCL1,TP53 (includes EG:22059),ITGB1,CDC37,RHEB,YWHAE,YWHAB,ITGA5,MDM2,NFKB2,BCL2L1,PPP2R1A,PPP2R4,PIK3CD |
| Molecular Mechanisms of Cancer | 7.93E00 | 1.79E-01 | BAD,MAPK3,PIK3R1,NCSTN,CDKN2C,GSK3A,ARHGEF1,RBL1,CCND1,RHOG,RHOB,MAP2K2,TGFB1 (includes EG:21803),HIPK2,FASLG,ADCY9,TP53 (includes EG:22059),CREBBP,ARHGEF17,NFKB2,AURKA,TCF3,RAP1A,APC,BCL2L1,PTPN11,PIK3CD,FZD5,NOTCH1,CDK2,CAMK2G,TCF4,BMPR2,PSENEN,CRK,FZD1,MAP3K5,E2F3,BCL2,EP300,CASP6,SHC1 (includes EG:20416),NFKBIA,NLK,JUN,AKT1,RHOT1,BBC3,ARHGEF2,PIK3R2,CTNNB1,BMP1,PAK4,PAK2,ADCY3,SMAD6,MDM2,BAX,APH1A,NBN,GNAI2,ARHGEF10,PRKCI,FZD4,NF1 (includes EG:18015),PRKAG2,ELK1,CTNND1 |
| PTEN Signaling | 6.98E00 | 2.42E-01 | FOXO4,BAD,PIK3R1,MAPK3,ILK,BMPR2,GSK3A,CCND1,BCL2,SHC1 (includes EG:20416),IKBKB,IKBKG,AKT1,MAP2K2,FOXO3,CSNK2A1,PDGFRA,PIK3R2,FASLG,PDGFRB,EGFR,ITGB1,FGFR1,ITGA5,FGFR2,NFKB2,IGF2R,DDR1,BCL2L1,PIK3CD |
| Apoptosis Signaling | 6.78E00 | 2.71E-01 | BAD,MAPK3,MAP4K4,MAP3K5,BCL2,ACIN1,CASP6,IKBKB,IKBKG,NFKBIA,MAP2K2,FASLG,MCL1,AIFM1,TP53 (includes EG:22059),TNFRSF1A,LMNA,BAX,NFKB2,CDK1,BCL2L1,CAPNS1,CASP2,CAPN1,CAPN2,SPTAN1 |
| Chronic Myeloid Leukemia Signaling | 5.71E00 | 2.38E-01 | BAD,PIK3R1,MAPK3,HDAC10,CRK,E2F3,RBL1,CCND1,CTBP1,IKBKB,IKBKG,AKT1,MAP2K2,TGFB1 (includes EG:21803),HDAC7,CTBP2,PIK3R2,STAT5B,TP53 (includes EG:22059),CRKL,MDM2,NFKB2,BCL2L1,PTPN11,PIK3CD |
| Integrin Signaling | 5.64E00 | 1.95E-01 | MPRIP,ARHGAP26,MAP3K11,PIK3R1,MAPK3,LIMS1,ILK,CRK,TLN1,MYLK,SHC1 (includes EG:20416),ARF6,AKT1,RHOG,RHOB,MAP2K2,RHOT1,PIK3R2,ITGB4,TSPAN4,ACTN1,ITGB1,PAK4,PAK2,CRKL,ACTB,ITGA5,RAP1A,ARHGAP5,MYL9,MYL12A,TLN2,CAPNS1,ARF3,WAS,CAPN1,ZYX,CAPN2,PIK3CD,ACTN4,ITGA7 |
| Androgen Signaling | 5.49E00 | 1.94E-01 | POLR2D,CALM1 (includes others),MAPK3,POLR2J,CCND1,EP300,GNB1,HSPA4,SHC1 (includes EG:20416),TGFB1I1,POLR2A,JUN,AR,NCOA2,NCOA4,CALR,CREBBP,NFKB2,GNG10,GNAI2,KAT2B,PRKCI,POLR2E,GTF2E1,NCOA1,GNB2,PRKAG2,GTF2H1 |
| Acute Phase Response Signaling | 5.38E00 | 2.02E-01 | IL6ST,SOCS3,SOCS1,TCF4,saa3p,PIK3R1,APOA2,MAPK3,SOCS6,AMBP,CP,MAP3K5,FGG,IKBKB,SHC1 (includes EG:20416),IKBKG,IL36G,NFKBIA,JUN,AKT1,MAP2K2,APCS,FGB (includes EG:110135),LBP,PIK3R2,TTR,TNFRSF1A,AHSG,IL36A,NFKB2,TCF3,ALB,TF,PTPN11,PIK3CD,ELK1 |
| p53 Signaling | 5.26E00 | 2.5E-01 | TP53 (includes EG:22059),GADD45B,PIK3R1,CSNK1D,MDM2,BAX,CCND1,TP53I3,EP300,BCL2,CASP6,BCL2L1,KAT2B,JUN,AKT1,BBC3,STAG1,ADCK3,PIK3CD,PIK3R2,PML,HIPK2,CTNNB1,CDK2 |
| PPAR Signaling | 5E00 | 2.24E-01 | TNFRSF1A,PDGFA,MAPK3,CREBBP,IL36A,MAP4K4,NFKB2,PDGFB,EP300,SHC1 (includes EG:20416),IKBKB,IL36G,IKBKG,HSP90B1,JUN,NFKBIA,MAP2K2,HSP90AB1,PDGFRA,NCOA1,IL1RAPL1,STAT5B,CITED2,PDGFRB |
| Renal Cell Carcinoma Signaling | 5E00 | 2.7E-01 | RBX1,PAK4,PAK2,PIK3R1,MAPK3,CREBBP,CRK,RAP1A,PDGFB,EP300,VEGFA,AKT1,JUN,MAP2K2,PTPN11,CUL2,TGFB1 (includes EG:21803),PIK3CD,PIK3R2,FH |
| Wnt/β-catenin Signaling | 4.85E00 | 2.01E-01 | TCF4,CSNK1G1,SOX15,CSNK1A1,ILK,GSK3A,FZD1,CCND1,EP300,JUN,AKT1,NLK,TGFB1 (includes EG:21803),RARB,CSNK2A1,SFRP5,MAP4K1,CTNNB1,TP53 (includes EG:22059),SOX4,APPL2,CSNK1G3,CREBBP,CSNK1D,MDM2,TCF3,APC,PPP2R1A,FZD4,PPP2R4,NR5A2,FZD5,PIN1,ACVR2A,TCF7L2 |
| Glucocorticoid Receptor Signaling | 4.72E00 | 1.63E-01 | TAF11 (includes EG:309638),POLR2D,HSPA1A/HSPA1B,MAPK3,PIK3R1,POLR2J,TAF13 (includes EG:310784),FGG,BCL2,EP300,HSPA4,SHC1 (includes EG:20416),IKBKB,IKBKG,HSP90B1,NFKBIA,AR,POLR2A,NFAT5,AKT1,JUN,HSP90AB1,NCOA2,CCL2,MAP2K2,TGFB1 (includes EG:21803),BAG1,PPP3R1,FOXO3,PIK3R2,STAT5B,TAF6,HSPA9,CREBBP,NFATC1,PPP3CC,KAT2B,BCL2L1,SUMO1,DUSP1,SMARCA2,POLR2E,GTF2E1,NCOA1,PRKAG2,GTF2H1,PIK3CD,ELK1 |
| PAK Signaling | 4.4E00 | 2.06E-01 | ITGB1,PAK4,PAK2,ARHGAP10,MYL6,CFL1,PDGFA,PIK3R1,MAPK3,PAK1IP1,ITGA5,PDGFB,MYLK,MYL9,SHC1 (includes EG:20416),MYL12A,MAP2K2,PDGFRA,PIK3CD,PIK3R2,MYL10,PDGFRB |
| Prostate Cancer Signaling | 4.37E00 | 2.16E-01 | TP53 (includes EG:22059),BAD,SRD5A1,MAPK3,PIK3R1,CREBBP,MDM2,NFKB2,CCND1,BCL2,HSP90B1,AKT1,NFKBIA,AR,MAP2K2,HSP90AB1,NKX3-1,PIK3CD,PIK3R2,CTNNB1,CDK2 |
| Role of Osteoblasts, Osteoclasts and Chondrocytes in Rheumatoid Arthritis | 4.35E00 | 1.71E-01 | TCF4,CALM1 (includes others),BAD,PIK3R1,MAPK3,CSNK1A1,BMPR2,MAP3K5,FZD1,BCL2,IKBKB,IL36G,IKBKG,NFKBIA,NFAT5,AKT1,JUN,RUNX2,TGFB1 (includes EG:21803),PPP3R1,SFRP5,PIK3R2,CTNNB1,BMP1,ITGB1,TNFRSF1A,IL36A,SMAD6,ITGA5,NFATC1,PPP3CC,TCF3,CSF1R,APC,COL1A1,FZD4,CSF1 (includes EG:12977),FZD5,PIK3CD,IL1RAPL1,TCF7L2 |
| Phospholipase C Signaling | 4.3E00 | 1.65E-01 | MPRIP,TRD@,CALM1 (includes others),MYL6,MAPK3,HDAC10,ARHGEF1,CD79A,EP300,GNB1,SHC1 (includes EG:20416),NFAT5,RHOG,PLCE1,RHOB,MAP2K2,RHOT1,PPP3R1,GPLD1,HDAC7,ARHGEF2,MYL10,ADCY9,ITGB1,PLD3,ITPR2,ADCY3,CREBBP,MEF2A,ITGA5,NFATC1,ARHGEF17,PPP3CC,NFKB2,RAP1A,GNG10,MYL9,MYL12A,PRKCI,ARHGEF10,MEF2D,ITPR3,GNB2 |
| Hepatic Fibrosis / Hepatic Stellate Cell Activation | 4.23E00 | 1.97E-01 | IGFBP4,MYH10,MYL6,PDGFA,BCL2,VEGFA,CCL2,TGFB1 (includes EG:21803),TIMP1,PDGFRA,LBP,FASLG,EGFR,PDGFRB,TNFRSF1A,FGFR1,FGFR2,MMP2,BAX,NFKB2,IFNAR2,PDGFB,FGF1,MYL9,COL1A1,CSF1 (includes EG:12977),MYH9,IL1RAPL1,IFNAR1 |
| Estrogen Receptor Signaling | 4.18E00 | 1.99E-01 | TAF11 (includes EG:309638),POLR2D,MAPK3,POLR2J,H3F3A/H3F3B,TAF13 (includes EG:310784),EP300,CTBP1,SPEN,SHC1 (includes EG:20416),CDK8,POLR2A,MAP2K2,NCOA2,MED15,CTBP2,TAF6,CREBBP,MED6 (includes EG:10001),KAT2B,POLR2E,MED13L,GTF2E1,NCOA1,GTF2H1,MED4 (includes EG:29079),CARM1 |
| Erythropoietin Signaling | 4.08E00 | 2.31E-01 | EPO,SOCS1,SOCS3,PTPN6,EPOR,MAPK3,PIK3R1,NFKB2,SHC1 (includes EG:20416),NFKBIA,PRKCI,AKT1,JUN,MAP2K2,PIK3CD,PIK3R2,STAT5B,ELK1 |
| Huntington's Disease Signaling | 4.01E00 | 1.68E-01 | POLR2D,HSPA1A/HSPA1B,REST,MAPK3,PIK3R1,POLR2J,HDAC10,NGF,EP300,GNB1,HSPA4,CASP6,SHC1 (includes EG:20416),ARFIP2,POLR2A,AKT1,JUN,CPLX2,HDAC7,VAMP3,PIK3R2,NAPA,EGFR,TP53 (includes EG:22059),HSPA9,CREBBP,BAX,GNG10,GRM5,RCOR1,BCL2L1,PRKCI,CAPNS1,POLR2E,CASP2,CAPN1,GNB2,STX16,CAPN2,PIK3CD |
| Glioblastoma Multiforme Signaling | 4E00 | 1.83E-01 | PDGFA,PIK3R1,MAPK3,FZD1,E2F3,CCND1,SHC1 (includes EG:20416),RHOG,AKT1,PLCE1,MAP2K2,RHOB,RHOT1,PDGFRA,PIK3R2,CTNNB1,EGFR,PDGFRB,TP53 (includes EG:22059),ITPR2,MDM2,TCF3,PDGFB,APC,FZD4,NF1 (includes EG:18015),ITPR3,FZD5,PIK3CD,CDK2 |
| NRF2-mediated Oxidative Stress Response | 3.99E00 | 1.77E-01 | AKR7A2,RBX1,PPIB,PIK3R1,MAPK3,DNAJC3,MAP3K5,SOD3,EP300,JUN,AKT1,MAP2K2,DNAJC8,PMF1,FOSL1,DNAJA2,JUND,TXN (includes EG:116484),PIK3R2,NFE2L2,DNAJC9,ACTB,CREBBP,SLC35A2,JUNB,DNAJB9,DNAJB14,PRKCI,STIP1,CAT,PIK3CD,PTPLAD1,DNAJB5,EPHX1 |
| FAK Signaling | 3.99E00 | 2.06E-01 | ITGB1,PAK4,PAK2,ARHGAP26,ACTB,PIK3R1,MAPK3,ITGA5,CRK,TLN1,CAPNS1,TLN2,AKT1,MAP2K2,WAS,CAPN1,CAPN2,PIK3CD,PIK3R2,TNS1,EGFR |
| Reelin Signaling in Neurons | 3.85E00 | 2.32E-01 | ITGB1,MAP3K11,PIK3R1,CRKL,MAPK8IP2,ITGA5,ARHGEF1,RELN,MAPK8IP3,FRK,APP,APBB1,YES1,AKT1,ARHGEF10,MAP4K1,ARHGEF2,PIK3CD,PIK3R2 |
| Hereditary Breast Cancer Signaling | 3.83E00 | 1.94E-01 | POLR2D,GADD45B,PIK3R1,POLR2J,HDAC10,DDB2,CCND1,RAD50,EP300,POLR2A,AKT1,HDAC7,RFC2,PIK3R2,TP53 (includes EG:22059),CREBBP,RFC5,CDK1,NBN,PALB2,SMARCA2,POLR2E,H2AFX,PIK3CD,RFC3 |
| JAK/Stat Signaling | 3.8E00 | 2.5E-01 | STAT6,SOCS1,SOCS3,PTPN6,PIAS2,MAPK3,PIK3R1,SOCS6,SHC1 (includes EG:20416),AKT1,PTPN11,MAP2K2,STAT2,PIK3CD,PIK3R2,STAT5B |
| Neuregulin Signaling | 3.77E00 | 2.06E-01 | ITGB1,BAD,PIK3R1,MAPK3,CRKL,ITGA5,CRK,SHC1 (includes EG:20416),ERBB2IP,HSP90B1,PRKCI,AKT1,PICK1,MAP2K2,PTPN11,HSP90AB1,PIK3R2,STAT5B,ELK1,EGFR,MATK |
| Clathrin-mediated Endocytosis Signaling | 3.75E00 | 1.8E-01 | RAB4A,PDGFA,PIK3R1,GAK,VEGFA,CD2AP,ARF6,AP1G2,RAB11B,PPP3R1,CSNK2A1,PIK3R2,SH3GLB2,ITGB4,HGS,ITGB1,MYO6,AP2M1,EPN1,ACTB,ITGA5,MDM2,PPP3CC,PDGFB,FGF1,FGF21,ARRB2,TF,PIP5K1C,CLTA,PIK3CD |
| Glioma Signaling | 3.75E00 | 1.96E-01 | TP53 (includes EG:22059),CALM1 (includes others),PDGFA,PIK3R1,MAPK3,CDKN2C,MDM2,E2F3,RBL1,CCND1,IGF2R,PDGFB,SHC1 (includes EG:20416),AKT1,PRKCI,MAP2K2,PDGFRA,PIK3CD,PIK3R2,EGFR,PDGFRB,CAMK2G |
| IL-6 Signaling | 3.7E00 | 2.1E-01 | IL6ST,SOCS1,TNFRSF1A,MAPK3,IL36A,MAP4K4,NFKB2,COL1A1,SHC1 (includes EG:20416),IKBKB,IKBKG,IL36G,JUN,NFKBIA,MAP2K2,PTPN11,CSNK2A1,IL1RAPL1,LBP,ELK1,HSPB1 |
| Role of NFAT in Regulation of the Immune Response | 3.68E00 | 1.65E-01 | CSNK1G1,CALM1 (includes others),TRD@,PIK3R1,CD4,MAPK3,CSNK1A1,GSK3A,CD79A,CABIN1,GNB1,IKBKB,IKBKG,NFKBIA,JUN,AKT1,NFAT5,MAP2K2,PPP3R1,PIK3R2,ITPR2,CSNK1G3,CSNK1D,MEF2A,NFATC1,PPP3CC,NFKB2,GNG10,GNAI2,MEF2D,ITPR3,GNB2,PIK3CD |
| IGF-1 Signaling | 3.68E00 | 2.06E-01 | IGFBP4,SOCS1,SOCS3,YWHAE,BAD,YWHAB,MAPK3,PIK3R1,SOCS6,SHC1 (includes EG:20416),PRKCI,AKT1,JUN,MAP2K2,PTPN11,FOXO3,CSNK2A1,PRKAG2,PIK3CD,PIK3R2,ELK1,CYR61 |
| SAPK/JNK Signaling | 3.63E00 | 2.06E-01 | TP53 (includes EG:22059),MAP3K11,DUSP8,TRD@,PIK3R1,CRKL,MAPK8IP2,MAP4K4,CRK,NFATC1,MAP3K5,MAPK8IP3,GNB1,SHC1 (includes EG:20416),MINK1,JUN,DUSP10,MAP4K1,PIK3CD,PIK3R2,ELK1 |
| Germ Cell-Sertoli Cell Junction Signaling | 3.49E00 | 1.8E-01 | MAP3K11,PIK3R1,MAPK3,ILK,MAP3K5,TUBB,TUBB2B,AGGF1,AKT1,RHOG,MAP2K2,RHOB,RHOT1,TGFB1 (includes EG:21803),MTMR2,PIK3R2,JUP,CTNNB1,ACTN1,ITGB1,EPN1,PAK4,PAK2,TUBB2C,TNFRSF1A,ACTB,ZYX,PIK3CD,ACTN4,CTNND1 |
| IL-3 Signaling | 3.48E00 | 2.3E-01 | STAT6,PTPN6,BAD,MAPK3,CRKL,PIK3R1,PPP3CC,SHC1 (includes EG:20416),PRKCI,AKT1,JUN,MAP2K2,PPP3R1,PIK3CD,PIK3R2,STAT5B,ELK1 |
| RAR Activation | 3.43E00 | 1.71E-01 | PIK3R1,ADH1C (includes EG:11522),MAP3K5,EP300,VEGFA,ALDH1A1,AKT1,JUN,TGFB1 (includes EG:21803),RARB,CSNK2A1,SORBS3,PIK3R2,STAT5B,CITED2,ADCY9,RDH14,RDH11,CREBBP,ADCY3,SMAD6,NFKB2,KAT2B,PRKCI,SMARCA2,DUSP1,NCOA1,PRKAG2,GTF2H1,PIK3CD,PML,CARM1 |
| B Cell Receptor Signaling | 3.36E00 | 1.79E-01 | MAP3K11,BAD,CALM1 (includes others),PIK3R1,MAPK3,GSK3A,MAP3K5,CD79A,SHC1 (includes EG:20416),IKBKB,IKBKG,AKT1,JUN,NFAT5,NFKBIA,MAP2K2,PPP3R1,CD22,PIK3R2,PTPN6,NFATC1,PPP3CC,NFKB2,BCL2L1,PTPN11,PIK3CD,ELK1,CAMK2G |
| Hypoxia Signaling in the Cardiovascular System | 3.3E00 | 2.35E-01 | TP53 (includes EG:22059),EPO,CSNK1D,MDM2,UBE2D1,EP300,VEGFA,HSP90B1,AKT1,JUN,NFKBIA,SUMO1,HSP90AB1,UBE2B,UBE2G1,UBE2J1 |
| IL-2 Signaling | 3.25E00 | 2.41E-01 | SOCS1,IL2RG,MAPK3,PIK3R1,SHC1 (includes EG:20416),AKT1,JUN,MAP2K2,PTPN11,CSNK2A1,PIK3CD,PIK3R2,ELK1,STAT5B |
| Pancreatic Adenocarcinoma Signaling | 3.25E00 | 1.85E-01 | TP53 (includes EG:22059),PLD3,BAD,PIK3R1,MAPK3,MDM2,NFKB2,E2F3,CCND1,BCL2,VEGFA,BCL2L1,AKT1,MAP2K2,TGFB1 (includes EG:21803),GPLD1,PIK3CD,PIK3R2,ELK1,NOTCH1,CDK2,EGFR |
| Thrombin Signaling | 3.23E00 | 1.64E-01 | MPRIP,MYL6,PIK3R1,MAPK3,ARHGEF1,MYLK,GNB1,SHC1 (includes EG:20416),IKBKB,RHOG,AKT1,PLCE1,RHOB,MAP2K2,RHOT1,ARHGEF2,PIK3R2,MYL10,EGFR,ADCY9,ITPR2,ADCY3,NFKB2,GNG10,MYL9,GNAI2,MYL12A,ARHGEF10,PRKCI,ITPR3,GNB2,PIK3CD,ELK1,CAMK2G |
| Extrinsic Prothrombin Activation Pathway | 3.2E00 | 3.5E-01 | SERPINC1,PROS1,F5,F13B,FGB (includes EG:110135),TFPI,FGG |
| Protein Kinase A Signaling | 3.17E00 | 1.49E-01 | MYH10,BAD,MYL6,MAPK3,GSK3A,MYLK,GNB1,PLCE1,MAP2K2,TGFB1 (includes EG:21803),PPP3R1,MYL10,ADCY9,YWHAE,YWHAB,ITPR2,PDE9A,CREBBP,PPP1R11,NFKB2,PPP3CC,TCF3,RAP1A,GNG10,MYL9,AKAP13,MYL12A,ITPR3,GNB2,CAMK2G,ANAPC2,TCF4,CALM1 (includes others),H3F3A/H3F3B,AKAP7,NFAT5,NFKBIA,FLNA,CTNNB1,ADCY3,PYGL,NFATC1,GNAI2,PRKCI,PRKAG2,ADD1,ELK1,TCF7L2,CDC27 |
| GM-CSF Signaling | 3.11E00 | 2.24E-01 | MAPK3,PIK3R1,PPP3CC,CCND1,SHC1 (includes EG:20416),BCL2L1,AKT1,MAP2K2,PTPN11,PPP3R1,PIK3CD,PIK3R2,ELK1,STAT5B,CAMK2G |
| Role of CHK Proteins in Cell Cycle Checkpoint Control | 3.1E00 | 2.86E-01 | TP53 (includes EG:22059),RAD17 (includes EG:19356),RFC2,RFC5,E2F3,CDK1,RAD50,CDK2,RFC3,NBN |
| Role of Tissue Factor in Cancer | 3.08E00 | 1.93E-01 | TP53 (includes EG:22059),ITGB1,CFL1,PIK3R1,MAPK3,PLAUR,FRK,EIF4E,FGG,VEGFA,BCL2L1,YES1,ARRB2,AKT1,PTPN11,CSF1 (includes EG:12977),FGB (includes EG:110135),PIK3CD,PIK3R2,STAT5B,CYR61,EGFR |
| Actin Cytoskeleton Signaling | 3.05E00 | 1.51E-01 | MYH10,PFN1,MPRIP,MYL6,PDGFA,PIK3R1,MAPK3,ARHGEF1,CRK,MYLK,SHC1 (includes EG:20416),IQGAP2,MAP2K2,LBP,PIK3R2,MYL10,ACTN1,MATK,ITGB1,PAK4,PAK2,CFL1,ACTB,CRKL,ITGA5,PDGFB,APC,FGF1,MYL9,FGF21,MYL12A,PIP5K1C,WAS,MYH9,PIK3CD,ACTN4 |
| Protein Ubiquitination Pathway | 3.04E00 | 1.53E-01 | PSMB3,B2M,ANAPC2,USP24,RBX1,CRYAB,USP5,HSPA1A/HSPA1B,UBE3B,UBR2 (includes EG:224826),DNAJC3,HSPA4,HSP90B1,HSP90AB1,UBE2B,BAG1,PSMC6,DNAJC8,UCHL5,NEDD4L,PSMA2,DNAJC22,UBE2J1,AMFR,HSPB6,DNAJC9,USP15,HSPA9,MDM2,DNAJB9,USP33,DNAJB14,UBE2D1,FZR1,CUL2,UBE2G1,PSMA4,USP46,UBA1,USP25,DNAJB5,HSPB1 |
| Polyamine Regulation in Colon Cancer | 3.02E00 | 2.76E-01 | TCF4,AZIN1,PSMF1,SAT1,PSME4,CTNNB1,PSME3,APC |
| ILK Signaling | 3.01E00 | 1.66E-01 | MYH10,MYL6,PIK3R1,MAPK3,ILK,GSK3A,CCND1,VEGFA,TGFB1I1,RHOG,AKT1,JUN,RHOB,FLNA,RHOT1,PIK3R2,ITGB4,CTNNB1,ACTN1,ITGB1,CFL1,TNFRSF1A,ACTB,FERMT2,SNAI1,NFKB2,MYL9,PPP2R1A,PPP2R4,MYH9,PIK3CD,ACTN4 |
| ATM Signaling | 2.98E00 | 2.41E-01 | TP53 (includes EG:22059),SMC3,GADD45B,MDM2,CDK1,RAD50,SMC1A,NBN,JUN,NFKBIA,SMC2,H2AFX,CDK2 |
| Amyloid Processing | 2.98E00 | 2.32E-01 | MAPK3,CSNK1D,CSNK1A1,NCSTN,PSENEN,APP,APH1A,AKT1,CAPNS1,CAPN1,CSNK2A1,PRKAG2,CAPN2 |
| Role of NFAT in Cardiac Hypertrophy | 2.97E00 | 1.54E-01 | IL6ST,LIF,CALM1 (includes others),PIK3R1,MAPK3,CSNK1A1,HDAC10,EP300,CABIN1,GNB1,SHC1 (includes EG:20416),AKT1,PLCE1,MAP2K2,TGFB1 (includes EG:21803),PPP3R1,HDAC7,PIK3R2,ADCY9,ITPR2,ADCY3,MEF2A,PPP3CC,GNG10,GNAI2,PRKCI,MEF2D,ITPR3,GNB2,PRKAG2,PIK3CD,CAMK2G |
| TR/RXR Activation | 2.96E00 | 1.98E-01 | AKR1C1/AKR1C2,GPS2,PIK3R1,BCL3,MDM2,ME1,EP300,KLF9,SLC16A3,UCP3,AKT1,NCOA2,SREBF2,DIO1,NCOA1,NCOA4,PIK3CD,PIK3R2,SYT12 |
| Colorectal Cancer Metastasis Signaling | 2.94E00 | 1.51E-01 | IL6ST,TCF4,BAD,PIK3R1,MAPK3,FZD1,CCND1,VEGFA,GNB1,RHOG,AKT1,JUN,RHOB,MMP25,MAP2K2,RHOT1,TGFB1 (includes EG:21803),PIK3R2,CTNNB1,EGFR,MMP19,TP53 (includes EG:22059),ADCY9,TNFRSF1A,ADRBK2,ADCY3,MMP2,BAX,NFKB2,TCF3,APC,GNG10,BCL2L1,FZD4,GNB2,PRKAG2,FZD5,PIK3CD,TCF7L2 |
| Death Receptor Signaling | 2.92E00 | 2.15E-01 | TNFRSF1A,MAP4K4,MAP3K5,NFKB2,BCL2,TANK,CASP6,IKBKB,IKBKG,NFKBIA,TNFSF12,CASP2,FASLG,HSPB1 |
| VEGF Signaling | 2.9E00 | 1.92E-01 | EIF2S3,EIF2B4,YWHAE,BAD,ACTB,MAPK3,PIK3R1,EIF1,BCL2,VEGFA,SHC1 (includes EG:20416),BCL2L1,AKT1,MAP2K2,FOXO3,PIK3CD,PIK3R2,ACTN4,ACTN1 |
| Role of JAK2 in Hormone-like Cytokine Signaling | 2.88E00 | 2.7E-01 | EPO,SHC1 (includes EG:20416),SOCS1,SOCS3,PTPN6,PTPN11,EPOR,SOCS6,STAT5B,SIRPA |
| fMLP Signaling in Neutrophils | 2.87E00 | 1.72E-01 | CALM1 (includes others),ITPR2,MAPK3,PIK3R1,NFATC1,NFKB2,PPP3CC,GNG10,GNB1,GNAI2,NFKBIA,NFAT5,PRKCI,MAP2K2,WAS,PPP3R1,ITPR3,GNB2,CYBB,PIK3CD,PIK3R2,ELK1 |
| Angiopoietin Signaling | 2.82E00 | 2.03E-01 | PAK4,PAK2,GRB14,BAD,PIK3R1,CRK,NFKB2,IKBKB,IKBKG,NFKBIA,AKT1,PTPN11,PIK3CD,PIK3R2,STAT5B |
| Endometrial Cancer Signaling | 2.82E00 | 2.28E-01 | TP53 (includes EG:22059),BAD,MAPK3,PIK3R1,ILK,CCND1,AKT1,MAP2K2,FOXO3,PIK3CD,PIK3R2,ELK1,CTNNB1 |
| CXCR4 Signaling | 2.81E00 | 1.66E-01 | MYL6,PIK3R1,MAPK3,CD4,CRK,GNB1,RHOG,AKT1,JUN,MAP2K2,RHOB,RHOT1,PIK3R2,MYL10,ADCY9,PAK4,PAK2,ITPR2,ADCY3,GNG10,GNAI2,MYL9,MYL12A,PRKCI,ITPR3,GNB2,PIK3CD,ELK1 |
| Breast Cancer Regulation by Stathmin1 | 2.81E00 | 1.57E-01 | CALM1 (includes others),PIK3R1,MAPK3,ARHGEF1,E2F3,TUBB,TUBB2B,GNB1,SHC1 (includes EG:20416),MAP2K2,RB1CC1,ARHGEF2,PIK3R2,TP53 (includes EG:22059),ADCY9,TUBB2C,ITPR2,ADCY3,ARHGEF17,PPP1R11,CDK1,GNG10,GNAI2,PPP2R1A,ARHGEF10,PRKCI,PPP2R4,ITPR3,GNB2,PRKAG2,PIK3CD,CDK2,CAMK2G |
| Axonal Guidance Signaling | 2.8E00 | 1.32E-01 | GLI2,PFN1,MYL6,MAPK3,PIK3R1,TUBB,VEGFA,GNB1,MAP2K2,PPP3R1,PLXNB2,MYL10,CFL1,CRKL,ITGA5,PPP3CC,RAP1A,PDGFB,GNG10,MYL9,MYL12A,PTPN11,RTN4,GNB2,PIK3CD,FZD5,PDGFA,SEMA6A,CRK,FZD1,NGF,EIF4E,TUBB2B,EPHB6,SHC1 (includes EG:20416),AKT1,NFAT5,PLXNA1,SDC2,EFNB1,PIK3R2,SHANK2,BMP1,ITGB1,EPHB4,PAK4,PAK2,NRP2,ADAM2,TUBB2C,NFATC1,PLXND1,GNAI2,PRKCI,FZD4,WAS,PRKAG2 |
| RANK Signaling in Osteoclasts | 2.79E00 | 1.89E-01 | MAP3K11,CALM1 (includes others),MAPK3,PIK3R1,NFATC1,PPP3CC,MAP3K5,NFKB2,IKBKB,IKBKG,NFKBIA,AKT1,JUN,MAP2K2,PPP3R1,PIK3CD,PIK3R2,ELK1 |
| Macropinocytosis Signaling | 2.75E00 | 1.97E-01 | ITGB1,PDGFA,PIK3R1,ITGA5,NGF,CSF1R,PDGFB,ARF6,PRKCI,ABI1,CSF1 (includes EG:12977),PIK3CD,ITGB4,PIK3R2,ACTN4 |
| Role of Macrophages, Fibroblasts and Endothelial Cells in Rheumatoid Arthritis | 2.73E00 | 1.38E-01 | IL6ST,SOCS1,SOCS3,TCF4,CALM1 (includes others),PDGFA,MAPK3,PIK3R1,IL32,CSNK1A1,FZD1,CCND1,VEGFA,IKBKB,IKBKG,IL36G,C5AR1,NFKBIA,NLK,NFAT5,AKT1,JUN,PLCE1,CCL2,MAP2K2,TGFB1 (includes EG:21803),PPP3R1,SFRP5,PIK3R2,LTBR,CTNNB1,TNFRSF1A,IL36A,NFATC1,PPP3CC,TCF3,PDGFB,APC,PRKCI,FZD4,CSF1 (includes EG:12977),FZD5,PIK3CD,IL1RAPL1,TCF7L2,CAMK2G |
| iCOS-iCOSL Signaling in T Helper Cells | 2.7E00 | 1.72E-01 | IL2RG,BAD,CALM1 (includes others),TRD@,ITPR2,CD4,PIK3R1,NFATC1,NFKB2,PPP3CC,SHC1 (includes EG:20416),IKBKB,IKBKG,AKT1,NFAT5,NFKBIA,PPP3R1,ITPR3,PIK3CD,PIK3R2,CAMK2G |
| Intrinsic Prothrombin Activation Pathway | 2.7E00 | 2.65E-01 | COL1A1,KNG1,KLK1,SERPINC1,PROS1,F5,F13B,FGB (includes EG:110135),FGG |
| Lymphotoxin β Receptor Signaling | 2.66E00 | 2.13E-01 | MAPK3,PIK3R1,CREBBP,NFKB2,EP300,IKBKB,BCL2L1,IKBKG,AKT1,NFKBIA,PIK3CD,LTBR,PIK3R2 |
| HIF1α Signaling | 2.64E00 | 1.85E-01 | TP53 (includes EG:22059),EPO,EGLN2,RBX1,MAPK3,PIK3R1,CREBBP,MMP2,MDM2,EP300,P4HTM,VEGFA,JUN,AKT1,MMP25,CUL2,NCOA1,PIK3CD,PIK3R2,MMP19 |
| Acute Myeloid Leukemia Signaling | 2.61E00 | 1.95E-01 | TCF4,BAD,MAPK3,PIK3R1,NFKB2,TCF3,CCND1,CSF1R,AKT1,MAP2K2,PIK3CD,JUP,PIK3R2,PML,STAT5B,TCF7L2 |
| Lysine Degradation | 2.61E00 | 1.22E-01 | SETD8,AASDHPPT,ACAT2,RDH11,ELOVL6,EP300,KAT2B,ALDH1A1,SMYD3,EHMT2,ACAT1,DBT,EHHADH,SHMT2,PLOD3,HADH,ALDH7A1 |
| PPARα/RXRα Activation | 2.61E00 | 1.56E-01 | CYP2C9,MAPK3,APOA2,MAP4K4,ADIPOR1,ABCA1,EP300,SHC1 (includes EG:20416),IKBKB,IKBKG,HSP90B1,JUN,PLCE1,NFKBIA,HSP90AB1,MAP2K2,TGFB1 (includes EG:21803),CLOCK,STAT5B,ADCY9,ACOX1,CREBBP,ADCY3,CD36,NFKB2,CAND1,PRKAG2,IL1RAPL1,ACVR2A |
| Calcium Signaling | 2.6E00 | 1.45E-01 | MYH10,MYL6,CALM1 (includes others),ATP2B1,MAPK3,HDAC10,EP300,GRINA,CABIN1,NFAT5,TRPV6,PPP3R1,HDAC7,ASPH,TPM4,CALR,ATP2C1,ITPR2,CREBBP,MEF2A,NFATC1,PPP3CC,RAP1A,ATP2B2,MYL9,MEF2D,ITPR3,PRKAG2,MYH9,CAMK2G |
| PI3K Signaling in B Lymphocytes | 2.58E00 | 1.68E-01 | CALM1 (includes others),ITPR2,MAPK3,PIK3R1,NFATC1,NFKB2,PPP3CC,CD79A,IKBKB,IKBKG,PLCE1,JUN,AKT1,NFKBIA,NFAT5,PRKCI,MAP2K2,PPP3R1,FOXO3,ITPR3,PIK3CD,PIK3R2,ELK1,CAMK2G |
| CD28 Signaling in T Helper Cells | 2.58E00 | 1.67E-01 | PTPN6,CALM1 (includes others),TRD@,ITPR2,CD4,PIK3R1,NFATC1,NFKB2,PPP3CC,IKBKB,IKBKG,JUN,AKT1,NFKBIA,NFAT5,MAP2K2,PTPN11,WAS,PPP3R1,ITPR3,PIK3CD,PIK3R2 |
| Role of Wnt/GSK-3β Signaling in the Pathogenesis of Influenza | 2.55E00 | 1.98E-01 | TCF4,CSNK1G1,CSNK1G3,CSNK1A1,CSNK1D,FZD1,TCF3,APC,FZD4,NCOA2,NCOA1,NCOA4,FZD5,CTNNB1,TCF7L2,IFNAR1 |
| Human Embryonic Stem Cell Pluripotency | 2.54E00 | 1.56E-01 | TCF4,PDGFA,PIK3R1,FGFR1,SMAD6,FGFR2,BMPR2,GSK3A,FZD1,NGF,TCF3,APC,PDGFB,AKT1,FZD4,TGFB1 (includes EG:21803),PDGFRA,FZD5,PIK3CD,PIK3R2,CTNNB1,TCF7L2,BMP1,PDGFRB |
| Cardiac Hypertrophy Signaling | 2.54E00 | 1.47E-01 | EIF2B4,MAP3K11,CALM1 (includes others),MYL6,PIK3R1,MAPK3,MAP3K5,EIF4E,EP300,GNB1,AKT1,JUN,PLCE1,RHOG,RHOB,MAP2K2,RHOT1,TGFB1 (includes EG:21803),PPP3R1,PIK3R2,MYL10,ADCY9,CREBBP,ADCY3,MEF2A,PPP3CC,GNG10,MYL9,GNAI2,MYL12A,MEF2D,GNB2,PRKAG2,PIK3CD,ELK1,HSPB1 |
| NF-κB Signaling | 2.54E00 | 1.59E-01 | AZI2,TRD@,PIK3R1,BMPR2,MAP4K4,NGF,EP300,TANK,IKBKB,IKBKG,IL36G,NFKBIA,AKT1,PDGFRA,CSNK2A1,LTBR,PIK3R2,EGFR,PDGFRB,TNFRSF1A,FGFR1,CREBBP,IL36A,FGFR2,NFKB2,IGF2R,DDR1,PIK3CD |
| Ephrin Receptor Signaling | 2.53E00 | 1.45E-01 | PDGFA,MAPK3,MAP4K4,CRK,GRINA,EPHB6,VEGFA,GNB1,SHC1 (includes EG:20416),AKT1,MAP2K2,SDC2,EFNB1,ITGB1,EPHB4,PAK4,PAK2,CFL1,CRKL,ITGA5,RAP1A,PDGFB,FGF1,GNG10,GNAI2,PTPN11,ABI1,WAS,GNB2 |
| Docosahexaenoic Acid (DHA) Signaling | 2.52E00 | 2.24E-01 | BCL2L1,AKT1,BAD,PIK3R1,GSK3A,PIK3CD,PIK3R2,BAX,PNPLA2,APP,BCL2 |
| Regulation of Actin-based Motility by Rho | 2.5E00 | 1.87E-01 | PAK4,MPRIP,PAK2,PFN1,MYL6,CFL1,ACTB,MYLK,MYL9,MYL12A,RHOG,RHOB,WAS,PIP5K1C,RHOT1,ARHGDIA,MYL10 |
| IL-8 Signaling | 2.49E00 | 1.5E-01 | PIK3R1,MAPK3,MAP4K4,CCND1,BCL2,VEGFA,GNB1,IKBKB,IKBKG,RHOG,AKT1,MAP2K2,RHOB,RHOT1,GPLD1,CYBB,PIK3R2,EGFR,PLD3,PAK2,MPO,MMP2,BAX,GNG10,GNAI2,BCL2L1,PRKCI,GNB2,PIK3CD |
| Production of Nitric Oxide and Reactive Oxygen Species in Macrophages | 2.47E00 | 1.44E-01 | MAP3K11,MAPK3,PIK3R1,MAP3K5,IKBKB,IKBKG,NFKBIA,AKT1,JUN,RHOG,RHOB,RHOT1,CYBB,PIK3R2,PTPN6,TNFRSF1A,CREBBP,MPO,PPP1R11,NFKB2,RAP1A,PPP2R1A,PRKCI,PPP2R4,CAT,PIK3CD,SIRPA |
| Cleavage and Polyadenylation of Pre-mRNA | 2.43E00 | 4.17E-01 | PAPOLA,CSTF1,NUDT21,CPSF1,CSTF3 |
| Tight Junction Signaling | 2.4E00 | 1.59E-01 | MYH10,MYL6,CPSF1,MYLK,MPDZ,AKT1,JUN,CLDN4,TGFB1 (includes EG:21803),ARHGEF2,CTNNB1,CSTF1,TNFRSF1A,ACTB,CSDA,NFKB2,MYL9,PPP2R1A,PRKCI,PPP2R4,NUDT21,PRKAG2,MYH9,SPTAN1,CSTF3,CLDN3 |
| ERK/MAPK Signaling | 2.39E00 | 1.47E-01 | BAD,PIK3R1,MAPK3,H3F3A/H3F3B,TLN1,CRK,RAPGEF4,EIF4E,SHC1 (includes EG:20416),MAP2K2,PIK3R2,ITGB1,PAK4,PAK2,YWHAB,CRKL,ITGA5,NFATC1,PPP1R11,RAP1A,ELF2,PPP2R1A,TLN2,PRKCI,DUSP1,PPP2R4,PRKAG2,PIK3CD,ELK1,HSPB1 |
| CREB Signaling in Neurons | 2.39E00 | 1.44E-01 | POLR2D,CALM1 (includes others),PIK3R1,MAPK3,POLR2J,EP300,GNB1,SHC1 (includes EG:20416),POLR2A,AKT1,PLCE1,MAP2K2,PIK3R2,ADCY9,GRM8,ITPR2,CREBBP,ADCY3,GNG10,GNAI2,GRM5,PRKCI,POLR2E,ITPR3,GNB2,PRKAG2,PIK3CD,ELK1,CAMK2G |
| Induction of Apoptosis by HIV1 | 2.38E00 | 1.97E-01 | TP53 (includes EG:22059),TNFRSF1A,BAX,MAP3K5,NFKB2,BCL2,IKBKB,BCL2L1,IKBKG,SLC25A6,NFKBIA,BBC3,FASLG |
| Aryl Hydrocarbon Receptor Signaling | 2.33E00 | 1.51E-01 | TP53 (includes EG:22059),MAPK3,SLC35A2,MDM2,NFKB2,BAX,CCND1,EP300,ALDH3B2,HSP90B1,ALDH1A1,JUN,NCOA2,HSP90AB1,TGFB1 (includes EG:21803),RARB,NFIB,DHFR,NFE2L2,ALDH5A1,CDK2,FASLG,HSPB1,ALDH7A1 |
| IL-15 Signaling | 2.32E00 | 1.94E-01 | STAT6,IL2RG,MAPK3,PIK3R1,NFKB2,BCL2,SHC1 (includes EG:20416),BCL2L1,AKT1,MAP2K2,PIK3CD,PIK3R2,STAT5B |
| Coagulation System | 2.31E00 | 2.37E-01 | KNG1,SERPINC1,PROS1,F5,PLAUR,F13B,FGB (includes EG:110135),TFPI,FGG |
| 4-1BB Signaling in T Lymphocytes | 2.3E00 | 2.35E-01 | IKBKB,IKBKG,JUN,NFKBIA,MAP2K2,MAPK3,MAP3K5,NFKB2 |
| TNFR1 Signaling | 2.29E00 | 2.08E-01 | TANK,CASP6,IKBKB,PAK4,IKBKG,JUN,PAK2,NFKBIA,TNFRSF1A,CASP2,NFKB2 |
| Gα12/13 Signaling | 2.29E00 | 1.64E-01 | MYL6,MAPK3,PIK3R1,MEF2A,ARHGEF1,NFKB2,MAP3K5,MYL9,IKBKB,MYL12A,IKBKG,JUN,NFKBIA,AKT1,MAP2K2,MEF2D,PIK3CD,PIK3R2,MYL10,ELK1,CTNNB1 |
| TGF-β Signaling | 2.27E00 | 1.8E-01 | INHA,MAPK3,CREBBP,SMAD6,BMPR2,PITX2,INHBB,BCL2,EP300,JUN,MAP2K2,TGFB1 (includes EG:21803),RUNX2,MAP4K1,TFE3,ACVR2A |
| Insulin Receptor Signaling | 2.26E00 | 1.64E-01 | SOCS3,FOXO4,EIF2B4,BAD,TRIP10,PIK3R1,MAPK3,CRKL,GSK3A,PPP1R11,CRK,VAMP2,PTPRF,EIF4E,SHC1 (includes EG:20416),AKT1,PRKCI,MAP2K2,PTPN11,FOXO3,PRKAG2,PIK3CD,PIK3R2 |
| Prolactin Signaling | 2.26E00 | 1.88E-01 | SOCS1,SOCS3,MAPK3,PIK3R1,CREBBP,SOCS6,EP300,SHC1 (includes EG:20416),PRKCI,JUN,MAP2K2,PTPN11,PIK3CD,PIK3R2,STAT5B |
| HER-2 Signaling in Breast Cancer | 2.21E00 | 1.85E-01 | TP53 (includes EG:22059),ITGB1,BAD,PIK3R1,MDM2,MMP2,GSK3A,MAP3K5,CCND1,PRKCI,AKT1,PIK3CD,ITGB4,PIK3R2,EGFR |
| PDGF Signaling | 2.21E00 | 1.9E-01 | PDGFA,MAPK3,CRKL,PIK3R1,CRK,PDGFB,SHC1 (includes EG:20416),JUN,MAP2K2,CSNK2A1,PDGFRA,PIK3CD,PIK3R2,ELK1,PDGFRB |
| Activation of IRF by Cytosolic Pattern Recognition Receptors | 2.19E00 | 1.81E-01 | PPIB,CREBBP,NFKB2,IRF3,TANK,IKBKB,IRF7,IKBKG,JUN,NFKBIA,STAT2,PIN1,IFNAR1 |
| ERK5 Signaling | 2.19E00 | 2.03E-01 | IL6ST,LIF,BAD,YWHAE,YWHAB,MEF2A,NGF,AKT1,PTPN11,MEF2D,FOXO3,FOSL1,EGFR |
| Rac Signaling | 2.19E00 | 1.54E-01 | ITGB1,PAK4,MAP3K11,PAK2,CFL1,MAPK3,PIK3R1,ITGA5,NFKB2,IQGAP2,PRKCI,JUN,ARFIP2,MAP2K2,PIP5K1C,CYBB,PIK3CD,PIK3R2,ELK1 |
| EGF Signaling | 2.15E00 | 2.12E-01 | SHC1 (includes EG:20416),JUN,ITPR2,MAPK3,PIK3R1,ITPR3,CSNK2A1,PIK3CD,PIK3R2,ELK1,EGFR |
| Non-Small Cell Lung Cancer Signaling | 2.14E00 | 1.77E-01 | TP53 (includes EG:22059),BAD,ITPR2,MAPK3,PIK3R1,CCND1,AKT1,MAP2K2,RARB,FOXO3,ITPR3,PIK3CD,PIK3R2,EGFR |
| Butanoate Metabolism | 2.12E00 | 1.23E-01 | ACAT2,RDH11,SUCLG2,SDHC,ELOVL6,AADAC,ALDH1A1,ACAT1,SDHD,DBT,EHHADH,PDHB,HMGCS1,ALDH5A1,HADH,ALDH7A1 |
| Aldosterone Signaling in Epithelial Cells | 2.11E00 | 1.53E-01 | CRYAB,HSPA1A/HSPA1B,PIK3R1,MAPK3,DNAJC3,HSPA4,HSP90B1,PLCE1,HSP90AB1,MAP2K2,DNAJC8,PIK3R2,DNAJC22,HSPB6,DNAJC9,ITPR2,HSPA9,DNAJB9,DNAJB14,PRKCI,DUSP1,PIP5K1C,ITPR3,PIK3CD,DNAJB5,HSPB1 |
| Melanoma Signaling | 2.1E00 | 2.17E-01 | TP53 (includes EG:22059),AKT1,BAD,MAP2K2,MAPK3,PIK3R1,MDM2,PIK3CD,PIK3R2,CCND1 |
| FLT3 Signaling in Hematopoietic Progenitor Cells | 2.09E00 | 1.89E-01 | STAT6,BAD,MAPK3,PIK3R1,EIF4E,SHC1 (includes EG:20416),AKT1,MAP2K2,PTPN11,PIK3CD,STAT2,PIK3R2,ELK1,STAT5B |
| T Cell Receptor Signaling | 2.07E00 | 1.65E-01 | CALM1 (includes others),MAPK3,CD4,PIK3R1,NFATC1,NFKB2,PPP3CC,SHB,IKBKB,IKBKG,NFKBIA,NFAT5,JUN,MAP2K2,PPP3R1,PIK3CD,PIK3R2,ELK1 |
| April Mediated Signaling | 2.06E00 | 2.09E-01 | IKBKB,IKBKG,NFAT5,JUN,NFKBIA,TNFSF13,NFATC1,NFKB2,ELK1 |
| 14-3-3-mediated Signaling | 2.05E00 | 1.67E-01 | YWHAE,BAD,TNFRSF1A,YWHAB,TUBB2C,MAPK3,PIK3R1,GSK3A,BAX,MAP3K5,TUBB,TUBB2B,PLCE1,JUN,AKT1,PRKCI,MAP2K2,PIK3CD,PIK3R2,ELK1 |
| Renin-Angiotensin Signaling | 2.05E00 | 1.59E-01 | ADCY9,PTPN6,PAK4,PAK2,ITPR2,MAPK3,PIK3R1,ADCY3,NFKB2,SHC1 (includes EG:20416),PRKCI,JUN,MAP2K2,CCL2,ITPR3,PRKAG2,PIK3CD,PIK3R2,ELK1,ACE |
| LPS-stimulated MAPK Signaling | 2.03E00 | 1.71E-01 | MAPK3,PIK3R1,MAP3K5,NFKB2,IKBKB,IKBKG,PRKCI,JUN,NFKBIA,MAP2K2,PIK3CD,LBP,PIK3R2,ELK1 |
| Thyroid Cancer Signaling | 2.03E00 | 2.17E-01 | TP53 (includes EG:22059),SHC1 (includes EG:20416),TCF4,MAP2K2,MAPK3,NGF,TCF3,CTNNB1,CCND1,TCF7L2 |
| DNA Methylation and Transcriptional Repression Signaling | 2.02E00 | 2.61E-01 | MECP2,DNMT3A,MTA1,CHD4,SAP18,RBBP4 |
| Myc Mediated Apoptosis Signaling | 2.01E00 | 1.97E-01 | TP53 (includes EG:22059),SHC1 (includes EG:20416),AKT1,BAD,YWHAE,YWHAB,PIK3R1,PIK3CD,PIK3R2,BAX,FASLG,BCL2 |
| **Canonical Pathways** | **-log(p-value)** | **Ratio** | **Molecules** |
| PI3K/AKT Signaling | 8.46E00 | 2.5E-01 | BAD,PIK3R1,MAPK3,LIMS1,ILK,GSK3A,MAP3K5,CCND1,EIF4E,BCL2,SHC1 (includes EG:20416),IKBKB,HSP90B1,IKBKG,NFKBIA,AKT1,HSP90AB1,MAP2K2,FOXO3,PIK3R2,CTNNB1,MCL1,TP53 (includes EG:22059),ITGB1,CDC37,RHEB,YWHAE,YWHAB,ITGA5,MDM2,NFKB2,BCL2L1,PPP2R1A,PPP2R4,PIK3CD |
| Molecular Mechanisms of Cancer | 7.93E00 | 1.79E-01 | BAD,MAPK3,PIK3R1,NCSTN,CDKN2C,GSK3A,ARHGEF1,RBL1,CCND1,RHOG,RHOB,MAP2K2,TGFB1 (includes EG:21803),HIPK2,FASLG,ADCY9,TP53 (includes EG:22059),CREBBP,ARHGEF17,NFKB2,AURKA,TCF3,RAP1A,APC,BCL2L1,PTPN11,PIK3CD,FZD5,NOTCH1,CDK2,CAMK2G,TCF4,BMPR2,PSENEN,CRK,FZD1,MAP3K5,E2F3,BCL2,EP300,CASP6,SHC1 (includes EG:20416),NFKBIA,NLK,JUN,AKT1,RHOT1,BBC3,ARHGEF2,PIK3R2,CTNNB1,BMP1,PAK4,PAK2,ADCY3,SMAD6,MDM2,BAX,APH1A,NBN,GNAI2,ARHGEF10,PRKCI,FZD4,NF1 (includes EG:18015),PRKAG2,ELK1,CTNND1 |
| PTEN Signaling | 6.98E00 | 2.42E-01 | FOXO4,BAD,PIK3R1,MAPK3,ILK,BMPR2,GSK3A,CCND1,BCL2,SHC1 (includes EG:20416),IKBKB,IKBKG,AKT1,MAP2K2,FOXO3,CSNK2A1,PDGFRA,PIK3R2,FASLG,PDGFRB,EGFR,ITGB1,FGFR1,ITGA5,FGFR2,NFKB2,IGF2R,DDR1,BCL2L1,PIK3CD |
| Apoptosis Signaling | 6.78E00 | 2.71E-01 | BAD,MAPK3,MAP4K4,MAP3K5,BCL2,ACIN1,CASP6,IKBKB,IKBKG,NFKBIA,MAP2K2,FASLG,MCL1,AIFM1,TP53 (includes EG:22059),TNFRSF1A,LMNA,BAX,NFKB2,CDK1,BCL2L1,CAPNS1,CASP2,CAPN1,CAPN2,SPTAN1 |
| Chronic Myeloid Leukemia Signaling | 5.71E00 | 2.38E-01 | BAD,PIK3R1,MAPK3,HDAC10,CRK,E2F3,RBL1,CCND1,CTBP1,IKBKB,IKBKG,AKT1,MAP2K2,TGFB1 (includes EG:21803),HDAC7,CTBP2,PIK3R2,STAT5B,TP53 (includes EG:22059),CRKL,MDM2,NFKB2,BCL2L1,PTPN11,PIK3CD |
| Integrin Signaling | 5.64E00 | 1.95E-01 | MPRIP,ARHGAP26,MAP3K11,PIK3R1,MAPK3,LIMS1,ILK,CRK,TLN1,MYLK,SHC1 (includes EG:20416),ARF6,AKT1,RHOG,RHOB,MAP2K2,RHOT1,PIK3R2,ITGB4,TSPAN4,ACTN1,ITGB1,PAK4,PAK2,CRKL,ACTB,ITGA5,RAP1A,ARHGAP5,MYL9,MYL12A,TLN2,CAPNS1,ARF3,WAS,CAPN1,ZYX,CAPN2,PIK3CD,ACTN4,ITGA7 |
| Androgen Signaling | 5.49E00 | 1.94E-01 | POLR2D,CALM1 (includes others),MAPK3,POLR2J,CCND1,EP300,GNB1,HSPA4,SHC1 (includes EG:20416),TGFB1I1,POLR2A,JUN,AR,NCOA2,NCOA4,CALR,CREBBP,NFKB2,GNG10,GNAI2,KAT2B,PRKCI,POLR2E,GTF2E1,NCOA1,GNB2,PRKAG2,GTF2H1 |
| Acute Phase Response Signaling | 5.38E00 | 2.02E-01 | IL6ST,SOCS3,SOCS1,TCF4,saa3p,PIK3R1,APOA2,MAPK3,SOCS6,AMBP,CP,MAP3K5,FGG,IKBKB,SHC1 (includes EG:20416),IKBKG,IL36G,NFKBIA,JUN,AKT1,MAP2K2,APCS,FGB (includes EG:110135),LBP,PIK3R2,TTR,TNFRSF1A,AHSG,IL36A,NFKB2,TCF3,ALB,TF,PTPN11,PIK3CD,ELK1 |
| p53 Signaling | 5.26E00 | 2.5E-01 | TP53 (includes EG:22059),GADD45B,PIK3R1,CSNK1D,MDM2,BAX,CCND1,TP53I3,EP300,BCL2,CASP6,BCL2L1,KAT2B,JUN,AKT1,BBC3,STAG1,ADCK3,PIK3CD,PIK3R2,PML,HIPK2,CTNNB1,CDK2 |
| PPAR Signaling | 5E00 | 2.24E-01 | TNFRSF1A,PDGFA,MAPK3,CREBBP,IL36A,MAP4K4,NFKB2,PDGFB,EP300,SHC1 (includes EG:20416),IKBKB,IL36G,IKBKG,HSP90B1,JUN,NFKBIA,MAP2K2,HSP90AB1,PDGFRA,NCOA1,IL1RAPL1,STAT5B,CITED2,PDGFRB |
| Renal Cell Carcinoma Signaling | 5E00 | 2.7E-01 | RBX1,PAK4,PAK2,PIK3R1,MAPK3,CREBBP,CRK,RAP1A,PDGFB,EP300,VEGFA,AKT1,JUN,MAP2K2,PTPN11,CUL2,TGFB1 (includes EG:21803),PIK3CD,PIK3R2,FH |
| Wnt/β-catenin Signaling | 4.85E00 | 2.01E-01 | TCF4,CSNK1G1,SOX15,CSNK1A1,ILK,GSK3A,FZD1,CCND1,EP300,JUN,AKT1,NLK,TGFB1 (includes EG:21803),RARB,CSNK2A1,SFRP5,MAP4K1,CTNNB1,TP53 (includes EG:22059),SOX4,APPL2,CSNK1G3,CREBBP,CSNK1D,MDM2,TCF3,APC,PPP2R1A,FZD4,PPP2R4,NR5A2,FZD5,PIN1,ACVR2A,TCF7L2 |
| Glucocorticoid Receptor Signaling | 4.72E00 | 1.63E-01 | TAF11 (includes EG:309638),POLR2D,HSPA1A/HSPA1B,MAPK3,PIK3R1,POLR2J,TAF13 (includes EG:310784),FGG,BCL2,EP300,HSPA4,SHC1 (includes EG:20416),IKBKB,IKBKG,HSP90B1,NFKBIA,AR,POLR2A,NFAT5,AKT1,JUN,HSP90AB1,NCOA2,CCL2,MAP2K2,TGFB1 (includes EG:21803),BAG1,PPP3R1,FOXO3,PIK3R2,STAT5B,TAF6,HSPA9,CREBBP,NFATC1,PPP3CC,KAT2B,BCL2L1,SUMO1,DUSP1,SMARCA2,POLR2E,GTF2E1,NCOA1,PRKAG2,GTF2H1,PIK3CD,ELK1 |
| PAK Signaling | 4.4E00 | 2.06E-01 | ITGB1,PAK4,PAK2,ARHGAP10,MYL6,CFL1,PDGFA,PIK3R1,MAPK3,PAK1IP1,ITGA5,PDGFB,MYLK,MYL9,SHC1 (includes EG:20416),MYL12A,MAP2K2,PDGFRA,PIK3CD,PIK3R2,MYL10,PDGFRB |
| Prostate Cancer Signaling | 4.37E00 | 2.16E-01 | TP53 (includes EG:22059),BAD,SRD5A1,MAPK3,PIK3R1,CREBBP,MDM2,NFKB2,CCND1,BCL2,HSP90B1,AKT1,NFKBIA,AR,MAP2K2,HSP90AB1,NKX3-1,PIK3CD,PIK3R2,CTNNB1,CDK2 |
| Role of Osteoblasts, Osteoclasts and Chondrocytes in Rheumatoid Arthritis | 4.35E00 | 1.71E-01 | TCF4,CALM1 (includes others),BAD,PIK3R1,MAPK3,CSNK1A1,BMPR2,MAP3K5,FZD1,BCL2,IKBKB,IL36G,IKBKG,NFKBIA,NFAT5,AKT1,JUN,RUNX2,TGFB1 (includes EG:21803),PPP3R1,SFRP5,PIK3R2,CTNNB1,BMP1,ITGB1,TNFRSF1A,IL36A,SMAD6,ITGA5,NFATC1,PPP3CC,TCF3,CSF1R,APC,COL1A1,FZD4,CSF1 (includes EG:12977),FZD5,PIK3CD,IL1RAPL1,TCF7L2 |
| Phospholipase C Signaling | 4.3E00 | 1.65E-01 | MPRIP,TRD@,CALM1 (includes others),MYL6,MAPK3,HDAC10,ARHGEF1,CD79A,EP300,GNB1,SHC1 (includes EG:20416),NFAT5,RHOG,PLCE1,RHOB,MAP2K2,RHOT1,PPP3R1,GPLD1,HDAC7,ARHGEF2,MYL10,ADCY9,ITGB1,PLD3,ITPR2,ADCY3,CREBBP,MEF2A,ITGA5,NFATC1,ARHGEF17,PPP3CC,NFKB2,RAP1A,GNG10,MYL9,MYL12A,PRKCI,ARHGEF10,MEF2D,ITPR3,GNB2 |
| Hepatic Fibrosis / Hepatic Stellate Cell Activation | 4.23E00 | 1.97E-01 | IGFBP4,MYH10,MYL6,PDGFA,BCL2,VEGFA,CCL2,TGFB1 (includes EG:21803),TIMP1,PDGFRA,LBP,FASLG,EGFR,PDGFRB,TNFRSF1A,FGFR1,FGFR2,MMP2,BAX,NFKB2,IFNAR2,PDGFB,FGF1,MYL9,COL1A1,CSF1 (includes EG:12977),MYH9,IL1RAPL1,IFNAR1 |
| Estrogen Receptor Signaling | 4.18E00 | 1.99E-01 | TAF11 (includes EG:309638),POLR2D,MAPK3,POLR2J,H3F3A/H3F3B,TAF13 (includes EG:310784),EP300,CTBP1,SPEN,SHC1 (includes EG:20416),CDK8,POLR2A,MAP2K2,NCOA2,MED15,CTBP2,TAF6,CREBBP,MED6 (includes EG:10001),KAT2B,POLR2E,MED13L,GTF2E1,NCOA1,GTF2H1,MED4 (includes EG:29079),CARM1 |
| Erythropoietin Signaling | 4.08E00 | 2.31E-01 | EPO,SOCS1,SOCS3,PTPN6,EPOR,MAPK3,PIK3R1,NFKB2,SHC1 (includes EG:20416),NFKBIA,PRKCI,AKT1,JUN,MAP2K2,PIK3CD,PIK3R2,STAT5B,ELK1 |
| Huntington's Disease Signaling | 4.01E00 | 1.68E-01 | POLR2D,HSPA1A/HSPA1B,REST,MAPK3,PIK3R1,POLR2J,HDAC10,NGF,EP300,GNB1,HSPA4,CASP6,SHC1 (includes EG:20416),ARFIP2,POLR2A,AKT1,JUN,CPLX2,HDAC7,VAMP3,PIK3R2,NAPA,EGFR,TP53 (includes EG:22059),HSPA9,CREBBP,BAX,GNG10,GRM5,RCOR1,BCL2L1,PRKCI,CAPNS1,POLR2E,CASP2,CAPN1,GNB2,STX16,CAPN2,PIK3CD |
| Glioblastoma Multiforme Signaling | 4E00 | 1.83E-01 | PDGFA,PIK3R1,MAPK3,FZD1,E2F3,CCND1,SHC1 (includes EG:20416),RHOG,AKT1,PLCE1,MAP2K2,RHOB,RHOT1,PDGFRA,PIK3R2,CTNNB1,EGFR,PDGFRB,TP53 (includes EG:22059),ITPR2,MDM2,TCF3,PDGFB,APC,FZD4,NF1 (includes EG:18015),ITPR3,FZD5,PIK3CD,CDK2 |
| NRF2-mediated Oxidative Stress Response | 3.99E00 | 1.77E-01 | AKR7A2,RBX1,PPIB,PIK3R1,MAPK3,DNAJC3,MAP3K5,SOD3,EP300,JUN,AKT1,MAP2K2,DNAJC8,PMF1,FOSL1,DNAJA2,JUND,TXN (includes EG:116484),PIK3R2,NFE2L2,DNAJC9,ACTB,CREBBP,SLC35A2,JUNB,DNAJB9,DNAJB14,PRKCI,STIP1,CAT,PIK3CD,PTPLAD1,DNAJB5,EPHX1 |
| FAK Signaling | 3.99E00 | 2.06E-01 | ITGB1,PAK4,PAK2,ARHGAP26,ACTB,PIK3R1,MAPK3,ITGA5,CRK,TLN1,CAPNS1,TLN2,AKT1,MAP2K2,WAS,CAPN1,CAPN2,PIK3CD,PIK3R2,TNS1,EGFR |
| Reelin Signaling in Neurons | 3.85E00 | 2.32E-01 | ITGB1,MAP3K11,PIK3R1,CRKL,MAPK8IP2,ITGA5,ARHGEF1,RELN,MAPK8IP3,FRK,APP,APBB1,YES1,AKT1,ARHGEF10,MAP4K1,ARHGEF2,PIK3CD,PIK3R2 |
| Hereditary Breast Cancer Signaling | 3.83E00 | 1.94E-01 | POLR2D,GADD45B,PIK3R1,POLR2J,HDAC10,DDB2,CCND1,RAD50,EP300,POLR2A,AKT1,HDAC7,RFC2,PIK3R2,TP53 (includes EG:22059),CREBBP,RFC5,CDK1,NBN,PALB2,SMARCA2,POLR2E,H2AFX,PIK3CD,RFC3 |
| JAK/Stat Signaling | 3.8E00 | 2.5E-01 | STAT6,SOCS1,SOCS3,PTPN6,PIAS2,MAPK3,PIK3R1,SOCS6,SHC1 (includes EG:20416),AKT1,PTPN11,MAP2K2,STAT2,PIK3CD,PIK3R2,STAT5B |
| Neuregulin Signaling | 3.77E00 | 2.06E-01 | ITGB1,BAD,PIK3R1,MAPK3,CRKL,ITGA5,CRK,SHC1 (includes EG:20416),ERBB2IP,HSP90B1,PRKCI,AKT1,PICK1,MAP2K2,PTPN11,HSP90AB1,PIK3R2,STAT5B,ELK1,EGFR,MATK |
| Clathrin-mediated Endocytosis Signaling | 3.75E00 | 1.8E-01 | RAB4A,PDGFA,PIK3R1,GAK,VEGFA,CD2AP,ARF6,AP1G2,RAB11B,PPP3R1,CSNK2A1,PIK3R2,SH3GLB2,ITGB4,HGS,ITGB1,MYO6,AP2M1,EPN1,ACTB,ITGA5,MDM2,PPP3CC,PDGFB,FGF1,FGF21,ARRB2,TF,PIP5K1C,CLTA,PIK3CD |
| Glioma Signaling | 3.75E00 | 1.96E-01 | TP53 (includes EG:22059),CALM1 (includes others),PDGFA,PIK3R1,MAPK3,CDKN2C,MDM2,E2F3,RBL1,CCND1,IGF2R,PDGFB,SHC1 (includes EG:20416),AKT1,PRKCI,MAP2K2,PDGFRA,PIK3CD,PIK3R2,EGFR,PDGFRB,CAMK2G |
| IL-6 Signaling | 3.7E00 | 2.1E-01 | IL6ST,SOCS1,TNFRSF1A,MAPK3,IL36A,MAP4K4,NFKB2,COL1A1,SHC1 (includes EG:20416),IKBKB,IKBKG,IL36G,JUN,NFKBIA,MAP2K2,PTPN11,CSNK2A1,IL1RAPL1,LBP,ELK1,HSPB1 |
| Role of NFAT in Regulation of the Immune Response | 3.68E00 | 1.65E-01 | CSNK1G1,CALM1 (includes others),TRD@,PIK3R1,CD4,MAPK3,CSNK1A1,GSK3A,CD79A,CABIN1,GNB1,IKBKB,IKBKG,NFKBIA,JUN,AKT1,NFAT5,MAP2K2,PPP3R1,PIK3R2,ITPR2,CSNK1G3,CSNK1D,MEF2A,NFATC1,PPP3CC,NFKB2,GNG10,GNAI2,MEF2D,ITPR3,GNB2,PIK3CD |
| IGF-1 Signaling | 3.68E00 | 2.06E-01 | IGFBP4,SOCS1,SOCS3,YWHAE,BAD,YWHAB,MAPK3,PIK3R1,SOCS6,SHC1 (includes EG:20416),PRKCI,AKT1,JUN,MAP2K2,PTPN11,FOXO3,CSNK2A1,PRKAG2,PIK3CD,PIK3R2,ELK1,CYR61 |
| SAPK/JNK Signaling | 3.63E00 | 2.06E-01 | TP53 (includes EG:22059),MAP3K11,DUSP8,TRD@,PIK3R1,CRKL,MAPK8IP2,MAP4K4,CRK,NFATC1,MAP3K5,MAPK8IP3,GNB1,SHC1 (includes EG:20416),MINK1,JUN,DUSP10,MAP4K1,PIK3CD,PIK3R2,ELK1 |
| Germ Cell-Sertoli Cell Junction Signaling | 3.49E00 | 1.8E-01 | MAP3K11,PIK3R1,MAPK3,ILK,MAP3K5,TUBB,TUBB2B,AGGF1,AKT1,RHOG,MAP2K2,RHOB,RHOT1,TGFB1 (includes EG:21803),MTMR2,PIK3R2,JUP,CTNNB1,ACTN1,ITGB1,EPN1,PAK4,PAK2,TUBB2C,TNFRSF1A,ACTB,ZYX,PIK3CD,ACTN4,CTNND1 |
| IL-3 Signaling | 3.48E00 | 2.3E-01 | STAT6,PTPN6,BAD,MAPK3,CRKL,PIK3R1,PPP3CC,SHC1 (includes EG:20416),PRKCI,AKT1,JUN,MAP2K2,PPP3R1,PIK3CD,PIK3R2,STAT5B,ELK1 |
| RAR Activation | 3.43E00 | 1.71E-01 | PIK3R1,ADH1C (includes EG:11522),MAP3K5,EP300,VEGFA,ALDH1A1,AKT1,JUN,TGFB1 (includes EG:21803),RARB,CSNK2A1,SORBS3,PIK3R2,STAT5B,CITED2,ADCY9,RDH14,RDH11,CREBBP,ADCY3,SMAD6,NFKB2,KAT2B,PRKCI,SMARCA2,DUSP1,NCOA1,PRKAG2,GTF2H1,PIK3CD,PML,CARM1 |
| B Cell Receptor Signaling | 3.36E00 | 1.79E-01 | MAP3K11,BAD,CALM1 (includes others),PIK3R1,MAPK3,GSK3A,MAP3K5,CD79A,SHC1 (includes EG:20416),IKBKB,IKBKG,AKT1,JUN,NFAT5,NFKBIA,MAP2K2,PPP3R1,CD22,PIK3R2,PTPN6,NFATC1,PPP3CC,NFKB2,BCL2L1,PTPN11,PIK3CD,ELK1,CAMK2G |
| Hypoxia Signaling in the Cardiovascular System | 3.3E00 | 2.35E-01 | TP53 (includes EG:22059),EPO,CSNK1D,MDM2,UBE2D1,EP300,VEGFA,HSP90B1,AKT1,JUN,NFKBIA,SUMO1,HSP90AB1,UBE2B,UBE2G1,UBE2J1 |
| IL-2 Signaling | 3.25E00 | 2.41E-01 | SOCS1,IL2RG,MAPK3,PIK3R1,SHC1 (includes EG:20416),AKT1,JUN,MAP2K2,PTPN11,CSNK2A1,PIK3CD,PIK3R2,ELK1,STAT5B |
| Pancreatic Adenocarcinoma Signaling | 3.25E00 | 1.85E-01 | TP53 (includes EG:22059),PLD3,BAD,PIK3R1,MAPK3,MDM2,NFKB2,E2F3,CCND1,BCL2,VEGFA,BCL2L1,AKT1,MAP2K2,TGFB1 (includes EG:21803),GPLD1,PIK3CD,PIK3R2,ELK1,NOTCH1,CDK2,EGFR |
| Thrombin Signaling | 3.23E00 | 1.64E-01 | MPRIP,MYL6,PIK3R1,MAPK3,ARHGEF1,MYLK,GNB1,SHC1 (includes EG:20416),IKBKB,RHOG,AKT1,PLCE1,RHOB,MAP2K2,RHOT1,ARHGEF2,PIK3R2,MYL10,EGFR,ADCY9,ITPR2,ADCY3,NFKB2,GNG10,MYL9,GNAI2,MYL12A,ARHGEF10,PRKCI,ITPR3,GNB2,PIK3CD,ELK1,CAMK2G |
| Extrinsic Prothrombin Activation Pathway | 3.2E00 | 3.5E-01 | SERPINC1,PROS1,F5,F13B,FGB (includes EG:110135),TFPI,FGG |
| Protein Kinase A Signaling | 3.17E00 | 1.49E-01 | MYH10,BAD,MYL6,MAPK3,GSK3A,MYLK,GNB1,PLCE1,MAP2K2,TGFB1 (includes EG:21803),PPP3R1,MYL10,ADCY9,YWHAE,YWHAB,ITPR2,PDE9A,CREBBP,PPP1R11,NFKB2,PPP3CC,TCF3,RAP1A,GNG10,MYL9,AKAP13,MYL12A,ITPR3,GNB2,CAMK2G,ANAPC2,TCF4,CALM1 (includes others),H3F3A/H3F3B,AKAP7,NFAT5,NFKBIA,FLNA,CTNNB1,ADCY3,PYGL,NFATC1,GNAI2,PRKCI,PRKAG2,ADD1,ELK1,TCF7L2,CDC27 |
| GM-CSF Signaling | 3.11E00 | 2.24E-01 | MAPK3,PIK3R1,PPP3CC,CCND1,SHC1 (includes EG:20416),BCL2L1,AKT1,MAP2K2,PTPN11,PPP3R1,PIK3CD,PIK3R2,ELK1,STAT5B,CAMK2G |
| Role of CHK Proteins in Cell Cycle Checkpoint Control | 3.1E00 | 2.86E-01 | TP53 (includes EG:22059),RAD17 (includes EG:19356),RFC2,RFC5,E2F3,CDK1,RAD50,CDK2,RFC3,NBN |
| Role of Tissue Factor in Cancer | 3.08E00 | 1.93E-01 | TP53 (includes EG:22059),ITGB1,CFL1,PIK3R1,MAPK3,PLAUR,FRK,EIF4E,FGG,VEGFA,BCL2L1,YES1,ARRB2,AKT1,PTPN11,CSF1 (includes EG:12977),FGB (includes EG:110135),PIK3CD,PIK3R2,STAT5B,CYR61,EGFR |
| Actin Cytoskeleton Signaling | 3.05E00 | 1.51E-01 | MYH10,PFN1,MPRIP,MYL6,PDGFA,PIK3R1,MAPK3,ARHGEF1,CRK,MYLK,SHC1 (includes EG:20416),IQGAP2,MAP2K2,LBP,PIK3R2,MYL10,ACTN1,MATK,ITGB1,PAK4,PAK2,CFL1,ACTB,CRKL,ITGA5,PDGFB,APC,FGF1,MYL9,FGF21,MYL12A,PIP5K1C,WAS,MYH9,PIK3CD,ACTN4 |
| Protein Ubiquitination Pathway | 3.04E00 | 1.53E-01 | PSMB3,B2M,ANAPC2,USP24,RBX1,CRYAB,USP5,HSPA1A/HSPA1B,UBE3B,UBR2 (includes EG:224826),DNAJC3,HSPA4,HSP90B1,HSP90AB1,UBE2B,BAG1,PSMC6,DNAJC8,UCHL5,NEDD4L,PSMA2,DNAJC22,UBE2J1,AMFR,HSPB6,DNAJC9,USP15,HSPA9,MDM2,DNAJB9,USP33,DNAJB14,UBE2D1,FZR1,CUL2,UBE2G1,PSMA4,USP46,UBA1,USP25,DNAJB5,HSPB1 |
| Polyamine Regulation in Colon Cancer | 3.02E00 | 2.76E-01 | TCF4,AZIN1,PSMF1,SAT1,PSME4,CTNNB1,PSME3,APC |
| ILK Signaling | 3.01E00 | 1.66E-01 | MYH10,MYL6,PIK3R1,MAPK3,ILK,GSK3A,CCND1,VEGFA,TGFB1I1,RHOG,AKT1,JUN,RHOB,FLNA,RHOT1,PIK3R2,ITGB4,CTNNB1,ACTN1,ITGB1,CFL1,TNFRSF1A,ACTB,FERMT2,SNAI1,NFKB2,MYL9,PPP2R1A,PPP2R4,MYH9,PIK3CD,ACTN4 |
| ATM Signaling | 2.98E00 | 2.41E-01 | TP53 (includes EG:22059),SMC3,GADD45B,MDM2,CDK1,RAD50,SMC1A,NBN,JUN,NFKBIA,SMC2,H2AFX,CDK2 |
| Amyloid Processing | 2.98E00 | 2.32E-01 | MAPK3,CSNK1D,CSNK1A1,NCSTN,PSENEN,APP,APH1A,AKT1,CAPNS1,CAPN1,CSNK2A1,PRKAG2,CAPN2 |
| Role of NFAT in Cardiac Hypertrophy | 2.97E00 | 1.54E-01 | IL6ST,LIF,CALM1 (includes others),PIK3R1,MAPK3,CSNK1A1,HDAC10,EP300,CABIN1,GNB1,SHC1 (includes EG:20416),AKT1,PLCE1,MAP2K2,TGFB1 (includes EG:21803),PPP3R1,HDAC7,PIK3R2,ADCY9,ITPR2,ADCY3,MEF2A,PPP3CC,GNG10,GNAI2,PRKCI,MEF2D,ITPR3,GNB2,PRKAG2,PIK3CD,CAMK2G |
| TR/RXR Activation | 2.96E00 | 1.98E-01 | AKR1C1/AKR1C2,GPS2,PIK3R1,BCL3,MDM2,ME1,EP300,KLF9,SLC16A3,UCP3,AKT1,NCOA2,SREBF2,DIO1,NCOA1,NCOA4,PIK3CD,PIK3R2,SYT12 |
| Colorectal Cancer Metastasis Signaling | 2.94E00 | 1.51E-01 | IL6ST,TCF4,BAD,PIK3R1,MAPK3,FZD1,CCND1,VEGFA,GNB1,RHOG,AKT1,JUN,RHOB,MMP25,MAP2K2,RHOT1,TGFB1 (includes EG:21803),PIK3R2,CTNNB1,EGFR,MMP19,TP53 (includes EG:22059),ADCY9,TNFRSF1A,ADRBK2,ADCY3,MMP2,BAX,NFKB2,TCF3,APC,GNG10,BCL2L1,FZD4,GNB2,PRKAG2,FZD5,PIK3CD,TCF7L2 |
| Death Receptor Signaling | 2.92E00 | 2.15E-01 | TNFRSF1A,MAP4K4,MAP3K5,NFKB2,BCL2,TANK,CASP6,IKBKB,IKBKG,NFKBIA,TNFSF12,CASP2,FASLG,HSPB1 |
| VEGF Signaling | 2.9E00 | 1.92E-01 | EIF2S3,EIF2B4,YWHAE,BAD,ACTB,MAPK3,PIK3R1,EIF1,BCL2,VEGFA,SHC1 (includes EG:20416),BCL2L1,AKT1,MAP2K2,FOXO3,PIK3CD,PIK3R2,ACTN4,ACTN1 |
| Role of JAK2 in Hormone-like Cytokine Signaling | 2.88E00 | 2.7E-01 | EPO,SHC1 (includes EG:20416),SOCS1,SOCS3,PTPN6,PTPN11,EPOR,SOCS6,STAT5B,SIRPA |
| fMLP Signaling in Neutrophils | 2.87E00 | 1.72E-01 | CALM1 (includes others),ITPR2,MAPK3,PIK3R1,NFATC1,NFKB2,PPP3CC,GNG10,GNB1,GNAI2,NFKBIA,NFAT5,PRKCI,MAP2K2,WAS,PPP3R1,ITPR3,GNB2,CYBB,PIK3CD,PIK3R2,ELK1 |
| Angiopoietin Signaling | 2.82E00 | 2.03E-01 | PAK4,PAK2,GRB14,BAD,PIK3R1,CRK,NFKB2,IKBKB,IKBKG,NFKBIA,AKT1,PTPN11,PIK3CD,PIK3R2,STAT5B |
| Endometrial Cancer Signaling | 2.82E00 | 2.28E-01 | TP53 (includes EG:22059),BAD,MAPK3,PIK3R1,ILK,CCND1,AKT1,MAP2K2,FOXO3,PIK3CD,PIK3R2,ELK1,CTNNB1 |
| CXCR4 Signaling | 2.81E00 | 1.66E-01 | MYL6,PIK3R1,MAPK3,CD4,CRK,GNB1,RHOG,AKT1,JUN,MAP2K2,RHOB,RHOT1,PIK3R2,MYL10,ADCY9,PAK4,PAK2,ITPR2,ADCY3,GNG10,GNAI2,MYL9,MYL12A,PRKCI,ITPR3,GNB2,PIK3CD,ELK1 |
| Breast Cancer Regulation by Stathmin1 | 2.81E00 | 1.57E-01 | CALM1 (includes others),PIK3R1,MAPK3,ARHGEF1,E2F3,TUBB,TUBB2B,GNB1,SHC1 (includes EG:20416),MAP2K2,RB1CC1,ARHGEF2,PIK3R2,TP53 (includes EG:22059),ADCY9,TUBB2C,ITPR2,ADCY3,ARHGEF17,PPP1R11,CDK1,GNG10,GNAI2,PPP2R1A,ARHGEF10,PRKCI,PPP2R4,ITPR3,GNB2,PRKAG2,PIK3CD,CDK2,CAMK2G |
| Axonal Guidance Signaling | 2.8E00 | 1.32E-01 | GLI2,PFN1,MYL6,MAPK3,PIK3R1,TUBB,VEGFA,GNB1,MAP2K2,PPP3R1,PLXNB2,MYL10,CFL1,CRKL,ITGA5,PPP3CC,RAP1A,PDGFB,GNG10,MYL9,MYL12A,PTPN11,RTN4,GNB2,PIK3CD,FZD5,PDGFA,SEMA6A,CRK,FZD1,NGF,EIF4E,TUBB2B,EPHB6,SHC1 (includes EG:20416),AKT1,NFAT5,PLXNA1,SDC2,EFNB1,PIK3R2,SHANK2,BMP1,ITGB1,EPHB4,PAK4,PAK2,NRP2,ADAM2,TUBB2C,NFATC1,PLXND1,GNAI2,PRKCI,FZD4,WAS,PRKAG2 |
| RANK Signaling in Osteoclasts | 2.79E00 | 1.89E-01 | MAP3K11,CALM1 (includes others),MAPK3,PIK3R1,NFATC1,PPP3CC,MAP3K5,NFKB2,IKBKB,IKBKG,NFKBIA,AKT1,JUN,MAP2K2,PPP3R1,PIK3CD,PIK3R2,ELK1 |
| Macropinocytosis Signaling | 2.75E00 | 1.97E-01 | ITGB1,PDGFA,PIK3R1,ITGA5,NGF,CSF1R,PDGFB,ARF6,PRKCI,ABI1,CSF1 (includes EG:12977),PIK3CD,ITGB4,PIK3R2,ACTN4 |
| Role of Macrophages, Fibroblasts and Endothelial Cells in Rheumatoid Arthritis | 2.73E00 | 1.38E-01 | IL6ST,SOCS1,SOCS3,TCF4,CALM1 (includes others),PDGFA,MAPK3,PIK3R1,IL32,CSNK1A1,FZD1,CCND1,VEGFA,IKBKB,IKBKG,IL36G,C5AR1,NFKBIA,NLK,NFAT5,AKT1,JUN,PLCE1,CCL2,MAP2K2,TGFB1 (includes EG:21803),PPP3R1,SFRP5,PIK3R2,LTBR,CTNNB1,TNFRSF1A,IL36A,NFATC1,PPP3CC,TCF3,PDGFB,APC,PRKCI,FZD4,CSF1 (includes EG:12977),FZD5,PIK3CD,IL1RAPL1,TCF7L2,CAMK2G |
| iCOS-iCOSL Signaling in T Helper Cells | 2.7E00 | 1.72E-01 | IL2RG,BAD,CALM1 (includes others),TRD@,ITPR2,CD4,PIK3R1,NFATC1,NFKB2,PPP3CC,SHC1 (includes EG:20416),IKBKB,IKBKG,AKT1,NFAT5,NFKBIA,PPP3R1,ITPR3,PIK3CD,PIK3R2,CAMK2G |
| Intrinsic Prothrombin Activation Pathway | 2.7E00 | 2.65E-01 | COL1A1,KNG1,KLK1,SERPINC1,PROS1,F5,F13B,FGB (includes EG:110135),FGG |
| Lymphotoxin β Receptor Signaling | 2.66E00 | 2.13E-01 | MAPK3,PIK3R1,CREBBP,NFKB2,EP300,IKBKB,BCL2L1,IKBKG,AKT1,NFKBIA,PIK3CD,LTBR,PIK3R2 |
| HIF1α Signaling | 2.64E00 | 1.85E-01 | TP53 (includes EG:22059),EPO,EGLN2,RBX1,MAPK3,PIK3R1,CREBBP,MMP2,MDM2,EP300,P4HTM,VEGFA,JUN,AKT1,MMP25,CUL2,NCOA1,PIK3CD,PIK3R2,MMP19 |
| Acute Myeloid Leukemia Signaling | 2.61E00 | 1.95E-01 | TCF4,BAD,MAPK3,PIK3R1,NFKB2,TCF3,CCND1,CSF1R,AKT1,MAP2K2,PIK3CD,JUP,PIK3R2,PML,STAT5B,TCF7L2 |
| Lysine Degradation | 2.61E00 | 1.22E-01 | SETD8,AASDHPPT,ACAT2,RDH11,ELOVL6,EP300,KAT2B,ALDH1A1,SMYD3,EHMT2,ACAT1,DBT,EHHADH,SHMT2,PLOD3,HADH,ALDH7A1 |
| PPARα/RXRα Activation | 2.61E00 | 1.56E-01 | CYP2C9,MAPK3,APOA2,MAP4K4,ADIPOR1,ABCA1,EP300,SHC1 (includes EG:20416),IKBKB,IKBKG,HSP90B1,JUN,PLCE1,NFKBIA,HSP90AB1,MAP2K2,TGFB1 (includes EG:21803),CLOCK,STAT5B,ADCY9,ACOX1,CREBBP,ADCY3,CD36,NFKB2,CAND1,PRKAG2,IL1RAPL1,ACVR2A |
| Calcium Signaling | 2.6E00 | 1.45E-01 | MYH10,MYL6,CALM1 (includes others),ATP2B1,MAPK3,HDAC10,EP300,GRINA,CABIN1,NFAT5,TRPV6,PPP3R1,HDAC7,ASPH,TPM4,CALR,ATP2C1,ITPR2,CREBBP,MEF2A,NFATC1,PPP3CC,RAP1A,ATP2B2,MYL9,MEF2D,ITPR3,PRKAG2,MYH9,CAMK2G |
| PI3K Signaling in B Lymphocytes | 2.58E00 | 1.68E-01 | CALM1 (includes others),ITPR2,MAPK3,PIK3R1,NFATC1,NFKB2,PPP3CC,CD79A,IKBKB,IKBKG,PLCE1,JUN,AKT1,NFKBIA,NFAT5,PRKCI,MAP2K2,PPP3R1,FOXO3,ITPR3,PIK3CD,PIK3R2,ELK1,CAMK2G |
| CD28 Signaling in T Helper Cells | 2.58E00 | 1.67E-01 | PTPN6,CALM1 (includes others),TRD@,ITPR2,CD4,PIK3R1,NFATC1,NFKB2,PPP3CC,IKBKB,IKBKG,JUN,AKT1,NFKBIA,NFAT5,MAP2K2,PTPN11,WAS,PPP3R1,ITPR3,PIK3CD,PIK3R2 |
| Role of Wnt/GSK-3β Signaling in the Pathogenesis of Influenza | 2.55E00 | 1.98E-01 | TCF4,CSNK1G1,CSNK1G3,CSNK1A1,CSNK1D,FZD1,TCF3,APC,FZD4,NCOA2,NCOA1,NCOA4,FZD5,CTNNB1,TCF7L2,IFNAR1 |
| Human Embryonic Stem Cell Pluripotency | 2.54E00 | 1.56E-01 | TCF4,PDGFA,PIK3R1,FGFR1,SMAD6,FGFR2,BMPR2,GSK3A,FZD1,NGF,TCF3,APC,PDGFB,AKT1,FZD4,TGFB1 (includes EG:21803),PDGFRA,FZD5,PIK3CD,PIK3R2,CTNNB1,TCF7L2,BMP1,PDGFRB |
| Cardiac Hypertrophy Signaling | 2.54E00 | 1.47E-01 | EIF2B4,MAP3K11,CALM1 (includes others),MYL6,PIK3R1,MAPK3,MAP3K5,EIF4E,EP300,GNB1,AKT1,JUN,PLCE1,RHOG,RHOB,MAP2K2,RHOT1,TGFB1 (includes EG:21803),PPP3R1,PIK3R2,MYL10,ADCY9,CREBBP,ADCY3,MEF2A,PPP3CC,GNG10,MYL9,GNAI2,MYL12A,MEF2D,GNB2,PRKAG2,PIK3CD,ELK1,HSPB1 |
| NF-κB Signaling | 2.54E00 | 1.59E-01 | AZI2,TRD@,PIK3R1,BMPR2,MAP4K4,NGF,EP300,TANK,IKBKB,IKBKG,IL36G,NFKBIA,AKT1,PDGFRA,CSNK2A1,LTBR,PIK3R2,EGFR,PDGFRB,TNFRSF1A,FGFR1,CREBBP,IL36A,FGFR2,NFKB2,IGF2R,DDR1,PIK3CD |
| Ephrin Receptor Signaling | 2.53E00 | 1.45E-01 | PDGFA,MAPK3,MAP4K4,CRK,GRINA,EPHB6,VEGFA,GNB1,SHC1 (includes EG:20416),AKT1,MAP2K2,SDC2,EFNB1,ITGB1,EPHB4,PAK4,PAK2,CFL1,CRKL,ITGA5,RAP1A,PDGFB,FGF1,GNG10,GNAI2,PTPN11,ABI1,WAS,GNB2 |
| Docosahexaenoic Acid (DHA) Signaling | 2.52E00 | 2.24E-01 | BCL2L1,AKT1,BAD,PIK3R1,GSK3A,PIK3CD,PIK3R2,BAX,PNPLA2,APP,BCL2 |
| Regulation of Actin-based Motility by Rho | 2.5E00 | 1.87E-01 | PAK4,MPRIP,PAK2,PFN1,MYL6,CFL1,ACTB,MYLK,MYL9,MYL12A,RHOG,RHOB,WAS,PIP5K1C,RHOT1,ARHGDIA,MYL10 |
| IL-8 Signaling | 2.49E00 | 1.5E-01 | PIK3R1,MAPK3,MAP4K4,CCND1,BCL2,VEGFA,GNB1,IKBKB,IKBKG,RHOG,AKT1,MAP2K2,RHOB,RHOT1,GPLD1,CYBB,PIK3R2,EGFR,PLD3,PAK2,MPO,MMP2,BAX,GNG10,GNAI2,BCL2L1,PRKCI,GNB2,PIK3CD |
| Production of Nitric Oxide and Reactive Oxygen Species in Macrophages | 2.47E00 | 1.44E-01 | MAP3K11,MAPK3,PIK3R1,MAP3K5,IKBKB,IKBKG,NFKBIA,AKT1,JUN,RHOG,RHOB,RHOT1,CYBB,PIK3R2,PTPN6,TNFRSF1A,CREBBP,MPO,PPP1R11,NFKB2,RAP1A,PPP2R1A,PRKCI,PPP2R4,CAT,PIK3CD,SIRPA |
| Cleavage and Polyadenylation of Pre-mRNA | 2.43E00 | 4.17E-01 | PAPOLA,CSTF1,NUDT21,CPSF1,CSTF3 |
| Tight Junction Signaling | 2.4E00 | 1.59E-01 | MYH10,MYL6,CPSF1,MYLK,MPDZ,AKT1,JUN,CLDN4,TGFB1 (includes EG:21803),ARHGEF2,CTNNB1,CSTF1,TNFRSF1A,ACTB,CSDA,NFKB2,MYL9,PPP2R1A,PRKCI,PPP2R4,NUDT21,PRKAG2,MYH9,SPTAN1,CSTF3,CLDN3 |
| ERK/MAPK Signaling | 2.39E00 | 1.47E-01 | BAD,PIK3R1,MAPK3,H3F3A/H3F3B,TLN1,CRK,RAPGEF4,EIF4E,SHC1 (includes EG:20416),MAP2K2,PIK3R2,ITGB1,PAK4,PAK2,YWHAB,CRKL,ITGA5,NFATC1,PPP1R11,RAP1A,ELF2,PPP2R1A,TLN2,PRKCI,DUSP1,PPP2R4,PRKAG2,PIK3CD,ELK1,HSPB1 |
| CREB Signaling in Neurons | 2.39E00 | 1.44E-01 | POLR2D,CALM1 (includes others),PIK3R1,MAPK3,POLR2J,EP300,GNB1,SHC1 (includes EG:20416),POLR2A,AKT1,PLCE1,MAP2K2,PIK3R2,ADCY9,GRM8,ITPR2,CREBBP,ADCY3,GNG10,GNAI2,GRM5,PRKCI,POLR2E,ITPR3,GNB2,PRKAG2,PIK3CD,ELK1,CAMK2G |
| Induction of Apoptosis by HIV1 | 2.38E00 | 1.97E-01 | TP53 (includes EG:22059),TNFRSF1A,BAX,MAP3K5,NFKB2,BCL2,IKBKB,BCL2L1,IKBKG,SLC25A6,NFKBIA,BBC3,FASLG |
| Aryl Hydrocarbon Receptor Signaling | 2.33E00 | 1.51E-01 | TP53 (includes EG:22059),MAPK3,SLC35A2,MDM2,NFKB2,BAX,CCND1,EP300,ALDH3B2,HSP90B1,ALDH1A1,JUN,NCOA2,HSP90AB1,TGFB1 (includes EG:21803),RARB,NFIB,DHFR,NFE2L2,ALDH5A1,CDK2,FASLG,HSPB1,ALDH7A1 |
| IL-15 Signaling | 2.32E00 | 1.94E-01 | STAT6,IL2RG,MAPK3,PIK3R1,NFKB2,BCL2,SHC1 (includes EG:20416),BCL2L1,AKT1,MAP2K2,PIK3CD,PIK3R2,STAT5B |
| Coagulation System | 2.31E00 | 2.37E-01 | KNG1,SERPINC1,PROS1,F5,PLAUR,F13B,FGB (includes EG:110135),TFPI,FGG |
| 4-1BB Signaling in T Lymphocytes | 2.3E00 | 2.35E-01 | IKBKB,IKBKG,JUN,NFKBIA,MAP2K2,MAPK3,MAP3K5,NFKB2 |
| TNFR1 Signaling | 2.29E00 | 2.08E-01 | TANK,CASP6,IKBKB,PAK4,IKBKG,JUN,PAK2,NFKBIA,TNFRSF1A,CASP2,NFKB2 |
| Gα12/13 Signaling | 2.29E00 | 1.64E-01 | MYL6,MAPK3,PIK3R1,MEF2A,ARHGEF1,NFKB2,MAP3K5,MYL9,IKBKB,MYL12A,IKBKG,JUN,NFKBIA,AKT1,MAP2K2,MEF2D,PIK3CD,PIK3R2,MYL10,ELK1,CTNNB1 |
| TGF-β Signaling | 2.27E00 | 1.8E-01 | INHA,MAPK3,CREBBP,SMAD6,BMPR2,PITX2,INHBB,BCL2,EP300,JUN,MAP2K2,TGFB1 (includes EG:21803),RUNX2,MAP4K1,TFE3,ACVR2A |
| Insulin Receptor Signaling | 2.26E00 | 1.64E-01 | SOCS3,FOXO4,EIF2B4,BAD,TRIP10,PIK3R1,MAPK3,CRKL,GSK3A,PPP1R11,CRK,VAMP2,PTPRF,EIF4E,SHC1 (includes EG:20416),AKT1,PRKCI,MAP2K2,PTPN11,FOXO3,PRKAG2,PIK3CD,PIK3R2 |
| Prolactin Signaling | 2.26E00 | 1.88E-01 | SOCS1,SOCS3,MAPK3,PIK3R1,CREBBP,SOCS6,EP300,SHC1 (includes EG:20416),PRKCI,JUN,MAP2K2,PTPN11,PIK3CD,PIK3R2,STAT5B |
| HER-2 Signaling in Breast Cancer | 2.21E00 | 1.85E-01 | TP53 (includes EG:22059),ITGB1,BAD,PIK3R1,MDM2,MMP2,GSK3A,MAP3K5,CCND1,PRKCI,AKT1,PIK3CD,ITGB4,PIK3R2,EGFR |
| PDGF Signaling | 2.21E00 | 1.9E-01 | PDGFA,MAPK3,CRKL,PIK3R1,CRK,PDGFB,SHC1 (includes EG:20416),JUN,MAP2K2,CSNK2A1,PDGFRA,PIK3CD,PIK3R2,ELK1,PDGFRB |
| Activation of IRF by Cytosolic Pattern Recognition Receptors | 2.19E00 | 1.81E-01 | PPIB,CREBBP,NFKB2,IRF3,TANK,IKBKB,IRF7,IKBKG,JUN,NFKBIA,STAT2,PIN1,IFNAR1 |
| ERK5 Signaling | 2.19E00 | 2.03E-01 | IL6ST,LIF,BAD,YWHAE,YWHAB,MEF2A,NGF,AKT1,PTPN11,MEF2D,FOXO3,FOSL1,EGFR |
| Rac Signaling | 2.19E00 | 1.54E-01 | ITGB1,PAK4,MAP3K11,PAK2,CFL1,MAPK3,PIK3R1,ITGA5,NFKB2,IQGAP2,PRKCI,JUN,ARFIP2,MAP2K2,PIP5K1C,CYBB,PIK3CD,PIK3R2,ELK1 |
| EGF Signaling | 2.15E00 | 2.12E-01 | SHC1 (includes EG:20416),JUN,ITPR2,MAPK3,PIK3R1,ITPR3,CSNK2A1,PIK3CD,PIK3R2,ELK1,EGFR |
| Non-Small Cell Lung Cancer Signaling | 2.14E00 | 1.77E-01 | TP53 (includes EG:22059),BAD,ITPR2,MAPK3,PIK3R1,CCND1,AKT1,MAP2K2,RARB,FOXO3,ITPR3,PIK3CD,PIK3R2,EGFR |
| Butanoate Metabolism | 2.12E00 | 1.23E-01 | ACAT2,RDH11,SUCLG2,SDHC,ELOVL6,AADAC,ALDH1A1,ACAT1,SDHD,DBT,EHHADH,PDHB,HMGCS1,ALDH5A1,HADH,ALDH7A1 |
| Aldosterone Signaling in Epithelial Cells | 2.11E00 | 1.53E-01 | CRYAB,HSPA1A/HSPA1B,PIK3R1,MAPK3,DNAJC3,HSPA4,HSP90B1,PLCE1,HSP90AB1,MAP2K2,DNAJC8,PIK3R2,DNAJC22,HSPB6,DNAJC9,ITPR2,HSPA9,DNAJB9,DNAJB14,PRKCI,DUSP1,PIP5K1C,ITPR3,PIK3CD,DNAJB5,HSPB1 |
| Melanoma Signaling | 2.1E00 | 2.17E-01 | TP53 (includes EG:22059),AKT1,BAD,MAP2K2,MAPK3,PIK3R1,MDM2,PIK3CD,PIK3R2,CCND1 |
| FLT3 Signaling in Hematopoietic Progenitor Cells | 2.09E00 | 1.89E-01 | STAT6,BAD,MAPK3,PIK3R1,EIF4E,SHC1 (includes EG:20416),AKT1,MAP2K2,PTPN11,PIK3CD,STAT2,PIK3R2,ELK1,STAT5B |
| T Cell Receptor Signaling | 2.07E00 | 1.65E-01 | CALM1 (includes others),MAPK3,CD4,PIK3R1,NFATC1,NFKB2,PPP3CC,SHB,IKBKB,IKBKG,NFKBIA,NFAT5,JUN,MAP2K2,PPP3R1,PIK3CD,PIK3R2,ELK1 |
| April Mediated Signaling | 2.06E00 | 2.09E-01 | IKBKB,IKBKG,NFAT5,JUN,NFKBIA,TNFSF13,NFATC1,NFKB2,ELK1 |
| 14-3-3-mediated Signaling | 2.05E00 | 1.67E-01 | YWHAE,BAD,TNFRSF1A,YWHAB,TUBB2C,MAPK3,PIK3R1,GSK3A,BAX,MAP3K5,TUBB,TUBB2B,PLCE1,JUN,AKT1,PRKCI,MAP2K2,PIK3CD,PIK3R2,ELK1 |
| Renin-Angiotensin Signaling | 2.05E00 | 1.59E-01 | ADCY9,PTPN6,PAK4,PAK2,ITPR2,MAPK3,PIK3R1,ADCY3,NFKB2,SHC1 (includes EG:20416),PRKCI,JUN,MAP2K2,CCL2,ITPR3,PRKAG2,PIK3CD,PIK3R2,ELK1,ACE |
| LPS-stimulated MAPK Signaling | 2.03E00 | 1.71E-01 | MAPK3,PIK3R1,MAP3K5,NFKB2,IKBKB,IKBKG,PRKCI,JUN,NFKBIA,MAP2K2,PIK3CD,LBP,PIK3R2,ELK1 |
| Thyroid Cancer Signaling | 2.03E00 | 2.17E-01 | TP53 (includes EG:22059),SHC1 (includes EG:20416),TCF4,MAP2K2,MAPK3,NGF,TCF3,CTNNB1,CCND1,TCF7L2 |
| DNA Methylation and Transcriptional Repression Signaling | 2.02E00 | 2.61E-01 | MECP2,DNMT3A,MTA1,CHD4,SAP18,RBBP4 |
| Myc Mediated Apoptosis Signaling | 2.01E00 | 1.97E-01 | TP53 (includes EG:22059),SHC1 (includes EG:20416),AKT1,BAD,YWHAE,YWHAB,PIK3R1,PIK3CD,PIK3R2,BAX,FASLG,BCL2 |
| **Canonical Pathways** | **-log(p-value)** | **Ratio** | **Molecules** |
| PI3K/AKT Signaling | 8.46E00 | 2.5E-01 | BAD,PIK3R1,MAPK3,LIMS1,ILK,GSK3A,MAP3K5,CCND1,EIF4E,BCL2,SHC1 (includes EG:20416),IKBKB,HSP90B1,IKBKG,NFKBIA,AKT1,HSP90AB1,MAP2K2,FOXO3,PIK3R2,CTNNB1,MCL1,TP53 (includes EG:22059),ITGB1,CDC37,RHEB,YWHAE,YWHAB,ITGA5,MDM2,NFKB2,BCL2L1,PPP2R1A,PPP2R4,PIK3CD |
| Molecular Mechanisms of Cancer | 7.93E00 | 1.79E-01 | BAD,MAPK3,PIK3R1,NCSTN,CDKN2C,GSK3A,ARHGEF1,RBL1,CCND1,RHOG,RHOB,MAP2K2,TGFB1 (includes EG:21803),HIPK2,FASLG,ADCY9,TP53 (includes EG:22059),CREBBP,ARHGEF17,NFKB2,AURKA,TCF3,RAP1A,APC,BCL2L1,PTPN11,PIK3CD,FZD5,NOTCH1,CDK2,CAMK2G,TCF4,BMPR2,PSENEN,CRK,FZD1,MAP3K5,E2F3,BCL2,EP300,CASP6,SHC1 (includes EG:20416),NFKBIA,NLK,JUN,AKT1,RHOT1,BBC3,ARHGEF2,PIK3R2,CTNNB1,BMP1,PAK4,PAK2,ADCY3,SMAD6,MDM2,BAX,APH1A,NBN,GNAI2,ARHGEF10,PRKCI,FZD4,NF1 (includes EG:18015),PRKAG2,ELK1,CTNND1 |
| PTEN Signaling | 6.98E00 | 2.42E-01 | FOXO4,BAD,PIK3R1,MAPK3,ILK,BMPR2,GSK3A,CCND1,BCL2,SHC1 (includes EG:20416),IKBKB,IKBKG,AKT1,MAP2K2,FOXO3,CSNK2A1,PDGFRA,PIK3R2,FASLG,PDGFRB,EGFR,ITGB1,FGFR1,ITGA5,FGFR2,NFKB2,IGF2R,DDR1,BCL2L1,PIK3CD |
| Apoptosis Signaling | 6.78E00 | 2.71E-01 | BAD,MAPK3,MAP4K4,MAP3K5,BCL2,ACIN1,CASP6,IKBKB,IKBKG,NFKBIA,MAP2K2,FASLG,MCL1,AIFM1,TP53 (includes EG:22059),TNFRSF1A,LMNA,BAX,NFKB2,CDK1,BCL2L1,CAPNS1,CASP2,CAPN1,CAPN2,SPTAN1 |
| Chronic Myeloid Leukemia Signaling | 5.71E00 | 2.38E-01 | BAD,PIK3R1,MAPK3,HDAC10,CRK,E2F3,RBL1,CCND1,CTBP1,IKBKB,IKBKG,AKT1,MAP2K2,TGFB1 (includes EG:21803),HDAC7,CTBP2,PIK3R2,STAT5B,TP53 (includes EG:22059),CRKL,MDM2,NFKB2,BCL2L1,PTPN11,PIK3CD |
| Integrin Signaling | 5.64E00 | 1.95E-01 | MPRIP,ARHGAP26,MAP3K11,PIK3R1,MAPK3,LIMS1,ILK,CRK,TLN1,MYLK,SHC1 (includes EG:20416),ARF6,AKT1,RHOG,RHOB,MAP2K2,RHOT1,PIK3R2,ITGB4,TSPAN4,ACTN1,ITGB1,PAK4,PAK2,CRKL,ACTB,ITGA5,RAP1A,ARHGAP5,MYL9,MYL12A,TLN2,CAPNS1,ARF3,WAS,CAPN1,ZYX,CAPN2,PIK3CD,ACTN4,ITGA7 |
| Androgen Signaling | 5.49E00 | 1.94E-01 | POLR2D,CALM1 (includes others),MAPK3,POLR2J,CCND1,EP300,GNB1,HSPA4,SHC1 (includes EG:20416),TGFB1I1,POLR2A,JUN,AR,NCOA2,NCOA4,CALR,CREBBP,NFKB2,GNG10,GNAI2,KAT2B,PRKCI,POLR2E,GTF2E1,NCOA1,GNB2,PRKAG2,GTF2H1 |
| Acute Phase Response Signaling | 5.38E00 | 2.02E-01 | IL6ST,SOCS3,SOCS1,TCF4,saa3p,PIK3R1,APOA2,MAPK3,SOCS6,AMBP,CP,MAP3K5,FGG,IKBKB,SHC1 (includes EG:20416),IKBKG,IL36G,NFKBIA,JUN,AKT1,MAP2K2,APCS,FGB (includes EG:110135),LBP,PIK3R2,TTR,TNFRSF1A,AHSG,IL36A,NFKB2,TCF3,ALB,TF,PTPN11,PIK3CD,ELK1 |
| p53 Signaling | 5.26E00 | 2.5E-01 | TP53 (includes EG:22059),GADD45B,PIK3R1,CSNK1D,MDM2,BAX,CCND1,TP53I3,EP300,BCL2,CASP6,BCL2L1,KAT2B,JUN,AKT1,BBC3,STAG1,ADCK3,PIK3CD,PIK3R2,PML,HIPK2,CTNNB1,CDK2 |
| PPAR Signaling | 5E00 | 2.24E-01 | TNFRSF1A,PDGFA,MAPK3,CREBBP,IL36A,MAP4K4,NFKB2,PDGFB,EP300,SHC1 (includes EG:20416),IKBKB,IL36G,IKBKG,HSP90B1,JUN,NFKBIA,MAP2K2,HSP90AB1,PDGFRA,NCOA1,IL1RAPL1,STAT5B,CITED2,PDGFRB |
| Renal Cell Carcinoma Signaling | 5E00 | 2.7E-01 | RBX1,PAK4,PAK2,PIK3R1,MAPK3,CREBBP,CRK,RAP1A,PDGFB,EP300,VEGFA,AKT1,JUN,MAP2K2,PTPN11,CUL2,TGFB1 (includes EG:21803),PIK3CD,PIK3R2,FH |
| Wnt/β-catenin Signaling | 4.85E00 | 2.01E-01 | TCF4,CSNK1G1,SOX15,CSNK1A1,ILK,GSK3A,FZD1,CCND1,EP300,JUN,AKT1,NLK,TGFB1 (includes EG:21803),RARB,CSNK2A1,SFRP5,MAP4K1,CTNNB1,TP53 (includes EG:22059),SOX4,APPL2,CSNK1G3,CREBBP,CSNK1D,MDM2,TCF3,APC,PPP2R1A,FZD4,PPP2R4,NR5A2,FZD5,PIN1,ACVR2A,TCF7L2 |
| Glucocorticoid Receptor Signaling | 4.72E00 | 1.63E-01 | TAF11 (includes EG:309638),POLR2D,HSPA1A/HSPA1B,MAPK3,PIK3R1,POLR2J,TAF13 (includes EG:310784),FGG,BCL2,EP300,HSPA4,SHC1 (includes EG:20416),IKBKB,IKBKG,HSP90B1,NFKBIA,AR,POLR2A,NFAT5,AKT1,JUN,HSP90AB1,NCOA2,CCL2,MAP2K2,TGFB1 (includes EG:21803),BAG1,PPP3R1,FOXO3,PIK3R2,STAT5B,TAF6,HSPA9,CREBBP,NFATC1,PPP3CC,KAT2B,BCL2L1,SUMO1,DUSP1,SMARCA2,POLR2E,GTF2E1,NCOA1,PRKAG2,GTF2H1,PIK3CD,ELK1 |
| PAK Signaling | 4.4E00 | 2.06E-01 | ITGB1,PAK4,PAK2,ARHGAP10,MYL6,CFL1,PDGFA,PIK3R1,MAPK3,PAK1IP1,ITGA5,PDGFB,MYLK,MYL9,SHC1 (includes EG:20416),MYL12A,MAP2K2,PDGFRA,PIK3CD,PIK3R2,MYL10,PDGFRB |
| Prostate Cancer Signaling | 4.37E00 | 2.16E-01 | TP53 (includes EG:22059),BAD,SRD5A1,MAPK3,PIK3R1,CREBBP,MDM2,NFKB2,CCND1,BCL2,HSP90B1,AKT1,NFKBIA,AR,MAP2K2,HSP90AB1,NKX3-1,PIK3CD,PIK3R2,CTNNB1,CDK2 |
| Role of Osteoblasts, Osteoclasts and Chondrocytes in Rheumatoid Arthritis | 4.35E00 | 1.71E-01 | TCF4,CALM1 (includes others),BAD,PIK3R1,MAPK3,CSNK1A1,BMPR2,MAP3K5,FZD1,BCL2,IKBKB,IL36G,IKBKG,NFKBIA,NFAT5,AKT1,JUN,RUNX2,TGFB1 (includes EG:21803),PPP3R1,SFRP5,PIK3R2,CTNNB1,BMP1,ITGB1,TNFRSF1A,IL36A,SMAD6,ITGA5,NFATC1,PPP3CC,TCF3,CSF1R,APC,COL1A1,FZD4,CSF1 (includes EG:12977),FZD5,PIK3CD,IL1RAPL1,TCF7L2 |
| Phospholipase C Signaling | 4.3E00 | 1.65E-01 | MPRIP,TRD@,CALM1 (includes others),MYL6,MAPK3,HDAC10,ARHGEF1,CD79A,EP300,GNB1,SHC1 (includes EG:20416),NFAT5,RHOG,PLCE1,RHOB,MAP2K2,RHOT1,PPP3R1,GPLD1,HDAC7,ARHGEF2,MYL10,ADCY9,ITGB1,PLD3,ITPR2,ADCY3,CREBBP,MEF2A,ITGA5,NFATC1,ARHGEF17,PPP3CC,NFKB2,RAP1A,GNG10,MYL9,MYL12A,PRKCI,ARHGEF10,MEF2D,ITPR3,GNB2 |
| Hepatic Fibrosis / Hepatic Stellate Cell Activation | 4.23E00 | 1.97E-01 | IGFBP4,MYH10,MYL6,PDGFA,BCL2,VEGFA,CCL2,TGFB1 (includes EG:21803),TIMP1,PDGFRA,LBP,FASLG,EGFR,PDGFRB,TNFRSF1A,FGFR1,FGFR2,MMP2,BAX,NFKB2,IFNAR2,PDGFB,FGF1,MYL9,COL1A1,CSF1 (includes EG:12977),MYH9,IL1RAPL1,IFNAR1 |
| Estrogen Receptor Signaling | 4.18E00 | 1.99E-01 | TAF11 (includes EG:309638),POLR2D,MAPK3,POLR2J,H3F3A/H3F3B,TAF13 (includes EG:310784),EP300,CTBP1,SPEN,SHC1 (includes EG:20416),CDK8,POLR2A,MAP2K2,NCOA2,MED15,CTBP2,TAF6,CREBBP,MED6 (includes EG:10001),KAT2B,POLR2E,MED13L,GTF2E1,NCOA1,GTF2H1,MED4 (includes EG:29079),CARM1 |
| Erythropoietin Signaling | 4.08E00 | 2.31E-01 | EPO,SOCS1,SOCS3,PTPN6,EPOR,MAPK3,PIK3R1,NFKB2,SHC1 (includes EG:20416),NFKBIA,PRKCI,AKT1,JUN,MAP2K2,PIK3CD,PIK3R2,STAT5B,ELK1 |
| Huntington's Disease Signaling | 4.01E00 | 1.68E-01 | POLR2D,HSPA1A/HSPA1B,REST,MAPK3,PIK3R1,POLR2J,HDAC10,NGF,EP300,GNB1,HSPA4,CASP6,SHC1 (includes EG:20416),ARFIP2,POLR2A,AKT1,JUN,CPLX2,HDAC7,VAMP3,PIK3R2,NAPA,EGFR,TP53 (includes EG:22059),HSPA9,CREBBP,BAX,GNG10,GRM5,RCOR1,BCL2L1,PRKCI,CAPNS1,POLR2E,CASP2,CAPN1,GNB2,STX16,CAPN2,PIK3CD |
| Glioblastoma Multiforme Signaling | 4E00 | 1.83E-01 | PDGFA,PIK3R1,MAPK3,FZD1,E2F3,CCND1,SHC1 (includes EG:20416),RHOG,AKT1,PLCE1,MAP2K2,RHOB,RHOT1,PDGFRA,PIK3R2,CTNNB1,EGFR,PDGFRB,TP53 (includes EG:22059),ITPR2,MDM2,TCF3,PDGFB,APC,FZD4,NF1 (includes EG:18015),ITPR3,FZD5,PIK3CD,CDK2 |
| NRF2-mediated Oxidative Stress Response | 3.99E00 | 1.77E-01 | AKR7A2,RBX1,PPIB,PIK3R1,MAPK3,DNAJC3,MAP3K5,SOD3,EP300,JUN,AKT1,MAP2K2,DNAJC8,PMF1,FOSL1,DNAJA2,JUND,TXN (includes EG:116484),PIK3R2,NFE2L2,DNAJC9,ACTB,CREBBP,SLC35A2,JUNB,DNAJB9,DNAJB14,PRKCI,STIP1,CAT,PIK3CD,PTPLAD1,DNAJB5,EPHX1 |
| FAK Signaling | 3.99E00 | 2.06E-01 | ITGB1,PAK4,PAK2,ARHGAP26,ACTB,PIK3R1,MAPK3,ITGA5,CRK,TLN1,CAPNS1,TLN2,AKT1,MAP2K2,WAS,CAPN1,CAPN2,PIK3CD,PIK3R2,TNS1,EGFR |
| Reelin Signaling in Neurons | 3.85E00 | 2.32E-01 | ITGB1,MAP3K11,PIK3R1,CRKL,MAPK8IP2,ITGA5,ARHGEF1,RELN,MAPK8IP3,FRK,APP,APBB1,YES1,AKT1,ARHGEF10,MAP4K1,ARHGEF2,PIK3CD,PIK3R2 |
| Hereditary Breast Cancer Signaling | 3.83E00 | 1.94E-01 | POLR2D,GADD45B,PIK3R1,POLR2J,HDAC10,DDB2,CCND1,RAD50,EP300,POLR2A,AKT1,HDAC7,RFC2,PIK3R2,TP53 (includes EG:22059),CREBBP,RFC5,CDK1,NBN,PALB2,SMARCA2,POLR2E,H2AFX,PIK3CD,RFC3 |
| JAK/Stat Signaling | 3.8E00 | 2.5E-01 | STAT6,SOCS1,SOCS3,PTPN6,PIAS2,MAPK3,PIK3R1,SOCS6,SHC1 (includes EG:20416),AKT1,PTPN11,MAP2K2,STAT2,PIK3CD,PIK3R2,STAT5B |
| Neuregulin Signaling | 3.77E00 | 2.06E-01 | ITGB1,BAD,PIK3R1,MAPK3,CRKL,ITGA5,CRK,SHC1 (includes EG:20416),ERBB2IP,HSP90B1,PRKCI,AKT1,PICK1,MAP2K2,PTPN11,HSP90AB1,PIK3R2,STAT5B,ELK1,EGFR,MATK |
| Clathrin-mediated Endocytosis Signaling | 3.75E00 | 1.8E-01 | RAB4A,PDGFA,PIK3R1,GAK,VEGFA,CD2AP,ARF6,AP1G2,RAB11B,PPP3R1,CSNK2A1,PIK3R2,SH3GLB2,ITGB4,HGS,ITGB1,MYO6,AP2M1,EPN1,ACTB,ITGA5,MDM2,PPP3CC,PDGFB,FGF1,FGF21,ARRB2,TF,PIP5K1C,CLTA,PIK3CD |
| Glioma Signaling | 3.75E00 | 1.96E-01 | TP53 (includes EG:22059),CALM1 (includes others),PDGFA,PIK3R1,MAPK3,CDKN2C,MDM2,E2F3,RBL1,CCND1,IGF2R,PDGFB,SHC1 (includes EG:20416),AKT1,PRKCI,MAP2K2,PDGFRA,PIK3CD,PIK3R2,EGFR,PDGFRB,CAMK2G |
| IL-6 Signaling | 3.7E00 | 2.1E-01 | IL6ST,SOCS1,TNFRSF1A,MAPK3,IL36A,MAP4K4,NFKB2,COL1A1,SHC1 (includes EG:20416),IKBKB,IKBKG,IL36G,JUN,NFKBIA,MAP2K2,PTPN11,CSNK2A1,IL1RAPL1,LBP,ELK1,HSPB1 |
| Role of NFAT in Regulation of the Immune Response | 3.68E00 | 1.65E-01 | CSNK1G1,CALM1 (includes others),TRD@,PIK3R1,CD4,MAPK3,CSNK1A1,GSK3A,CD79A,CABIN1,GNB1,IKBKB,IKBKG,NFKBIA,JUN,AKT1,NFAT5,MAP2K2,PPP3R1,PIK3R2,ITPR2,CSNK1G3,CSNK1D,MEF2A,NFATC1,PPP3CC,NFKB2,GNG10,GNAI2,MEF2D,ITPR3,GNB2,PIK3CD |
| IGF-1 Signaling | 3.68E00 | 2.06E-01 | IGFBP4,SOCS1,SOCS3,YWHAE,BAD,YWHAB,MAPK3,PIK3R1,SOCS6,SHC1 (includes EG:20416),PRKCI,AKT1,JUN,MAP2K2,PTPN11,FOXO3,CSNK2A1,PRKAG2,PIK3CD,PIK3R2,ELK1,CYR61 |
| SAPK/JNK Signaling | 3.63E00 | 2.06E-01 | TP53 (includes EG:22059),MAP3K11,DUSP8,TRD@,PIK3R1,CRKL,MAPK8IP2,MAP4K4,CRK,NFATC1,MAP3K5,MAPK8IP3,GNB1,SHC1 (includes EG:20416),MINK1,JUN,DUSP10,MAP4K1,PIK3CD,PIK3R2,ELK1 |
| Germ Cell-Sertoli Cell Junction Signaling | 3.49E00 | 1.8E-01 | MAP3K11,PIK3R1,MAPK3,ILK,MAP3K5,TUBB,TUBB2B,AGGF1,AKT1,RHOG,MAP2K2,RHOB,RHOT1,TGFB1 (includes EG:21803),MTMR2,PIK3R2,JUP,CTNNB1,ACTN1,ITGB1,EPN1,PAK4,PAK2,TUBB2C,TNFRSF1A,ACTB,ZYX,PIK3CD,ACTN4,CTNND1 |
| IL-3 Signaling | 3.48E00 | 2.3E-01 | STAT6,PTPN6,BAD,MAPK3,CRKL,PIK3R1,PPP3CC,SHC1 (includes EG:20416),PRKCI,AKT1,JUN,MAP2K2,PPP3R1,PIK3CD,PIK3R2,STAT5B,ELK1 |
| RAR Activation | 3.43E00 | 1.71E-01 | PIK3R1,ADH1C (includes EG:11522),MAP3K5,EP300,VEGFA,ALDH1A1,AKT1,JUN,TGFB1 (includes EG:21803),RARB,CSNK2A1,SORBS3,PIK3R2,STAT5B,CITED2,ADCY9,RDH14,RDH11,CREBBP,ADCY3,SMAD6,NFKB2,KAT2B,PRKCI,SMARCA2,DUSP1,NCOA1,PRKAG2,GTF2H1,PIK3CD,PML,CARM1 |
| B Cell Receptor Signaling | 3.36E00 | 1.79E-01 | MAP3K11,BAD,CALM1 (includes others),PIK3R1,MAPK3,GSK3A,MAP3K5,CD79A,SHC1 (includes EG:20416),IKBKB,IKBKG,AKT1,JUN,NFAT5,NFKBIA,MAP2K2,PPP3R1,CD22,PIK3R2,PTPN6,NFATC1,PPP3CC,NFKB2,BCL2L1,PTPN11,PIK3CD,ELK1,CAMK2G |
| Hypoxia Signaling in the Cardiovascular System | 3.3E00 | 2.35E-01 | TP53 (includes EG:22059),EPO,CSNK1D,MDM2,UBE2D1,EP300,VEGFA,HSP90B1,AKT1,JUN,NFKBIA,SUMO1,HSP90AB1,UBE2B,UBE2G1,UBE2J1 |
| IL-2 Signaling | 3.25E00 | 2.41E-01 | SOCS1,IL2RG,MAPK3,PIK3R1,SHC1 (includes EG:20416),AKT1,JUN,MAP2K2,PTPN11,CSNK2A1,PIK3CD,PIK3R2,ELK1,STAT5B |
| Pancreatic Adenocarcinoma Signaling | 3.25E00 | 1.85E-01 | TP53 (includes EG:22059),PLD3,BAD,PIK3R1,MAPK3,MDM2,NFKB2,E2F3,CCND1,BCL2,VEGFA,BCL2L1,AKT1,MAP2K2,TGFB1 (includes EG:21803),GPLD1,PIK3CD,PIK3R2,ELK1,NOTCH1,CDK2,EGFR |
| Thrombin Signaling | 3.23E00 | 1.64E-01 | MPRIP,MYL6,PIK3R1,MAPK3,ARHGEF1,MYLK,GNB1,SHC1 (includes EG:20416),IKBKB,RHOG,AKT1,PLCE1,RHOB,MAP2K2,RHOT1,ARHGEF2,PIK3R2,MYL10,EGFR,ADCY9,ITPR2,ADCY3,NFKB2,GNG10,MYL9,GNAI2,MYL12A,ARHGEF10,PRKCI,ITPR3,GNB2,PIK3CD,ELK1,CAMK2G |
| Extrinsic Prothrombin Activation Pathway | 3.2E00 | 3.5E-01 | SERPINC1,PROS1,F5,F13B,FGB (includes EG:110135),TFPI,FGG |
| Protein Kinase A Signaling | 3.17E00 | 1.49E-01 | MYH10,BAD,MYL6,MAPK3,GSK3A,MYLK,GNB1,PLCE1,MAP2K2,TGFB1 (includes EG:21803),PPP3R1,MYL10,ADCY9,YWHAE,YWHAB,ITPR2,PDE9A,CREBBP,PPP1R11,NFKB2,PPP3CC,TCF3,RAP1A,GNG10,MYL9,AKAP13,MYL12A,ITPR3,GNB2,CAMK2G,ANAPC2,TCF4,CALM1 (includes others),H3F3A/H3F3B,AKAP7,NFAT5,NFKBIA,FLNA,CTNNB1,ADCY3,PYGL,NFATC1,GNAI2,PRKCI,PRKAG2,ADD1,ELK1,TCF7L2,CDC27 |
| GM-CSF Signaling | 3.11E00 | 2.24E-01 | MAPK3,PIK3R1,PPP3CC,CCND1,SHC1 (includes EG:20416),BCL2L1,AKT1,MAP2K2,PTPN11,PPP3R1,PIK3CD,PIK3R2,ELK1,STAT5B,CAMK2G |
| Role of CHK Proteins in Cell Cycle Checkpoint Control | 3.1E00 | 2.86E-01 | TP53 (includes EG:22059),RAD17 (includes EG:19356),RFC2,RFC5,E2F3,CDK1,RAD50,CDK2,RFC3,NBN |
| Role of Tissue Factor in Cancer | 3.08E00 | 1.93E-01 | TP53 (includes EG:22059),ITGB1,CFL1,PIK3R1,MAPK3,PLAUR,FRK,EIF4E,FGG,VEGFA,BCL2L1,YES1,ARRB2,AKT1,PTPN11,CSF1 (includes EG:12977),FGB (includes EG:110135),PIK3CD,PIK3R2,STAT5B,CYR61,EGFR |
| Actin Cytoskeleton Signaling | 3.05E00 | 1.51E-01 | MYH10,PFN1,MPRIP,MYL6,PDGFA,PIK3R1,MAPK3,ARHGEF1,CRK,MYLK,SHC1 (includes EG:20416),IQGAP2,MAP2K2,LBP,PIK3R2,MYL10,ACTN1,MATK,ITGB1,PAK4,PAK2,CFL1,ACTB,CRKL,ITGA5,PDGFB,APC,FGF1,MYL9,FGF21,MYL12A,PIP5K1C,WAS,MYH9,PIK3CD,ACTN4 |
| Protein Ubiquitination Pathway | 3.04E00 | 1.53E-01 | PSMB3,B2M,ANAPC2,USP24,RBX1,CRYAB,USP5,HSPA1A/HSPA1B,UBE3B,UBR2 (includes EG:224826),DNAJC3,HSPA4,HSP90B1,HSP90AB1,UBE2B,BAG1,PSMC6,DNAJC8,UCHL5,NEDD4L,PSMA2,DNAJC22,UBE2J1,AMFR,HSPB6,DNAJC9,USP15,HSPA9,MDM2,DNAJB9,USP33,DNAJB14,UBE2D1,FZR1,CUL2,UBE2G1,PSMA4,USP46,UBA1,USP25,DNAJB5,HSPB1 |
| Polyamine Regulation in Colon Cancer | 3.02E00 | 2.76E-01 | TCF4,AZIN1,PSMF1,SAT1,PSME4,CTNNB1,PSME3,APC |
| ILK Signaling | 3.01E00 | 1.66E-01 | MYH10,MYL6,PIK3R1,MAPK3,ILK,GSK3A,CCND1,VEGFA,TGFB1I1,RHOG,AKT1,JUN,RHOB,FLNA,RHOT1,PIK3R2,ITGB4,CTNNB1,ACTN1,ITGB1,CFL1,TNFRSF1A,ACTB,FERMT2,SNAI1,NFKB2,MYL9,PPP2R1A,PPP2R4,MYH9,PIK3CD,ACTN4 |
| ATM Signaling | 2.98E00 | 2.41E-01 | TP53 (includes EG:22059),SMC3,GADD45B,MDM2,CDK1,RAD50,SMC1A,NBN,JUN,NFKBIA,SMC2,H2AFX,CDK2 |
| Amyloid Processing | 2.98E00 | 2.32E-01 | MAPK3,CSNK1D,CSNK1A1,NCSTN,PSENEN,APP,APH1A,AKT1,CAPNS1,CAPN1,CSNK2A1,PRKAG2,CAPN2 |
| Role of NFAT in Cardiac Hypertrophy | 2.97E00 | 1.54E-01 | IL6ST,LIF,CALM1 (includes others),PIK3R1,MAPK3,CSNK1A1,HDAC10,EP300,CABIN1,GNB1,SHC1 (includes EG:20416),AKT1,PLCE1,MAP2K2,TGFB1 (includes EG:21803),PPP3R1,HDAC7,PIK3R2,ADCY9,ITPR2,ADCY3,MEF2A,PPP3CC,GNG10,GNAI2,PRKCI,MEF2D,ITPR3,GNB2,PRKAG2,PIK3CD,CAMK2G |
| TR/RXR Activation | 2.96E00 | 1.98E-01 | AKR1C1/AKR1C2,GPS2,PIK3R1,BCL3,MDM2,ME1,EP300,KLF9,SLC16A3,UCP3,AKT1,NCOA2,SREBF2,DIO1,NCOA1,NCOA4,PIK3CD,PIK3R2,SYT12 |
| Colorectal Cancer Metastasis Signaling | 2.94E00 | 1.51E-01 | IL6ST,TCF4,BAD,PIK3R1,MAPK3,FZD1,CCND1,VEGFA,GNB1,RHOG,AKT1,JUN,RHOB,MMP25,MAP2K2,RHOT1,TGFB1 (includes EG:21803),PIK3R2,CTNNB1,EGFR,MMP19,TP53 (includes EG:22059),ADCY9,TNFRSF1A,ADRBK2,ADCY3,MMP2,BAX,NFKB2,TCF3,APC,GNG10,BCL2L1,FZD4,GNB2,PRKAG2,FZD5,PIK3CD,TCF7L2 |
| Death Receptor Signaling | 2.92E00 | 2.15E-01 | TNFRSF1A,MAP4K4,MAP3K5,NFKB2,BCL2,TANK,CASP6,IKBKB,IKBKG,NFKBIA,TNFSF12,CASP2,FASLG,HSPB1 |
| VEGF Signaling | 2.9E00 | 1.92E-01 | EIF2S3,EIF2B4,YWHAE,BAD,ACTB,MAPK3,PIK3R1,EIF1,BCL2,VEGFA,SHC1 (includes EG:20416),BCL2L1,AKT1,MAP2K2,FOXO3,PIK3CD,PIK3R2,ACTN4,ACTN1 |
| Role of JAK2 in Hormone-like Cytokine Signaling | 2.88E00 | 2.7E-01 | EPO,SHC1 (includes EG:20416),SOCS1,SOCS3,PTPN6,PTPN11,EPOR,SOCS6,STAT5B,SIRPA |
| fMLP Signaling in Neutrophils | 2.87E00 | 1.72E-01 | CALM1 (includes others),ITPR2,MAPK3,PIK3R1,NFATC1,NFKB2,PPP3CC,GNG10,GNB1,GNAI2,NFKBIA,NFAT5,PRKCI,MAP2K2,WAS,PPP3R1,ITPR3,GNB2,CYBB,PIK3CD,PIK3R2,ELK1 |
| Angiopoietin Signaling | 2.82E00 | 2.03E-01 | PAK4,PAK2,GRB14,BAD,PIK3R1,CRK,NFKB2,IKBKB,IKBKG,NFKBIA,AKT1,PTPN11,PIK3CD,PIK3R2,STAT5B |
| Endometrial Cancer Signaling | 2.82E00 | 2.28E-01 | TP53 (includes EG:22059),BAD,MAPK3,PIK3R1,ILK,CCND1,AKT1,MAP2K2,FOXO3,PIK3CD,PIK3R2,ELK1,CTNNB1 |
| CXCR4 Signaling | 2.81E00 | 1.66E-01 | MYL6,PIK3R1,MAPK3,CD4,CRK,GNB1,RHOG,AKT1,JUN,MAP2K2,RHOB,RHOT1,PIK3R2,MYL10,ADCY9,PAK4,PAK2,ITPR2,ADCY3,GNG10,GNAI2,MYL9,MYL12A,PRKCI,ITPR3,GNB2,PIK3CD,ELK1 |
| Breast Cancer Regulation by Stathmin1 | 2.81E00 | 1.57E-01 | CALM1 (includes others),PIK3R1,MAPK3,ARHGEF1,E2F3,TUBB,TUBB2B,GNB1,SHC1 (includes EG:20416),MAP2K2,RB1CC1,ARHGEF2,PIK3R2,TP53 (includes EG:22059),ADCY9,TUBB2C,ITPR2,ADCY3,ARHGEF17,PPP1R11,CDK1,GNG10,GNAI2,PPP2R1A,ARHGEF10,PRKCI,PPP2R4,ITPR3,GNB2,PRKAG2,PIK3CD,CDK2,CAMK2G |
| Axonal Guidance Signaling | 2.8E00 | 1.32E-01 | GLI2,PFN1,MYL6,MAPK3,PIK3R1,TUBB,VEGFA,GNB1,MAP2K2,PPP3R1,PLXNB2,MYL10,CFL1,CRKL,ITGA5,PPP3CC,RAP1A,PDGFB,GNG10,MYL9,MYL12A,PTPN11,RTN4,GNB2,PIK3CD,FZD5,PDGFA,SEMA6A,CRK,FZD1,NGF,EIF4E,TUBB2B,EPHB6,SHC1 (includes EG:20416),AKT1,NFAT5,PLXNA1,SDC2,EFNB1,PIK3R2,SHANK2,BMP1,ITGB1,EPHB4,PAK4,PAK2,NRP2,ADAM2,TUBB2C,NFATC1,PLXND1,GNAI2,PRKCI,FZD4,WAS,PRKAG2 |
| RANK Signaling in Osteoclasts | 2.79E00 | 1.89E-01 | MAP3K11,CALM1 (includes others),MAPK3,PIK3R1,NFATC1,PPP3CC,MAP3K5,NFKB2,IKBKB,IKBKG,NFKBIA,AKT1,JUN,MAP2K2,PPP3R1,PIK3CD,PIK3R2,ELK1 |
| Macropinocytosis Signaling | 2.75E00 | 1.97E-01 | ITGB1,PDGFA,PIK3R1,ITGA5,NGF,CSF1R,PDGFB,ARF6,PRKCI,ABI1,CSF1 (includes EG:12977),PIK3CD,ITGB4,PIK3R2,ACTN4 |
| Role of Macrophages, Fibroblasts and Endothelial Cells in Rheumatoid Arthritis | 2.73E00 | 1.38E-01 | IL6ST,SOCS1,SOCS3,TCF4,CALM1 (includes others),PDGFA,MAPK3,PIK3R1,IL32,CSNK1A1,FZD1,CCND1,VEGFA,IKBKB,IKBKG,IL36G,C5AR1,NFKBIA,NLK,NFAT5,AKT1,JUN,PLCE1,CCL2,MAP2K2,TGFB1 (includes EG:21803),PPP3R1,SFRP5,PIK3R2,LTBR,CTNNB1,TNFRSF1A,IL36A,NFATC1,PPP3CC,TCF3,PDGFB,APC,PRKCI,FZD4,CSF1 (includes EG:12977),FZD5,PIK3CD,IL1RAPL1,TCF7L2,CAMK2G |
| iCOS-iCOSL Signaling in T Helper Cells | 2.7E00 | 1.72E-01 | IL2RG,BAD,CALM1 (includes others),TRD@,ITPR2,CD4,PIK3R1,NFATC1,NFKB2,PPP3CC,SHC1 (includes EG:20416),IKBKB,IKBKG,AKT1,NFAT5,NFKBIA,PPP3R1,ITPR3,PIK3CD,PIK3R2,CAMK2G |
| Intrinsic Prothrombin Activation Pathway | 2.7E00 | 2.65E-01 | COL1A1,KNG1,KLK1,SERPINC1,PROS1,F5,F13B,FGB (includes EG:110135),FGG |
| Lymphotoxin β Receptor Signaling | 2.66E00 | 2.13E-01 | MAPK3,PIK3R1,CREBBP,NFKB2,EP300,IKBKB,BCL2L1,IKBKG,AKT1,NFKBIA,PIK3CD,LTBR,PIK3R2 |
| HIF1α Signaling | 2.64E00 | 1.85E-01 | TP53 (includes EG:22059),EPO,EGLN2,RBX1,MAPK3,PIK3R1,CREBBP,MMP2,MDM2,EP300,P4HTM,VEGFA,JUN,AKT1,MMP25,CUL2,NCOA1,PIK3CD,PIK3R2,MMP19 |
| Acute Myeloid Leukemia Signaling | 2.61E00 | 1.95E-01 | TCF4,BAD,MAPK3,PIK3R1,NFKB2,TCF3,CCND1,CSF1R,AKT1,MAP2K2,PIK3CD,JUP,PIK3R2,PML,STAT5B,TCF7L2 |
| Lysine Degradation | 2.61E00 | 1.22E-01 | SETD8,AASDHPPT,ACAT2,RDH11,ELOVL6,EP300,KAT2B,ALDH1A1,SMYD3,EHMT2,ACAT1,DBT,EHHADH,SHMT2,PLOD3,HADH,ALDH7A1 |
| PPARα/RXRα Activation | 2.61E00 | 1.56E-01 | CYP2C9,MAPK3,APOA2,MAP4K4,ADIPOR1,ABCA1,EP300,SHC1 (includes EG:20416),IKBKB,IKBKG,HSP90B1,JUN,PLCE1,NFKBIA,HSP90AB1,MAP2K2,TGFB1 (includes EG:21803),CLOCK,STAT5B,ADCY9,ACOX1,CREBBP,ADCY3,CD36,NFKB2,CAND1,PRKAG2,IL1RAPL1,ACVR2A |
| Calcium Signaling | 2.6E00 | 1.45E-01 | MYH10,MYL6,CALM1 (includes others),ATP2B1,MAPK3,HDAC10,EP300,GRINA,CABIN1,NFAT5,TRPV6,PPP3R1,HDAC7,ASPH,TPM4,CALR,ATP2C1,ITPR2,CREBBP,MEF2A,NFATC1,PPP3CC,RAP1A,ATP2B2,MYL9,MEF2D,ITPR3,PRKAG2,MYH9,CAMK2G |
| PI3K Signaling in B Lymphocytes | 2.58E00 | 1.68E-01 | CALM1 (includes others),ITPR2,MAPK3,PIK3R1,NFATC1,NFKB2,PPP3CC,CD79A,IKBKB,IKBKG,PLCE1,JUN,AKT1,NFKBIA,NFAT5,PRKCI,MAP2K2,PPP3R1,FOXO3,ITPR3,PIK3CD,PIK3R2,ELK1,CAMK2G |
| CD28 Signaling in T Helper Cells | 2.58E00 | 1.67E-01 | PTPN6,CALM1 (includes others),TRD@,ITPR2,CD4,PIK3R1,NFATC1,NFKB2,PPP3CC,IKBKB,IKBKG,JUN,AKT1,NFKBIA,NFAT5,MAP2K2,PTPN11,WAS,PPP3R1,ITPR3,PIK3CD,PIK3R2 |
| Role of Wnt/GSK-3β Signaling in the Pathogenesis of Influenza | 2.55E00 | 1.98E-01 | TCF4,CSNK1G1,CSNK1G3,CSNK1A1,CSNK1D,FZD1,TCF3,APC,FZD4,NCOA2,NCOA1,NCOA4,FZD5,CTNNB1,TCF7L2,IFNAR1 |
| Human Embryonic Stem Cell Pluripotency | 2.54E00 | 1.56E-01 | TCF4,PDGFA,PIK3R1,FGFR1,SMAD6,FGFR2,BMPR2,GSK3A,FZD1,NGF,TCF3,APC,PDGFB,AKT1,FZD4,TGFB1 (includes EG:21803),PDGFRA,FZD5,PIK3CD,PIK3R2,CTNNB1,TCF7L2,BMP1,PDGFRB |
| Cardiac Hypertrophy Signaling | 2.54E00 | 1.47E-01 | EIF2B4,MAP3K11,CALM1 (includes others),MYL6,PIK3R1,MAPK3,MAP3K5,EIF4E,EP300,GNB1,AKT1,JUN,PLCE1,RHOG,RHOB,MAP2K2,RHOT1,TGFB1 (includes EG:21803),PPP3R1,PIK3R2,MYL10,ADCY9,CREBBP,ADCY3,MEF2A,PPP3CC,GNG10,MYL9,GNAI2,MYL12A,MEF2D,GNB2,PRKAG2,PIK3CD,ELK1,HSPB1 |
| NF-κB Signaling | 2.54E00 | 1.59E-01 | AZI2,TRD@,PIK3R1,BMPR2,MAP4K4,NGF,EP300,TANK,IKBKB,IKBKG,IL36G,NFKBIA,AKT1,PDGFRA,CSNK2A1,LTBR,PIK3R2,EGFR,PDGFRB,TNFRSF1A,FGFR1,CREBBP,IL36A,FGFR2,NFKB2,IGF2R,DDR1,PIK3CD |
| Ephrin Receptor Signaling | 2.53E00 | 1.45E-01 | PDGFA,MAPK3,MAP4K4,CRK,GRINA,EPHB6,VEGFA,GNB1,SHC1 (includes EG:20416),AKT1,MAP2K2,SDC2,EFNB1,ITGB1,EPHB4,PAK4,PAK2,CFL1,CRKL,ITGA5,RAP1A,PDGFB,FGF1,GNG10,GNAI2,PTPN11,ABI1,WAS,GNB2 |
| Docosahexaenoic Acid (DHA) Signaling | 2.52E00 | 2.24E-01 | BCL2L1,AKT1,BAD,PIK3R1,GSK3A,PIK3CD,PIK3R2,BAX,PNPLA2,APP,BCL2 |
| Regulation of Actin-based Motility by Rho | 2.5E00 | 1.87E-01 | PAK4,MPRIP,PAK2,PFN1,MYL6,CFL1,ACTB,MYLK,MYL9,MYL12A,RHOG,RHOB,WAS,PIP5K1C,RHOT1,ARHGDIA,MYL10 |
| IL-8 Signaling | 2.49E00 | 1.5E-01 | PIK3R1,MAPK3,MAP4K4,CCND1,BCL2,VEGFA,GNB1,IKBKB,IKBKG,RHOG,AKT1,MAP2K2,RHOB,RHOT1,GPLD1,CYBB,PIK3R2,EGFR,PLD3,PAK2,MPO,MMP2,BAX,GNG10,GNAI2,BCL2L1,PRKCI,GNB2,PIK3CD |
| Production of Nitric Oxide and Reactive Oxygen Species in Macrophages | 2.47E00 | 1.44E-01 | MAP3K11,MAPK3,PIK3R1,MAP3K5,IKBKB,IKBKG,NFKBIA,AKT1,JUN,RHOG,RHOB,RHOT1,CYBB,PIK3R2,PTPN6,TNFRSF1A,CREBBP,MPO,PPP1R11,NFKB2,RAP1A,PPP2R1A,PRKCI,PPP2R4,CAT,PIK3CD,SIRPA |
| Cleavage and Polyadenylation of Pre-mRNA | 2.43E00 | 4.17E-01 | PAPOLA,CSTF1,NUDT21,CPSF1,CSTF3 |
| Tight Junction Signaling | 2.4E00 | 1.59E-01 | MYH10,MYL6,CPSF1,MYLK,MPDZ,AKT1,JUN,CLDN4,TGFB1 (includes EG:21803),ARHGEF2,CTNNB1,CSTF1,TNFRSF1A,ACTB,CSDA,NFKB2,MYL9,PPP2R1A,PRKCI,PPP2R4,NUDT21,PRKAG2,MYH9,SPTAN1,CSTF3,CLDN3 |
| ERK/MAPK Signaling | 2.39E00 | 1.47E-01 | BAD,PIK3R1,MAPK3,H3F3A/H3F3B,TLN1,CRK,RAPGEF4,EIF4E,SHC1 (includes EG:20416),MAP2K2,PIK3R2,ITGB1,PAK4,PAK2,YWHAB,CRKL,ITGA5,NFATC1,PPP1R11,RAP1A,ELF2,PPP2R1A,TLN2,PRKCI,DUSP1,PPP2R4,PRKAG2,PIK3CD,ELK1,HSPB1 |
| CREB Signaling in Neurons | 2.39E00 | 1.44E-01 | POLR2D,CALM1 (includes others),PIK3R1,MAPK3,POLR2J,EP300,GNB1,SHC1 (includes EG:20416),POLR2A,AKT1,PLCE1,MAP2K2,PIK3R2,ADCY9,GRM8,ITPR2,CREBBP,ADCY3,GNG10,GNAI2,GRM5,PRKCI,POLR2E,ITPR3,GNB2,PRKAG2,PIK3CD,ELK1,CAMK2G |
| Induction of Apoptosis by HIV1 | 2.38E00 | 1.97E-01 | TP53 (includes EG:22059),TNFRSF1A,BAX,MAP3K5,NFKB2,BCL2,IKBKB,BCL2L1,IKBKG,SLC25A6,NFKBIA,BBC3,FASLG |
| Aryl Hydrocarbon Receptor Signaling | 2.33E00 | 1.51E-01 | TP53 (includes EG:22059),MAPK3,SLC35A2,MDM2,NFKB2,BAX,CCND1,EP300,ALDH3B2,HSP90B1,ALDH1A1,JUN,NCOA2,HSP90AB1,TGFB1 (includes EG:21803),RARB,NFIB,DHFR,NFE2L2,ALDH5A1,CDK2,FASLG,HSPB1,ALDH7A1 |
| IL-15 Signaling | 2.32E00 | 1.94E-01 | STAT6,IL2RG,MAPK3,PIK3R1,NFKB2,BCL2,SHC1 (includes EG:20416),BCL2L1,AKT1,MAP2K2,PIK3CD,PIK3R2,STAT5B |
| Coagulation System | 2.31E00 | 2.37E-01 | KNG1,SERPINC1,PROS1,F5,PLAUR,F13B,FGB (includes EG:110135),TFPI,FGG |
| 4-1BB Signaling in T Lymphocytes | 2.3E00 | 2.35E-01 | IKBKB,IKBKG,JUN,NFKBIA,MAP2K2,MAPK3,MAP3K5,NFKB2 |
| TNFR1 Signaling | 2.29E00 | 2.08E-01 | TANK,CASP6,IKBKB,PAK4,IKBKG,JUN,PAK2,NFKBIA,TNFRSF1A,CASP2,NFKB2 |
| Gα12/13 Signaling | 2.29E00 | 1.64E-01 | MYL6,MAPK3,PIK3R1,MEF2A,ARHGEF1,NFKB2,MAP3K5,MYL9,IKBKB,MYL12A,IKBKG,JUN,NFKBIA,AKT1,MAP2K2,MEF2D,PIK3CD,PIK3R2,MYL10,ELK1,CTNNB1 |
| TGF-β Signaling | 2.27E00 | 1.8E-01 | INHA,MAPK3,CREBBP,SMAD6,BMPR2,PITX2,INHBB,BCL2,EP300,JUN,MAP2K2,TGFB1 (includes EG:21803),RUNX2,MAP4K1,TFE3,ACVR2A |
| Insulin Receptor Signaling | 2.26E00 | 1.64E-01 | SOCS3,FOXO4,EIF2B4,BAD,TRIP10,PIK3R1,MAPK3,CRKL,GSK3A,PPP1R11,CRK,VAMP2,PTPRF,EIF4E,SHC1 (includes EG:20416),AKT1,PRKCI,MAP2K2,PTPN11,FOXO3,PRKAG2,PIK3CD,PIK3R2 |
| Prolactin Signaling | 2.26E00 | 1.88E-01 | SOCS1,SOCS3,MAPK3,PIK3R1,CREBBP,SOCS6,EP300,SHC1 (includes EG:20416),PRKCI,JUN,MAP2K2,PTPN11,PIK3CD,PIK3R2,STAT5B |
| HER-2 Signaling in Breast Cancer | 2.21E00 | 1.85E-01 | TP53 (includes EG:22059),ITGB1,BAD,PIK3R1,MDM2,MMP2,GSK3A,MAP3K5,CCND1,PRKCI,AKT1,PIK3CD,ITGB4,PIK3R2,EGFR |
| PDGF Signaling | 2.21E00 | 1.9E-01 | PDGFA,MAPK3,CRKL,PIK3R1,CRK,PDGFB,SHC1 (includes EG:20416),JUN,MAP2K2,CSNK2A1,PDGFRA,PIK3CD,PIK3R2,ELK1,PDGFRB |
| Activation of IRF by Cytosolic Pattern Recognition Receptors | 2.19E00 | 1.81E-01 | PPIB,CREBBP,NFKB2,IRF3,TANK,IKBKB,IRF7,IKBKG,JUN,NFKBIA,STAT2,PIN1,IFNAR1 |
| ERK5 Signaling | 2.19E00 | 2.03E-01 | IL6ST,LIF,BAD,YWHAE,YWHAB,MEF2A,NGF,AKT1,PTPN11,MEF2D,FOXO3,FOSL1,EGFR |
| Rac Signaling | 2.19E00 | 1.54E-01 | ITGB1,PAK4,MAP3K11,PAK2,CFL1,MAPK3,PIK3R1,ITGA5,NFKB2,IQGAP2,PRKCI,JUN,ARFIP2,MAP2K2,PIP5K1C,CYBB,PIK3CD,PIK3R2,ELK1 |
| EGF Signaling | 2.15E00 | 2.12E-01 | SHC1 (includes EG:20416),JUN,ITPR2,MAPK3,PIK3R1,ITPR3,CSNK2A1,PIK3CD,PIK3R2,ELK1,EGFR |
| Non-Small Cell Lung Cancer Signaling | 2.14E00 | 1.77E-01 | TP53 (includes EG:22059),BAD,ITPR2,MAPK3,PIK3R1,CCND1,AKT1,MAP2K2,RARB,FOXO3,ITPR3,PIK3CD,PIK3R2,EGFR |
| Butanoate Metabolism | 2.12E00 | 1.23E-01 | ACAT2,RDH11,SUCLG2,SDHC,ELOVL6,AADAC,ALDH1A1,ACAT1,SDHD,DBT,EHHADH,PDHB,HMGCS1,ALDH5A1,HADH,ALDH7A1 |
| Aldosterone Signaling in Epithelial Cells | 2.11E00 | 1.53E-01 | CRYAB,HSPA1A/HSPA1B,PIK3R1,MAPK3,DNAJC3,HSPA4,HSP90B1,PLCE1,HSP90AB1,MAP2K2,DNAJC8,PIK3R2,DNAJC22,HSPB6,DNAJC9,ITPR2,HSPA9,DNAJB9,DNAJB14,PRKCI,DUSP1,PIP5K1C,ITPR3,PIK3CD,DNAJB5,HSPB1 |
| Melanoma Signaling | 2.1E00 | 2.17E-01 | TP53 (includes EG:22059),AKT1,BAD,MAP2K2,MAPK3,PIK3R1,MDM2,PIK3CD,PIK3R2,CCND1 |
| FLT3 Signaling in Hematopoietic Progenitor Cells | 2.09E00 | 1.89E-01 | STAT6,BAD,MAPK3,PIK3R1,EIF4E,SHC1 (includes EG:20416),AKT1,MAP2K2,PTPN11,PIK3CD,STAT2,PIK3R2,ELK1,STAT5B |
| T Cell Receptor Signaling | 2.07E00 | 1.65E-01 | CALM1 (includes others),MAPK3,CD4,PIK3R1,NFATC1,NFKB2,PPP3CC,SHB,IKBKB,IKBKG,NFKBIA,NFAT5,JUN,MAP2K2,PPP3R1,PIK3CD,PIK3R2,ELK1 |
| April Mediated Signaling | 2.06E00 | 2.09E-01 | IKBKB,IKBKG,NFAT5,JUN,NFKBIA,TNFSF13,NFATC1,NFKB2,ELK1 |
| 14-3-3-mediated Signaling | 2.05E00 | 1.67E-01 | YWHAE,BAD,TNFRSF1A,YWHAB,TUBB2C,MAPK3,PIK3R1,GSK3A,BAX,MAP3K5,TUBB,TUBB2B,PLCE1,JUN,AKT1,PRKCI,MAP2K2,PIK3CD,PIK3R2,ELK1 |
| Renin-Angiotensin Signaling | 2.05E00 | 1.59E-01 | ADCY9,PTPN6,PAK4,PAK2,ITPR2,MAPK3,PIK3R1,ADCY3,NFKB2,SHC1 (includes EG:20416),PRKCI,JUN,MAP2K2,CCL2,ITPR3,PRKAG2,PIK3CD,PIK3R2,ELK1,ACE |
| LPS-stimulated MAPK Signaling | 2.03E00 | 1.71E-01 | MAPK3,PIK3R1,MAP3K5,NFKB2,IKBKB,IKBKG,PRKCI,JUN,NFKBIA,MAP2K2,PIK3CD,LBP,PIK3R2,ELK1 |
| Thyroid Cancer Signaling | 2.03E00 | 2.17E-01 | TP53 (includes EG:22059),SHC1 (includes EG:20416),TCF4,MAP2K2,MAPK3,NGF,TCF3,CTNNB1,CCND1,TCF7L2 |
| DNA Methylation and Transcriptional Repression Signaling | 2.02E00 | 2.61E-01 | MECP2,DNMT3A,MTA1,CHD4,SAP18,RBBP4 |
| Myc Mediated Apoptosis Signaling | 2.01E00 | 1.97E-01 | TP53 (includes EG:22059),SHC1 (includes EG:20416),AKT1,BAD,YWHAE,YWHAB,PIK3R1,PIK3CD,PIK3R2,BAX,FASLG,BCL2 |
| **Canonical Pathways** | **-log(p-value)** | **Ratio** | **Molecules** |
| PI3K/AKT Signaling | 8.46E00 | 2.5E-01 | BAD,PIK3R1,MAPK3,LIMS1,ILK,GSK3A,MAP3K5,CCND1,EIF4E,BCL2,SHC1 (includes EG:20416),IKBKB,HSP90B1,IKBKG,NFKBIA,AKT1,HSP90AB1,MAP2K2,FOXO3,PIK3R2,CTNNB1,MCL1,TP53 (includes EG:22059),ITGB1,CDC37,RHEB,YWHAE,YWHAB,ITGA5,MDM2,NFKB2,BCL2L1,PPP2R1A,PPP2R4,PIK3CD |
| Molecular Mechanisms of Cancer | 7.93E00 | 1.79E-01 | BAD,MAPK3,PIK3R1,NCSTN,CDKN2C,GSK3A,ARHGEF1,RBL1,CCND1,RHOG,RHOB,MAP2K2,TGFB1 (includes EG:21803),HIPK2,FASLG,ADCY9,TP53 (includes EG:22059),CREBBP,ARHGEF17,NFKB2,AURKA,TCF3,RAP1A,APC,BCL2L1,PTPN11,PIK3CD,FZD5,NOTCH1,CDK2,CAMK2G,TCF4,BMPR2,PSENEN,CRK,FZD1,MAP3K5,E2F3,BCL2,EP300,CASP6,SHC1 (includes EG:20416),NFKBIA,NLK,JUN,AKT1,RHOT1,BBC3,ARHGEF2,PIK3R2,CTNNB1,BMP1,PAK4,PAK2,ADCY3,SMAD6,MDM2,BAX,APH1A,NBN,GNAI2,ARHGEF10,PRKCI,FZD4,NF1 (includes EG:18015),PRKAG2,ELK1,CTNND1 |
| PTEN Signaling | 6.98E00 | 2.42E-01 | FOXO4,BAD,PIK3R1,MAPK3,ILK,BMPR2,GSK3A,CCND1,BCL2,SHC1 (includes EG:20416),IKBKB,IKBKG,AKT1,MAP2K2,FOXO3,CSNK2A1,PDGFRA,PIK3R2,FASLG,PDGFRB,EGFR,ITGB1,FGFR1,ITGA5,FGFR2,NFKB2,IGF2R,DDR1,BCL2L1,PIK3CD |
| Apoptosis Signaling | 6.78E00 | 2.71E-01 | BAD,MAPK3,MAP4K4,MAP3K5,BCL2,ACIN1,CASP6,IKBKB,IKBKG,NFKBIA,MAP2K2,FASLG,MCL1,AIFM1,TP53 (includes EG:22059),TNFRSF1A,LMNA,BAX,NFKB2,CDK1,BCL2L1,CAPNS1,CASP2,CAPN1,CAPN2,SPTAN1 |
| Chronic Myeloid Leukemia Signaling | 5.71E00 | 2.38E-01 | BAD,PIK3R1,MAPK3,HDAC10,CRK,E2F3,RBL1,CCND1,CTBP1,IKBKB,IKBKG,AKT1,MAP2K2,TGFB1 (includes EG:21803),HDAC7,CTBP2,PIK3R2,STAT5B,TP53 (includes EG:22059),CRKL,MDM2,NFKB2,BCL2L1,PTPN11,PIK3CD |
| Integrin Signaling | 5.64E00 | 1.95E-01 | MPRIP,ARHGAP26,MAP3K11,PIK3R1,MAPK3,LIMS1,ILK,CRK,TLN1,MYLK,SHC1 (includes EG:20416),ARF6,AKT1,RHOG,RHOB,MAP2K2,RHOT1,PIK3R2,ITGB4,TSPAN4,ACTN1,ITGB1,PAK4,PAK2,CRKL,ACTB,ITGA5,RAP1A,ARHGAP5,MYL9,MYL12A,TLN2,CAPNS1,ARF3,WAS,CAPN1,ZYX,CAPN2,PIK3CD,ACTN4,ITGA7 |
| Androgen Signaling | 5.49E00 | 1.94E-01 | POLR2D,CALM1 (includes others),MAPK3,POLR2J,CCND1,EP300,GNB1,HSPA4,SHC1 (includes EG:20416),TGFB1I1,POLR2A,JUN,AR,NCOA2,NCOA4,CALR,CREBBP,NFKB2,GNG10,GNAI2,KAT2B,PRKCI,POLR2E,GTF2E1,NCOA1,GNB2,PRKAG2,GTF2H1 |
| Acute Phase Response Signaling | 5.38E00 | 2.02E-01 | IL6ST,SOCS3,SOCS1,TCF4,saa3p,PIK3R1,APOA2,MAPK3,SOCS6,AMBP,CP,MAP3K5,FGG,IKBKB,SHC1 (includes EG:20416),IKBKG,IL36G,NFKBIA,JUN,AKT1,MAP2K2,APCS,FGB (includes EG:110135),LBP,PIK3R2,TTR,TNFRSF1A,AHSG,IL36A,NFKB2,TCF3,ALB,TF,PTPN11,PIK3CD,ELK1 |
| p53 Signaling | 5.26E00 | 2.5E-01 | TP53 (includes EG:22059),GADD45B,PIK3R1,CSNK1D,MDM2,BAX,CCND1,TP53I3,EP300,BCL2,CASP6,BCL2L1,KAT2B,JUN,AKT1,BBC3,STAG1,ADCK3,PIK3CD,PIK3R2,PML,HIPK2,CTNNB1,CDK2 |
| PPAR Signaling | 5E00 | 2.24E-01 | TNFRSF1A,PDGFA,MAPK3,CREBBP,IL36A,MAP4K4,NFKB2,PDGFB,EP300,SHC1 (includes EG:20416),IKBKB,IL36G,IKBKG,HSP90B1,JUN,NFKBIA,MAP2K2,HSP90AB1,PDGFRA,NCOA1,IL1RAPL1,STAT5B,CITED2,PDGFRB |
| Renal Cell Carcinoma Signaling | 5E00 | 2.7E-01 | RBX1,PAK4,PAK2,PIK3R1,MAPK3,CREBBP,CRK,RAP1A,PDGFB,EP300,VEGFA,AKT1,JUN,MAP2K2,PTPN11,CUL2,TGFB1 (includes EG:21803),PIK3CD,PIK3R2,FH |
| Wnt/β-catenin Signaling | 4.85E00 | 2.01E-01 | TCF4,CSNK1G1,SOX15,CSNK1A1,ILK,GSK3A,FZD1,CCND1,EP300,JUN,AKT1,NLK,TGFB1 (includes EG:21803),RARB,CSNK2A1,SFRP5,MAP4K1,CTNNB1,TP53 (includes EG:22059),SOX4,APPL2,CSNK1G3,CREBBP,CSNK1D,MDM2,TCF3,APC,PPP2R1A,FZD4,PPP2R4,NR5A2,FZD5,PIN1,ACVR2A,TCF7L2 |
| Glucocorticoid Receptor Signaling | 4.72E00 | 1.63E-01 | TAF11 (includes EG:309638),POLR2D,HSPA1A/HSPA1B,MAPK3,PIK3R1,POLR2J,TAF13 (includes EG:310784),FGG,BCL2,EP300,HSPA4,SHC1 (includes EG:20416),IKBKB,IKBKG,HSP90B1,NFKBIA,AR,POLR2A,NFAT5,AKT1,JUN,HSP90AB1,NCOA2,CCL2,MAP2K2,TGFB1 (includes EG:21803),BAG1,PPP3R1,FOXO3,PIK3R2,STAT5B,TAF6,HSPA9,CREBBP,NFATC1,PPP3CC,KAT2B,BCL2L1,SUMO1,DUSP1,SMARCA2,POLR2E,GTF2E1,NCOA1,PRKAG2,GTF2H1,PIK3CD,ELK1 |
| PAK Signaling | 4.4E00 | 2.06E-01 | ITGB1,PAK4,PAK2,ARHGAP10,MYL6,CFL1,PDGFA,PIK3R1,MAPK3,PAK1IP1,ITGA5,PDGFB,MYLK,MYL9,SHC1 (includes EG:20416),MYL12A,MAP2K2,PDGFRA,PIK3CD,PIK3R2,MYL10,PDGFRB |
| Prostate Cancer Signaling | 4.37E00 | 2.16E-01 | TP53 (includes EG:22059),BAD,SRD5A1,MAPK3,PIK3R1,CREBBP,MDM2,NFKB2,CCND1,BCL2,HSP90B1,AKT1,NFKBIA,AR,MAP2K2,HSP90AB1,NKX3-1,PIK3CD,PIK3R2,CTNNB1,CDK2 |
| Role of Osteoblasts, Osteoclasts and Chondrocytes in Rheumatoid Arthritis | 4.35E00 | 1.71E-01 | TCF4,CALM1 (includes others),BAD,PIK3R1,MAPK3,CSNK1A1,BMPR2,MAP3K5,FZD1,BCL2,IKBKB,IL36G,IKBKG,NFKBIA,NFAT5,AKT1,JUN,RUNX2,TGFB1 (includes EG:21803),PPP3R1,SFRP5,PIK3R2,CTNNB1,BMP1,ITGB1,TNFRSF1A,IL36A,SMAD6,ITGA5,NFATC1,PPP3CC,TCF3,CSF1R,APC,COL1A1,FZD4,CSF1 (includes EG:12977),FZD5,PIK3CD,IL1RAPL1,TCF7L2 |
| Phospholipase C Signaling | 4.3E00 | 1.65E-01 | MPRIP,TRD@,CALM1 (includes others),MYL6,MAPK3,HDAC10,ARHGEF1,CD79A,EP300,GNB1,SHC1 (includes EG:20416),NFAT5,RHOG,PLCE1,RHOB,MAP2K2,RHOT1,PPP3R1,GPLD1,HDAC7,ARHGEF2,MYL10,ADCY9,ITGB1,PLD3,ITPR2,ADCY3,CREBBP,MEF2A,ITGA5,NFATC1,ARHGEF17,PPP3CC,NFKB2,RAP1A,GNG10,MYL9,MYL12A,PRKCI,ARHGEF10,MEF2D,ITPR3,GNB2 |
| Hepatic Fibrosis / Hepatic Stellate Cell Activation | 4.23E00 | 1.97E-01 | IGFBP4,MYH10,MYL6,PDGFA,BCL2,VEGFA,CCL2,TGFB1 (includes EG:21803),TIMP1,PDGFRA,LBP,FASLG,EGFR,PDGFRB,TNFRSF1A,FGFR1,FGFR2,MMP2,BAX,NFKB2,IFNAR2,PDGFB,FGF1,MYL9,COL1A1,CSF1 (includes EG:12977),MYH9,IL1RAPL1,IFNAR1 |
| Estrogen Receptor Signaling | 4.18E00 | 1.99E-01 | TAF11 (includes EG:309638),POLR2D,MAPK3,POLR2J,H3F3A/H3F3B,TAF13 (includes EG:310784),EP300,CTBP1,SPEN,SHC1 (includes EG:20416),CDK8,POLR2A,MAP2K2,NCOA2,MED15,CTBP2,TAF6,CREBBP,MED6 (includes EG:10001),KAT2B,POLR2E,MED13L,GTF2E1,NCOA1,GTF2H1,MED4 (includes EG:29079),CARM1 |
| Erythropoietin Signaling | 4.08E00 | 2.31E-01 | EPO,SOCS1,SOCS3,PTPN6,EPOR,MAPK3,PIK3R1,NFKB2,SHC1 (includes EG:20416),NFKBIA,PRKCI,AKT1,JUN,MAP2K2,PIK3CD,PIK3R2,STAT5B,ELK1 |
| Huntington's Disease Signaling | 4.01E00 | 1.68E-01 | POLR2D,HSPA1A/HSPA1B,REST,MAPK3,PIK3R1,POLR2J,HDAC10,NGF,EP300,GNB1,HSPA4,CASP6,SHC1 (includes EG:20416),ARFIP2,POLR2A,AKT1,JUN,CPLX2,HDAC7,VAMP3,PIK3R2,NAPA,EGFR,TP53 (includes EG:22059),HSPA9,CREBBP,BAX,GNG10,GRM5,RCOR1,BCL2L1,PRKCI,CAPNS1,POLR2E,CASP2,CAPN1,GNB2,STX16,CAPN2,PIK3CD |
| Glioblastoma Multiforme Signaling | 4E00 | 1.83E-01 | PDGFA,PIK3R1,MAPK3,FZD1,E2F3,CCND1,SHC1 (includes EG:20416),RHOG,AKT1,PLCE1,MAP2K2,RHOB,RHOT1,PDGFRA,PIK3R2,CTNNB1,EGFR,PDGFRB,TP53 (includes EG:22059),ITPR2,MDM2,TCF3,PDGFB,APC,FZD4,NF1 (includes EG:18015),ITPR3,FZD5,PIK3CD,CDK2 |
| NRF2-mediated Oxidative Stress Response | 3.99E00 | 1.77E-01 | AKR7A2,RBX1,PPIB,PIK3R1,MAPK3,DNAJC3,MAP3K5,SOD3,EP300,JUN,AKT1,MAP2K2,DNAJC8,PMF1,FOSL1,DNAJA2,JUND,TXN (includes EG:116484),PIK3R2,NFE2L2,DNAJC9,ACTB,CREBBP,SLC35A2,JUNB,DNAJB9,DNAJB14,PRKCI,STIP1,CAT,PIK3CD,PTPLAD1,DNAJB5,EPHX1 |
| FAK Signaling | 3.99E00 | 2.06E-01 | ITGB1,PAK4,PAK2,ARHGAP26,ACTB,PIK3R1,MAPK3,ITGA5,CRK,TLN1,CAPNS1,TLN2,AKT1,MAP2K2,WAS,CAPN1,CAPN2,PIK3CD,PIK3R2,TNS1,EGFR |
| Reelin Signaling in Neurons | 3.85E00 | 2.32E-01 | ITGB1,MAP3K11,PIK3R1,CRKL,MAPK8IP2,ITGA5,ARHGEF1,RELN,MAPK8IP3,FRK,APP,APBB1,YES1,AKT1,ARHGEF10,MAP4K1,ARHGEF2,PIK3CD,PIK3R2 |
| Hereditary Breast Cancer Signaling | 3.83E00 | 1.94E-01 | POLR2D,GADD45B,PIK3R1,POLR2J,HDAC10,DDB2,CCND1,RAD50,EP300,POLR2A,AKT1,HDAC7,RFC2,PIK3R2,TP53 (includes EG:22059),CREBBP,RFC5,CDK1,NBN,PALB2,SMARCA2,POLR2E,H2AFX,PIK3CD,RFC3 |
| JAK/Stat Signaling | 3.8E00 | 2.5E-01 | STAT6,SOCS1,SOCS3,PTPN6,PIAS2,MAPK3,PIK3R1,SOCS6,SHC1 (includes EG:20416),AKT1,PTPN11,MAP2K2,STAT2,PIK3CD,PIK3R2,STAT5B |
| Neuregulin Signaling | 3.77E00 | 2.06E-01 | ITGB1,BAD,PIK3R1,MAPK3,CRKL,ITGA5,CRK,SHC1 (includes EG:20416),ERBB2IP,HSP90B1,PRKCI,AKT1,PICK1,MAP2K2,PTPN11,HSP90AB1,PIK3R2,STAT5B,ELK1,EGFR,MATK |
| Clathrin-mediated Endocytosis Signaling | 3.75E00 | 1.8E-01 | RAB4A,PDGFA,PIK3R1,GAK,VEGFA,CD2AP,ARF6,AP1G2,RAB11B,PPP3R1,CSNK2A1,PIK3R2,SH3GLB2,ITGB4,HGS,ITGB1,MYO6,AP2M1,EPN1,ACTB,ITGA5,MDM2,PPP3CC,PDGFB,FGF1,FGF21,ARRB2,TF,PIP5K1C,CLTA,PIK3CD |
| Glioma Signaling | 3.75E00 | 1.96E-01 | TP53 (includes EG:22059),CALM1 (includes others),PDGFA,PIK3R1,MAPK3,CDKN2C,MDM2,E2F3,RBL1,CCND1,IGF2R,PDGFB,SHC1 (includes EG:20416),AKT1,PRKCI,MAP2K2,PDGFRA,PIK3CD,PIK3R2,EGFR,PDGFRB,CAMK2G |
| IL-6 Signaling | 3.7E00 | 2.1E-01 | IL6ST,SOCS1,TNFRSF1A,MAPK3,IL36A,MAP4K4,NFKB2,COL1A1,SHC1 (includes EG:20416),IKBKB,IKBKG,IL36G,JUN,NFKBIA,MAP2K2,PTPN11,CSNK2A1,IL1RAPL1,LBP,ELK1,HSPB1 |
| Role of NFAT in Regulation of the Immune Response | 3.68E00 | 1.65E-01 | CSNK1G1,CALM1 (includes others),TRD@,PIK3R1,CD4,MAPK3,CSNK1A1,GSK3A,CD79A,CABIN1,GNB1,IKBKB,IKBKG,NFKBIA,JUN,AKT1,NFAT5,MAP2K2,PPP3R1,PIK3R2,ITPR2,CSNK1G3,CSNK1D,MEF2A,NFATC1,PPP3CC,NFKB2,GNG10,GNAI2,MEF2D,ITPR3,GNB2,PIK3CD |
| IGF-1 Signaling | 3.68E00 | 2.06E-01 | IGFBP4,SOCS1,SOCS3,YWHAE,BAD,YWHAB,MAPK3,PIK3R1,SOCS6,SHC1 (includes EG:20416),PRKCI,AKT1,JUN,MAP2K2,PTPN11,FOXO3,CSNK2A1,PRKAG2,PIK3CD,PIK3R2,ELK1,CYR61 |
| SAPK/JNK Signaling | 3.63E00 | 2.06E-01 | TP53 (includes EG:22059),MAP3K11,DUSP8,TRD@,PIK3R1,CRKL,MAPK8IP2,MAP4K4,CRK,NFATC1,MAP3K5,MAPK8IP3,GNB1,SHC1 (includes EG:20416),MINK1,JUN,DUSP10,MAP4K1,PIK3CD,PIK3R2,ELK1 |
| Germ Cell-Sertoli Cell Junction Signaling | 3.49E00 | 1.8E-01 | MAP3K11,PIK3R1,MAPK3,ILK,MAP3K5,TUBB,TUBB2B,AGGF1,AKT1,RHOG,MAP2K2,RHOB,RHOT1,TGFB1 (includes EG:21803),MTMR2,PIK3R2,JUP,CTNNB1,ACTN1,ITGB1,EPN1,PAK4,PAK2,TUBB2C,TNFRSF1A,ACTB,ZYX,PIK3CD,ACTN4,CTNND1 |
| IL-3 Signaling | 3.48E00 | 2.3E-01 | STAT6,PTPN6,BAD,MAPK3,CRKL,PIK3R1,PPP3CC,SHC1 (includes EG:20416),PRKCI,AKT1,JUN,MAP2K2,PPP3R1,PIK3CD,PIK3R2,STAT5B,ELK1 |
| RAR Activation | 3.43E00 | 1.71E-01 | PIK3R1,ADH1C (includes EG:11522),MAP3K5,EP300,VEGFA,ALDH1A1,AKT1,JUN,TGFB1 (includes EG:21803),RARB,CSNK2A1,SORBS3,PIK3R2,STAT5B,CITED2,ADCY9,RDH14,RDH11,CREBBP,ADCY3,SMAD6,NFKB2,KAT2B,PRKCI,SMARCA2,DUSP1,NCOA1,PRKAG2,GTF2H1,PIK3CD,PML,CARM1 |
| B Cell Receptor Signaling | 3.36E00 | 1.79E-01 | MAP3K11,BAD,CALM1 (includes others),PIK3R1,MAPK3,GSK3A,MAP3K5,CD79A,SHC1 (includes EG:20416),IKBKB,IKBKG,AKT1,JUN,NFAT5,NFKBIA,MAP2K2,PPP3R1,CD22,PIK3R2,PTPN6,NFATC1,PPP3CC,NFKB2,BCL2L1,PTPN11,PIK3CD,ELK1,CAMK2G |
| Hypoxia Signaling in the Cardiovascular System | 3.3E00 | 2.35E-01 | TP53 (includes EG:22059),EPO,CSNK1D,MDM2,UBE2D1,EP300,VEGFA,HSP90B1,AKT1,JUN,NFKBIA,SUMO1,HSP90AB1,UBE2B,UBE2G1,UBE2J1 |
| IL-2 Signaling | 3.25E00 | 2.41E-01 | SOCS1,IL2RG,MAPK3,PIK3R1,SHC1 (includes EG:20416),AKT1,JUN,MAP2K2,PTPN11,CSNK2A1,PIK3CD,PIK3R2,ELK1,STAT5B |
| Pancreatic Adenocarcinoma Signaling | 3.25E00 | 1.85E-01 | TP53 (includes EG:22059),PLD3,BAD,PIK3R1,MAPK3,MDM2,NFKB2,E2F3,CCND1,BCL2,VEGFA,BCL2L1,AKT1,MAP2K2,TGFB1 (includes EG:21803),GPLD1,PIK3CD,PIK3R2,ELK1,NOTCH1,CDK2,EGFR |
| Thrombin Signaling | 3.23E00 | 1.64E-01 | MPRIP,MYL6,PIK3R1,MAPK3,ARHGEF1,MYLK,GNB1,SHC1 (includes EG:20416),IKBKB,RHOG,AKT1,PLCE1,RHOB,MAP2K2,RHOT1,ARHGEF2,PIK3R2,MYL10,EGFR,ADCY9,ITPR2,ADCY3,NFKB2,GNG10,MYL9,GNAI2,MYL12A,ARHGEF10,PRKCI,ITPR3,GNB2,PIK3CD,ELK1,CAMK2G |
| Extrinsic Prothrombin Activation Pathway | 3.2E00 | 3.5E-01 | SERPINC1,PROS1,F5,F13B,FGB (includes EG:110135),TFPI,FGG |
| Protein Kinase A Signaling | 3.17E00 | 1.49E-01 | MYH10,BAD,MYL6,MAPK3,GSK3A,MYLK,GNB1,PLCE1,MAP2K2,TGFB1 (includes EG:21803),PPP3R1,MYL10,ADCY9,YWHAE,YWHAB,ITPR2,PDE9A,CREBBP,PPP1R11,NFKB2,PPP3CC,TCF3,RAP1A,GNG10,MYL9,AKAP13,MYL12A,ITPR3,GNB2,CAMK2G,ANAPC2,TCF4,CALM1 (includes others),H3F3A/H3F3B,AKAP7,NFAT5,NFKBIA,FLNA,CTNNB1,ADCY3,PYGL,NFATC1,GNAI2,PRKCI,PRKAG2,ADD1,ELK1,TCF7L2,CDC27 |
| GM-CSF Signaling | 3.11E00 | 2.24E-01 | MAPK3,PIK3R1,PPP3CC,CCND1,SHC1 (includes EG:20416),BCL2L1,AKT1,MAP2K2,PTPN11,PPP3R1,PIK3CD,PIK3R2,ELK1,STAT5B,CAMK2G |
| Role of CHK Proteins in Cell Cycle Checkpoint Control | 3.1E00 | 2.86E-01 | TP53 (includes EG:22059),RAD17 (includes EG:19356),RFC2,RFC5,E2F3,CDK1,RAD50,CDK2,RFC3,NBN |
| Role of Tissue Factor in Cancer | 3.08E00 | 1.93E-01 | TP53 (includes EG:22059),ITGB1,CFL1,PIK3R1,MAPK3,PLAUR,FRK,EIF4E,FGG,VEGFA,BCL2L1,YES1,ARRB2,AKT1,PTPN11,CSF1 (includes EG:12977),FGB (includes EG:110135),PIK3CD,PIK3R2,STAT5B,CYR61,EGFR |
| Actin Cytoskeleton Signaling | 3.05E00 | 1.51E-01 | MYH10,PFN1,MPRIP,MYL6,PDGFA,PIK3R1,MAPK3,ARHGEF1,CRK,MYLK,SHC1 (includes EG:20416),IQGAP2,MAP2K2,LBP,PIK3R2,MYL10,ACTN1,MATK,ITGB1,PAK4,PAK2,CFL1,ACTB,CRKL,ITGA5,PDGFB,APC,FGF1,MYL9,FGF21,MYL12A,PIP5K1C,WAS,MYH9,PIK3CD,ACTN4 |
| Protein Ubiquitination Pathway | 3.04E00 | 1.53E-01 | PSMB3,B2M,ANAPC2,USP24,RBX1,CRYAB,USP5,HSPA1A/HSPA1B,UBE3B,UBR2 (includes EG:224826),DNAJC3,HSPA4,HSP90B1,HSP90AB1,UBE2B,BAG1,PSMC6,DNAJC8,UCHL5,NEDD4L,PSMA2,DNAJC22,UBE2J1,AMFR,HSPB6,DNAJC9,USP15,HSPA9,MDM2,DNAJB9,USP33,DNAJB14,UBE2D1,FZR1,CUL2,UBE2G1,PSMA4,USP46,UBA1,USP25,DNAJB5,HSPB1 |
| Polyamine Regulation in Colon Cancer | 3.02E00 | 2.76E-01 | TCF4,AZIN1,PSMF1,SAT1,PSME4,CTNNB1,PSME3,APC |
| ILK Signaling | 3.01E00 | 1.66E-01 | MYH10,MYL6,PIK3R1,MAPK3,ILK,GSK3A,CCND1,VEGFA,TGFB1I1,RHOG,AKT1,JUN,RHOB,FLNA,RHOT1,PIK3R2,ITGB4,CTNNB1,ACTN1,ITGB1,CFL1,TNFRSF1A,ACTB,FERMT2,SNAI1,NFKB2,MYL9,PPP2R1A,PPP2R4,MYH9,PIK3CD,ACTN4 |
| ATM Signaling | 2.98E00 | 2.41E-01 | TP53 (includes EG:22059),SMC3,GADD45B,MDM2,CDK1,RAD50,SMC1A,NBN,JUN,NFKBIA,SMC2,H2AFX,CDK2 |
| Amyloid Processing | 2.98E00 | 2.32E-01 | MAPK3,CSNK1D,CSNK1A1,NCSTN,PSENEN,APP,APH1A,AKT1,CAPNS1,CAPN1,CSNK2A1,PRKAG2,CAPN2 |
| Role of NFAT in Cardiac Hypertrophy | 2.97E00 | 1.54E-01 | IL6ST,LIF,CALM1 (includes others),PIK3R1,MAPK3,CSNK1A1,HDAC10,EP300,CABIN1,GNB1,SHC1 (includes EG:20416),AKT1,PLCE1,MAP2K2,TGFB1 (includes EG:21803),PPP3R1,HDAC7,PIK3R2,ADCY9,ITPR2,ADCY3,MEF2A,PPP3CC,GNG10,GNAI2,PRKCI,MEF2D,ITPR3,GNB2,PRKAG2,PIK3CD,CAMK2G |
| TR/RXR Activation | 2.96E00 | 1.98E-01 | AKR1C1/AKR1C2,GPS2,PIK3R1,BCL3,MDM2,ME1,EP300,KLF9,SLC16A3,UCP3,AKT1,NCOA2,SREBF2,DIO1,NCOA1,NCOA4,PIK3CD,PIK3R2,SYT12 |
| Colorectal Cancer Metastasis Signaling | 2.94E00 | 1.51E-01 | IL6ST,TCF4,BAD,PIK3R1,MAPK3,FZD1,CCND1,VEGFA,GNB1,RHOG,AKT1,JUN,RHOB,MMP25,MAP2K2,RHOT1,TGFB1 (includes EG:21803),PIK3R2,CTNNB1,EGFR,MMP19,TP53 (includes EG:22059),ADCY9,TNFRSF1A,ADRBK2,ADCY3,MMP2,BAX,NFKB2,TCF3,APC,GNG10,BCL2L1,FZD4,GNB2,PRKAG2,FZD5,PIK3CD,TCF7L2 |
| Death Receptor Signaling | 2.92E00 | 2.15E-01 | TNFRSF1A,MAP4K4,MAP3K5,NFKB2,BCL2,TANK,CASP6,IKBKB,IKBKG,NFKBIA,TNFSF12,CASP2,FASLG,HSPB1 |
| VEGF Signaling | 2.9E00 | 1.92E-01 | EIF2S3,EIF2B4,YWHAE,BAD,ACTB,MAPK3,PIK3R1,EIF1,BCL2,VEGFA,SHC1 (includes EG:20416),BCL2L1,AKT1,MAP2K2,FOXO3,PIK3CD,PIK3R2,ACTN4,ACTN1 |
| Role of JAK2 in Hormone-like Cytokine Signaling | 2.88E00 | 2.7E-01 | EPO,SHC1 (includes EG:20416),SOCS1,SOCS3,PTPN6,PTPN11,EPOR,SOCS6,STAT5B,SIRPA |
| fMLP Signaling in Neutrophils | 2.87E00 | 1.72E-01 | CALM1 (includes others),ITPR2,MAPK3,PIK3R1,NFATC1,NFKB2,PPP3CC,GNG10,GNB1,GNAI2,NFKBIA,NFAT5,PRKCI,MAP2K2,WAS,PPP3R1,ITPR3,GNB2,CYBB,PIK3CD,PIK3R2,ELK1 |
| Angiopoietin Signaling | 2.82E00 | 2.03E-01 | PAK4,PAK2,GRB14,BAD,PIK3R1,CRK,NFKB2,IKBKB,IKBKG,NFKBIA,AKT1,PTPN11,PIK3CD,PIK3R2,STAT5B |
| Endometrial Cancer Signaling | 2.82E00 | 2.28E-01 | TP53 (includes EG:22059),BAD,MAPK3,PIK3R1,ILK,CCND1,AKT1,MAP2K2,FOXO3,PIK3CD,PIK3R2,ELK1,CTNNB1 |
| CXCR4 Signaling | 2.81E00 | 1.66E-01 | MYL6,PIK3R1,MAPK3,CD4,CRK,GNB1,RHOG,AKT1,JUN,MAP2K2,RHOB,RHOT1,PIK3R2,MYL10,ADCY9,PAK4,PAK2,ITPR2,ADCY3,GNG10,GNAI2,MYL9,MYL12A,PRKCI,ITPR3,GNB2,PIK3CD,ELK1 |
| Breast Cancer Regulation by Stathmin1 | 2.81E00 | 1.57E-01 | CALM1 (includes others),PIK3R1,MAPK3,ARHGEF1,E2F3,TUBB,TUBB2B,GNB1,SHC1 (includes EG:20416),MAP2K2,RB1CC1,ARHGEF2,PIK3R2,TP53 (includes EG:22059),ADCY9,TUBB2C,ITPR2,ADCY3,ARHGEF17,PPP1R11,CDK1,GNG10,GNAI2,PPP2R1A,ARHGEF10,PRKCI,PPP2R4,ITPR3,GNB2,PRKAG2,PIK3CD,CDK2,CAMK2G |
| Axonal Guidance Signaling | 2.8E00 | 1.32E-01 | GLI2,PFN1,MYL6,MAPK3,PIK3R1,TUBB,VEGFA,GNB1,MAP2K2,PPP3R1,PLXNB2,MYL10,CFL1,CRKL,ITGA5,PPP3CC,RAP1A,PDGFB,GNG10,MYL9,MYL12A,PTPN11,RTN4,GNB2,PIK3CD,FZD5,PDGFA,SEMA6A,CRK,FZD1,NGF,EIF4E,TUBB2B,EPHB6,SHC1 (includes EG:20416),AKT1,NFAT5,PLXNA1,SDC2,EFNB1,PIK3R2,SHANK2,BMP1,ITGB1,EPHB4,PAK4,PAK2,NRP2,ADAM2,TUBB2C,NFATC1,PLXND1,GNAI2,PRKCI,FZD4,WAS,PRKAG2 |
| RANK Signaling in Osteoclasts | 2.79E00 | 1.89E-01 | MAP3K11,CALM1 (includes others),MAPK3,PIK3R1,NFATC1,PPP3CC,MAP3K5,NFKB2,IKBKB,IKBKG,NFKBIA,AKT1,JUN,MAP2K2,PPP3R1,PIK3CD,PIK3R2,ELK1 |
| Macropinocytosis Signaling | 2.75E00 | 1.97E-01 | ITGB1,PDGFA,PIK3R1,ITGA5,NGF,CSF1R,PDGFB,ARF6,PRKCI,ABI1,CSF1 (includes EG:12977),PIK3CD,ITGB4,PIK3R2,ACTN4 |
| Role of Macrophages, Fibroblasts and Endothelial Cells in Rheumatoid Arthritis | 2.73E00 | 1.38E-01 | IL6ST,SOCS1,SOCS3,TCF4,CALM1 (includes others),PDGFA,MAPK3,PIK3R1,IL32,CSNK1A1,FZD1,CCND1,VEGFA,IKBKB,IKBKG,IL36G,C5AR1,NFKBIA,NLK,NFAT5,AKT1,JUN,PLCE1,CCL2,MAP2K2,TGFB1 (includes EG:21803),PPP3R1,SFRP5,PIK3R2,LTBR,CTNNB1,TNFRSF1A,IL36A,NFATC1,PPP3CC,TCF3,PDGFB,APC,PRKCI,FZD4,CSF1 (includes EG:12977),FZD5,PIK3CD,IL1RAPL1,TCF7L2,CAMK2G |
| iCOS-iCOSL Signaling in T Helper Cells | 2.7E00 | 1.72E-01 | IL2RG,BAD,CALM1 (includes others),TRD@,ITPR2,CD4,PIK3R1,NFATC1,NFKB2,PPP3CC,SHC1 (includes EG:20416),IKBKB,IKBKG,AKT1,NFAT5,NFKBIA,PPP3R1,ITPR3,PIK3CD,PIK3R2,CAMK2G |
| Intrinsic Prothrombin Activation Pathway | 2.7E00 | 2.65E-01 | COL1A1,KNG1,KLK1,SERPINC1,PROS1,F5,F13B,FGB (includes EG:110135),FGG |
| Lymphotoxin β Receptor Signaling | 2.66E00 | 2.13E-01 | MAPK3,PIK3R1,CREBBP,NFKB2,EP300,IKBKB,BCL2L1,IKBKG,AKT1,NFKBIA,PIK3CD,LTBR,PIK3R2 |
| HIF1α Signaling | 2.64E00 | 1.85E-01 | TP53 (includes EG:22059),EPO,EGLN2,RBX1,MAPK3,PIK3R1,CREBBP,MMP2,MDM2,EP300,P4HTM,VEGFA,JUN,AKT1,MMP25,CUL2,NCOA1,PIK3CD,PIK3R2,MMP19 |
| Acute Myeloid Leukemia Signaling | 2.61E00 | 1.95E-01 | TCF4,BAD,MAPK3,PIK3R1,NFKB2,TCF3,CCND1,CSF1R,AKT1,MAP2K2,PIK3CD,JUP,PIK3R2,PML,STAT5B,TCF7L2 |
| Lysine Degradation | 2.61E00 | 1.22E-01 | SETD8,AASDHPPT,ACAT2,RDH11,ELOVL6,EP300,KAT2B,ALDH1A1,SMYD3,EHMT2,ACAT1,DBT,EHHADH,SHMT2,PLOD3,HADH,ALDH7A1 |
| PPARα/RXRα Activation | 2.61E00 | 1.56E-01 | CYP2C9,MAPK3,APOA2,MAP4K4,ADIPOR1,ABCA1,EP300,SHC1 (includes EG:20416),IKBKB,IKBKG,HSP90B1,JUN,PLCE1,NFKBIA,HSP90AB1,MAP2K2,TGFB1 (includes EG:21803),CLOCK,STAT5B,ADCY9,ACOX1,CREBBP,ADCY3,CD36,NFKB2,CAND1,PRKAG2,IL1RAPL1,ACVR2A |
| Calcium Signaling | 2.6E00 | 1.45E-01 | MYH10,MYL6,CALM1 (includes others),ATP2B1,MAPK3,HDAC10,EP300,GRINA,CABIN1,NFAT5,TRPV6,PPP3R1,HDAC7,ASPH,TPM4,CALR,ATP2C1,ITPR2,CREBBP,MEF2A,NFATC1,PPP3CC,RAP1A,ATP2B2,MYL9,MEF2D,ITPR3,PRKAG2,MYH9,CAMK2G |
| PI3K Signaling in B Lymphocytes | 2.58E00 | 1.68E-01 | CALM1 (includes others),ITPR2,MAPK3,PIK3R1,NFATC1,NFKB2,PPP3CC,CD79A,IKBKB,IKBKG,PLCE1,JUN,AKT1,NFKBIA,NFAT5,PRKCI,MAP2K2,PPP3R1,FOXO3,ITPR3,PIK3CD,PIK3R2,ELK1,CAMK2G |
| CD28 Signaling in T Helper Cells | 2.58E00 | 1.67E-01 | PTPN6,CALM1 (includes others),TRD@,ITPR2,CD4,PIK3R1,NFATC1,NFKB2,PPP3CC,IKBKB,IKBKG,JUN,AKT1,NFKBIA,NFAT5,MAP2K2,PTPN11,WAS,PPP3R1,ITPR3,PIK3CD,PIK3R2 |
| Role of Wnt/GSK-3β Signaling in the Pathogenesis of Influenza | 2.55E00 | 1.98E-01 | TCF4,CSNK1G1,CSNK1G3,CSNK1A1,CSNK1D,FZD1,TCF3,APC,FZD4,NCOA2,NCOA1,NCOA4,FZD5,CTNNB1,TCF7L2,IFNAR1 |
| Human Embryonic Stem Cell Pluripotency | 2.54E00 | 1.56E-01 | TCF4,PDGFA,PIK3R1,FGFR1,SMAD6,FGFR2,BMPR2,GSK3A,FZD1,NGF,TCF3,APC,PDGFB,AKT1,FZD4,TGFB1 (includes EG:21803),PDGFRA,FZD5,PIK3CD,PIK3R2,CTNNB1,TCF7L2,BMP1,PDGFRB |
| Cardiac Hypertrophy Signaling | 2.54E00 | 1.47E-01 | EIF2B4,MAP3K11,CALM1 (includes others),MYL6,PIK3R1,MAPK3,MAP3K5,EIF4E,EP300,GNB1,AKT1,JUN,PLCE1,RHOG,RHOB,MAP2K2,RHOT1,TGFB1 (includes EG:21803),PPP3R1,PIK3R2,MYL10,ADCY9,CREBBP,ADCY3,MEF2A,PPP3CC,GNG10,MYL9,GNAI2,MYL12A,MEF2D,GNB2,PRKAG2,PIK3CD,ELK1,HSPB1 |
| NF-κB Signaling | 2.54E00 | 1.59E-01 | AZI2,TRD@,PIK3R1,BMPR2,MAP4K4,NGF,EP300,TANK,IKBKB,IKBKG,IL36G,NFKBIA,AKT1,PDGFRA,CSNK2A1,LTBR,PIK3R2,EGFR,PDGFRB,TNFRSF1A,FGFR1,CREBBP,IL36A,FGFR2,NFKB2,IGF2R,DDR1,PIK3CD |
| Ephrin Receptor Signaling | 2.53E00 | 1.45E-01 | PDGFA,MAPK3,MAP4K4,CRK,GRINA,EPHB6,VEGFA,GNB1,SHC1 (includes EG:20416),AKT1,MAP2K2,SDC2,EFNB1,ITGB1,EPHB4,PAK4,PAK2,CFL1,CRKL,ITGA5,RAP1A,PDGFB,FGF1,GNG10,GNAI2,PTPN11,ABI1,WAS,GNB2 |
| Docosahexaenoic Acid (DHA) Signaling | 2.52E00 | 2.24E-01 | BCL2L1,AKT1,BAD,PIK3R1,GSK3A,PIK3CD,PIK3R2,BAX,PNPLA2,APP,BCL2 |
| Regulation of Actin-based Motility by Rho | 2.5E00 | 1.87E-01 | PAK4,MPRIP,PAK2,PFN1,MYL6,CFL1,ACTB,MYLK,MYL9,MYL12A,RHOG,RHOB,WAS,PIP5K1C,RHOT1,ARHGDIA,MYL10 |
| IL-8 Signaling | 2.49E00 | 1.5E-01 | PIK3R1,MAPK3,MAP4K4,CCND1,BCL2,VEGFA,GNB1,IKBKB,IKBKG,RHOG,AKT1,MAP2K2,RHOB,RHOT1,GPLD1,CYBB,PIK3R2,EGFR,PLD3,PAK2,MPO,MMP2,BAX,GNG10,GNAI2,BCL2L1,PRKCI,GNB2,PIK3CD |
| Production of Nitric Oxide and Reactive Oxygen Species in Macrophages | 2.47E00 | 1.44E-01 | MAP3K11,MAPK3,PIK3R1,MAP3K5,IKBKB,IKBKG,NFKBIA,AKT1,JUN,RHOG,RHOB,RHOT1,CYBB,PIK3R2,PTPN6,TNFRSF1A,CREBBP,MPO,PPP1R11,NFKB2,RAP1A,PPP2R1A,PRKCI,PPP2R4,CAT,PIK3CD,SIRPA |
| Cleavage and Polyadenylation of Pre-mRNA | 2.43E00 | 4.17E-01 | PAPOLA,CSTF1,NUDT21,CPSF1,CSTF3 |
| Tight Junction Signaling | 2.4E00 | 1.59E-01 | MYH10,MYL6,CPSF1,MYLK,MPDZ,AKT1,JUN,CLDN4,TGFB1 (includes EG:21803),ARHGEF2,CTNNB1,CSTF1,TNFRSF1A,ACTB,CSDA,NFKB2,MYL9,PPP2R1A,PRKCI,PPP2R4,NUDT21,PRKAG2,MYH9,SPTAN1,CSTF3,CLDN3 |
| ERK/MAPK Signaling | 2.39E00 | 1.47E-01 | BAD,PIK3R1,MAPK3,H3F3A/H3F3B,TLN1,CRK,RAPGEF4,EIF4E,SHC1 (includes EG:20416),MAP2K2,PIK3R2,ITGB1,PAK4,PAK2,YWHAB,CRKL,ITGA5,NFATC1,PPP1R11,RAP1A,ELF2,PPP2R1A,TLN2,PRKCI,DUSP1,PPP2R4,PRKAG2,PIK3CD,ELK1,HSPB1 |
| CREB Signaling in Neurons | 2.39E00 | 1.44E-01 | POLR2D,CALM1 (includes others),PIK3R1,MAPK3,POLR2J,EP300,GNB1,SHC1 (includes EG:20416),POLR2A,AKT1,PLCE1,MAP2K2,PIK3R2,ADCY9,GRM8,ITPR2,CREBBP,ADCY3,GNG10,GNAI2,GRM5,PRKCI,POLR2E,ITPR3,GNB2,PRKAG2,PIK3CD,ELK1,CAMK2G |
| Induction of Apoptosis by HIV1 | 2.38E00 | 1.97E-01 | TP53 (includes EG:22059),TNFRSF1A,BAX,MAP3K5,NFKB2,BCL2,IKBKB,BCL2L1,IKBKG,SLC25A6,NFKBIA,BBC3,FASLG |
| Aryl Hydrocarbon Receptor Signaling | 2.33E00 | 1.51E-01 | TP53 (includes EG:22059),MAPK3,SLC35A2,MDM2,NFKB2,BAX,CCND1,EP300,ALDH3B2,HSP90B1,ALDH1A1,JUN,NCOA2,HSP90AB1,TGFB1 (includes EG:21803),RARB,NFIB,DHFR,NFE2L2,ALDH5A1,CDK2,FASLG,HSPB1,ALDH7A1 |
| IL-15 Signaling | 2.32E00 | 1.94E-01 | STAT6,IL2RG,MAPK3,PIK3R1,NFKB2,BCL2,SHC1 (includes EG:20416),BCL2L1,AKT1,MAP2K2,PIK3CD,PIK3R2,STAT5B |
| Coagulation System | 2.31E00 | 2.37E-01 | KNG1,SERPINC1,PROS1,F5,PLAUR,F13B,FGB (includes EG:110135),TFPI,FGG |
| 4-1BB Signaling in T Lymphocytes | 2.3E00 | 2.35E-01 | IKBKB,IKBKG,JUN,NFKBIA,MAP2K2,MAPK3,MAP3K5,NFKB2 |
| TNFR1 Signaling | 2.29E00 | 2.08E-01 | TANK,CASP6,IKBKB,PAK4,IKBKG,JUN,PAK2,NFKBIA,TNFRSF1A,CASP2,NFKB2 |
| Gα12/13 Signaling | 2.29E00 | 1.64E-01 | MYL6,MAPK3,PIK3R1,MEF2A,ARHGEF1,NFKB2,MAP3K5,MYL9,IKBKB,MYL12A,IKBKG,JUN,NFKBIA,AKT1,MAP2K2,MEF2D,PIK3CD,PIK3R2,MYL10,ELK1,CTNNB1 |
| TGF-β Signaling | 2.27E00 | 1.8E-01 | INHA,MAPK3,CREBBP,SMAD6,BMPR2,PITX2,INHBB,BCL2,EP300,JUN,MAP2K2,TGFB1 (includes EG:21803),RUNX2,MAP4K1,TFE3,ACVR2A |
| Insulin Receptor Signaling | 2.26E00 | 1.64E-01 | SOCS3,FOXO4,EIF2B4,BAD,TRIP10,PIK3R1,MAPK3,CRKL,GSK3A,PPP1R11,CRK,VAMP2,PTPRF,EIF4E,SHC1 (includes EG:20416),AKT1,PRKCI,MAP2K2,PTPN11,FOXO3,PRKAG2,PIK3CD,PIK3R2 |
| Prolactin Signaling | 2.26E00 | 1.88E-01 | SOCS1,SOCS3,MAPK3,PIK3R1,CREBBP,SOCS6,EP300,SHC1 (includes EG:20416),PRKCI,JUN,MAP2K2,PTPN11,PIK3CD,PIK3R2,STAT5B |
| HER-2 Signaling in Breast Cancer | 2.21E00 | 1.85E-01 | TP53 (includes EG:22059),ITGB1,BAD,PIK3R1,MDM2,MMP2,GSK3A,MAP3K5,CCND1,PRKCI,AKT1,PIK3CD,ITGB4,PIK3R2,EGFR |
| PDGF Signaling | 2.21E00 | 1.9E-01 | PDGFA,MAPK3,CRKL,PIK3R1,CRK,PDGFB,SHC1 (includes EG:20416),JUN,MAP2K2,CSNK2A1,PDGFRA,PIK3CD,PIK3R2,ELK1,PDGFRB |
| Activation of IRF by Cytosolic Pattern Recognition Receptors | 2.19E00 | 1.81E-01 | PPIB,CREBBP,NFKB2,IRF3,TANK,IKBKB,IRF7,IKBKG,JUN,NFKBIA,STAT2,PIN1,IFNAR1 |
| ERK5 Signaling | 2.19E00 | 2.03E-01 | IL6ST,LIF,BAD,YWHAE,YWHAB,MEF2A,NGF,AKT1,PTPN11,MEF2D,FOXO3,FOSL1,EGFR |
| Rac Signaling | 2.19E00 | 1.54E-01 | ITGB1,PAK4,MAP3K11,PAK2,CFL1,MAPK3,PIK3R1,ITGA5,NFKB2,IQGAP2,PRKCI,JUN,ARFIP2,MAP2K2,PIP5K1C,CYBB,PIK3CD,PIK3R2,ELK1 |
| EGF Signaling | 2.15E00 | 2.12E-01 | SHC1 (includes EG:20416),JUN,ITPR2,MAPK3,PIK3R1,ITPR3,CSNK2A1,PIK3CD,PIK3R2,ELK1,EGFR |
| Non-Small Cell Lung Cancer Signaling | 2.14E00 | 1.77E-01 | TP53 (includes EG:22059),BAD,ITPR2,MAPK3,PIK3R1,CCND1,AKT1,MAP2K2,RARB,FOXO3,ITPR3,PIK3CD,PIK3R2,EGFR |
| Butanoate Metabolism | 2.12E00 | 1.23E-01 | ACAT2,RDH11,SUCLG2,SDHC,ELOVL6,AADAC,ALDH1A1,ACAT1,SDHD,DBT,EHHADH,PDHB,HMGCS1,ALDH5A1,HADH,ALDH7A1 |
| Aldosterone Signaling in Epithelial Cells | 2.11E00 | 1.53E-01 | CRYAB,HSPA1A/HSPA1B,PIK3R1,MAPK3,DNAJC3,HSPA4,HSP90B1,PLCE1,HSP90AB1,MAP2K2,DNAJC8,PIK3R2,DNAJC22,HSPB6,DNAJC9,ITPR2,HSPA9,DNAJB9,DNAJB14,PRKCI,DUSP1,PIP5K1C,ITPR3,PIK3CD,DNAJB5,HSPB1 |
| Melanoma Signaling | 2.1E00 | 2.17E-01 | TP53 (includes EG:22059),AKT1,BAD,MAP2K2,MAPK3,PIK3R1,MDM2,PIK3CD,PIK3R2,CCND1 |
| FLT3 Signaling in Hematopoietic Progenitor Cells | 2.09E00 | 1.89E-01 | STAT6,BAD,MAPK3,PIK3R1,EIF4E,SHC1 (includes EG:20416),AKT1,MAP2K2,PTPN11,PIK3CD,STAT2,PIK3R2,ELK1,STAT5B |
| T Cell Receptor Signaling | 2.07E00 | 1.65E-01 | CALM1 (includes others),MAPK3,CD4,PIK3R1,NFATC1,NFKB2,PPP3CC,SHB,IKBKB,IKBKG,NFKBIA,NFAT5,JUN,MAP2K2,PPP3R1,PIK3CD,PIK3R2,ELK1 |
| April Mediated Signaling | 2.06E00 | 2.09E-01 | IKBKB,IKBKG,NFAT5,JUN,NFKBIA,TNFSF13,NFATC1,NFKB2,ELK1 |
| 14-3-3-mediated Signaling | 2.05E00 | 1.67E-01 | YWHAE,BAD,TNFRSF1A,YWHAB,TUBB2C,MAPK3,PIK3R1,GSK3A,BAX,MAP3K5,TUBB,TUBB2B,PLCE1,JUN,AKT1,PRKCI,MAP2K2,PIK3CD,PIK3R2,ELK1 |
| Renin-Angiotensin Signaling | 2.05E00 | 1.59E-01 | ADCY9,PTPN6,PAK4,PAK2,ITPR2,MAPK3,PIK3R1,ADCY3,NFKB2,SHC1 (includes EG:20416),PRKCI,JUN,MAP2K2,CCL2,ITPR3,PRKAG2,PIK3CD,PIK3R2,ELK1,ACE |
| LPS-stimulated MAPK Signaling | 2.03E00 | 1.71E-01 | MAPK3,PIK3R1,MAP3K5,NFKB2,IKBKB,IKBKG,PRKCI,JUN,NFKBIA,MAP2K2,PIK3CD,LBP,PIK3R2,ELK1 |
| Thyroid Cancer Signaling | 2.03E00 | 2.17E-01 | TP53 (includes EG:22059),SHC1 (includes EG:20416),TCF4,MAP2K2,MAPK3,NGF,TCF3,CTNNB1,CCND1,TCF7L2 |
| DNA Methylation and Transcriptional Repression Signaling | 2.02E00 | 2.61E-01 | MECP2,DNMT3A,MTA1,CHD4,SAP18,RBBP4 |
| Myc Mediated Apoptosis Signaling | 2.01E00 | 1.97E-01 | TP53 (includes EG:22059),SHC1 (includes EG:20416),AKT1,BAD,YWHAE,YWHAB,PIK3R1,PIK3CD,PIK3R2,BAX,FASLG,BCL2 |
