# Supplementary material for: Molecular Profiles of HCV Cirrhotic Tissues Derived in a Panel of Markers with Clinical Utility for Hepatocellular Carcinoma Surveillance
Source: PLoS One. 2012 Jul 5;7(7):e40275. doi: 10.1371/journal.pone.0040275 (PMC3390353; doi:10.1371/journal.pone.0040275)
Supplement: Table S2 — Cell cycle deregulated genes HCV-cirrhotic tissue from patients with hepatocellular carcinoma. (DOCX) [file pone.0040275.s006.docx]

**Supplementary Table S2** Cell cycle deregulated genes HCV-cirrhotic tissue from patients with HCC

| Gene |  | Fold change | *p*-value | Cell cycle phase† |  |  |  |  |  | Cell cycle role | Cell cycle trend‡ |
| --- | --- | --- | --- | --- | --- | --- | --- | --- | --- | --- | --- |
| Symbol | Entrez ID |  |  | G1 | G1/S | S | G2 | G2/M | M |  |  |
| CAPNS1 | 826 | -1.50 | 3.8E-07 | X | X |  |  |  |  | Positive regulation | Arrest |
| CCND1 | 595 | 1.49 | 4.4E-04 | X | X | X | X | X | X | Positive regulation | Progression |
| CDK1 | 983 | 1.21 | 3.7E-04 | X |  |  | X | X | X | Positive regulation | Progression |
| CDK2 | 1017 | 1.28 | 8.5E-05 | X | X | X | X | X | X | Positive regulation | Progression |
| CDK11A/CDK11B | 728642 | -1.21 | 4.9E-05 | X |  |  |  |  | X | Delay early G1 – Induce apoptosis | Early progression – arrest at M |
| E2F3 | 1871 | 1.47 | 5.1E-06 | X | X | X |  |  | X | Positive regulation | Progression |
| EIF4E | 1977 | 1.34 | 9.3E-05 | X | X |  |  |  |  | Positive regulation | Progression |
| MDM2 | 4193 | 1.31 | 1.9E-05 | X | X | X | X | X |  | Positive regulation | Progression |
| JUNB | 3726 | -1.44 | 1.0E-05 |  |  | X | X | X |  | Positive regulation | Arrest |
| CDKN2C | 1031 | -1.36 | 1.7E-06 | X | X | X |  |  |  | Negative regulation | Progression |
| FOXO4 | 4303 | -1.34 | 2.4E-05 | X |  |  | X | X |  | Negative regulation | Progression |
| HMGA1***** | 3159 | -1.30 | 1.4E-06 | X | X | X | X |  |  | Negative regulation | Progression |
| KLF4 | 9314 | -1.24 | 3.3E-04 | X | X | X |  |  |  | Negative regulation | Progression |
| NFKBIA | 4792 | -1.29 | 2.7E-04 | X | X |  |  |  |  | Negative regulation | Progression |
| PIN1 | 5300 | -1.47 | 1.9E-05 |  |  |  |  |  | X | Positive regulation | Arrest at M |
| FOXO3***** | 2309 | -1.28 | 1.9E-06 |  |  | X |  |  | X | Negative regulation | Progression |
| NGF | 4803 | -1.38 | 1.9E-06 | X |  |  |  |  | X | Negative regulation | Progression |
| APC***** | 324 | 1.45 | 4.5E-05 | X | X | X |  |  |  | Negative regulation at G1/S | Arrest |
| RBL1 | 5933 | 1.14 | 8.6E-05 | X | X |  |  |  |  | Negative regulation at G1/S | Arrest |
| CUL2 | 8453 | 1.53 | 2.9E-06 | X | X |  |  |  |  | Negative regulation at G1/S | Arrest |
| CUL4A | 8451 | 1.32 | 2.6E-05 | X | X |  | X |  | X | Negative regulation at G1/S | Arrest |
| CUL5 | 8065 | 1.27 | 1.6E-04 | X | X |  |  |  |  | Negative regulation at G1/S | Arrest |
| ESRRG | 2104 | 1.24 | 3.7E-04 | X | X |  |  |  |  | Negative regulation at G1/S | Arrest |
| STRADA | \| 92335 \| 7.29 \| 7.54 \|  \|  \| 1.19 \| \| --- \| --- \| --- \| --- \| --- \| --- \| | 1.19 | 2.3E-05 | X |  |  |  |  |  | Negative regulation at G1 | Arrest |
| PIAS2 | \| 9063 \| 1.26 \| \| --- \| --- \| | 1.26 | 2.2E-05 | X |  |  |  |  |  | Negative regulation at G1 | Arrest |
| GPS2 | \| 2874 \| -1.3 \| \| --- \| --- \| | -1.30 | 1.8E-05 | X |  |  |  |  |  | Negative regulation at G1 | Progression |
| BTG2 | 7832 | -1.39 | 8.1E-07 | X |  |  |  |  |  | Negative regulation at G1 | Progression |
| DUSP1 | 1843 | -1.77 | 9.3E-05 | X |  |  |  |  |  | Negative regulation at G1 | Progression |
| FKBP1A***** | 2280 | -1.62 | 1.8E-07 | X |  |  |  |  |  | Negative regulation at G1 | Progression |
| SMARCB1 | 6598 | -1.25 | 1.5E-05 | X |  |  |  |  |  | Negative regulation at G1 | Progression |
| NFE2L2 | 4780 | 1.41 | 3.1E-07 | X |  |  |  |  |  | Positive regulation at G1 | Progression |
| GSPT1 | 2935 | 1.22 | 1.3E-03 | X | X |  |  |  |  | Positive regulation at G1/S | Progression |
| EZH2 | 2146 | 1.16 | 6.5E-04 |  | X | X |  |  |  | Positive regulation at G1/S | Progression |
| BAD | 572 | 1.24 | 6.1E-04 | X | X | X |  |  |  | Positive regulation at G1/S | Progression |
| RB1CC1 | 9821 | 1.45 | 6.6E-08 | X | X |  |  |  |  | Negative regulation at G1/S | Arrest |
| CAMK2G | 818 | -1.25 | 4.2E-05 | X | X |  |  |  |  | Negative regulation at G1/S | Progression |
| FZR1 | 51343 | -1.22 | 2.2E-05 | X | X |  |  |  |  | Negative regulation at G1/S | Progression |
| ITGB1 | 3688 | -1.10 | 2.2E-04 | X | X | X |  |  |  | Negative regulation at G1/S | Progression |
| LIF | 3976 | -1.42 | 4.4E-04 |  | X |  | X | X |  | Negative regulation at G1/S-G2/M | Progression |
| MYB | 4602 | -1.07 | 6.3E-04 |  | X |  | X | X |  | Negative regulation at G1/S-G2/M | Progression |
| FBXO5 | 26271 | 1.20 | 4.1E-04 | X |  | X |  |  | X | Positive regulation at G1 – S  Stop mitosis | Early Progression |
| PIK3R1 | 5295 | 1.31 | 8.6E-05 | X |  |  | X |  |  | Positive regulation at G1 – G2 | Progression |
| CREBBP | 1387 | -1.18 | 6.0E-05 |  |  | X |  |  |  | Increase S phase intro | Arrest |
| FGF1 | 2246 | -1.13 | 1.2E-03 |  |  | X |  |  |  | Increase S phase intro | Arrest |
| PTOV1 | 53635 | -1.30 | 5.0E-04 |  |  | X |  |  |  | Increase S phase intro | Arrest |
| PEA15***** | 8682 | -1.37 | 3.9E-06 |  |  | X |  |  |  | Increase S phase intro | Arrest |
| CTNND1***** | 1500 | -1.41 | 2.2E-08 |  |  | X |  |  |  | increase S phase duration - reduce proliferation | Progression |
| POLR2A | 5430 | -1.17 | 8.1E-04 |  |  | X |  |  |  | Negative regulation at S phase | Progression |
| AGK | 55750 | 1.35 | 3.8E-04 |  |  | X |  |  |  | Positive regulation at S phase | Progression |
| ZBTB10 | 65986 | 1.53 | 2.4E-04 |  |  | X |  |  |  | Positive S phase progression | Progression |
| REV3L | 5980 | 1.39 | 1.4E-04 |  |  | X |  |  |  | Positive S phase progression | Progression |
| ANP32B | 10541 | 1.25 | 6.5E-04 |  |  | X |  |  |  | Positive S phase progression | Progression |
| APBB1 | 322 | -1.16 | 9.4E-05 |  |  | X |  |  |  | S phase blockage | Progression |
| APBB2 | 323 | 1.14 | 1.9E-04 |  |  | X |  |  |  | S phase blockage | Arrest |
| PTPN11 | 5781 | 1.33 | 6.4E-06 |  |  | X |  | X |  | Positive S phase intro –  Negative regulation at G2/M | Progression-G2/M arrest |
| HMGA1***** | 3159 | -1.19 | 2.3E-04 |  |  | X |  | X |  | Positive S phase intro –  Negative regulation at G2/M | Progression-G2/M arrest |
| CKS2 | 1164 | 1.96 | 3.2E-07 |  |  |  | X |  |  | Positive regulation at G2 phase | Progression |
| ING4 | 51147 | 1.15 | 2.1E-04 |  |  | X |  | X |  | Negative regulation at S – G2/M | Arrest |
| FLNA* | 2316 | -1.67 | 2.2E-10 |  |  |  | X |  |  | Negative regulation at G2 phase | Arrest |
| CSNK2A1 | 1457 | 1.19 | 2.6E-04 |  |  |  | X | X |  | Negative regulation at G2/M | Arrest |
| AURKA | 6790 | 1.37 | 9.2E-05 |  |  | X | X | X |  | Negative regulation at G2/M | Arrest |
| AMACR***** | 23600 | 1.70 | 4.0E-05 |  |  |  | X | X |  | Negative regulation at G2/M | Arrest |
| CAT | 847 | 1.33 | 5.9E-05 |  |  |  | X | X |  | Negative regulation at G2/M | Arrest |
| DPP4***** | 1803 | 1.61 | 1.0E-06 |  |  |  | X | X |  | Negative regulation at G2/M | Arrest |
| MAD2L1 | 4085 | 1.28 | 4.5E-04 |  |  |  | X | X | X | Negative regulation at G2/M | Arrest |
| PCBP4 | 57060 | -1.28 | 4.0E-04 |  |  |  | X | X |  | Negative regulation at G2/M | Progression |
| PPP5C | 5536 | -1.42 | 1.7E-06 |  |  |  | X | X | X | Negative regulation at G2/M | Progression |
| POLD4 | 57804 | -1.32 | 4.0E-05 |  |  |  | X | X |  | Positive regulation at G2/M | Arrest |
| RBX1 | 9978 | 1.22 | 9.2E-04 |  |  |  |  |  |  | Positive regulation at G2/M | Progression |
| RUNX2 | 860 | -1.11 | 4.6E-04 |  |  |  | X | X |  | Positive regulation at G2/M | Arrest |
| MBD4 | 8930 | 1.26 | 8.7E-04 |  |  |  | X | X |  | DNA damage check-point at G2/M | Arrest |
| BRCC3 | 79184 | 1.28 | 1.2E-04 |  |  |  | X | X |  | DNA damage check-point at G2/M | Arrest |
| CDC14B***** | 8555 | 1.64 | 3.0E-07 |  |  |  | X | X |  | DNA damage check-point at G2/M | Arrest |
| HMGN1 | 3150 | 1.20 | 5.7E-04 |  |  |  | X | X |  | DNA damage check-point at G2/M | Arrest |
| RINT1 | 60561 | 1.25 | 1.3E-03 |  |  |  | X | X |  | DNA damage check-point at G2/M | Arrest |
| TOP2A | 7153 | 1.33 | 9.0E-06 |  |  |  |  | X |  | DNA damage check-point at G2/M | Arrest |
| RAD17 | 5884 | 1.36 | 1.8E-05 |  | X |  | X | X |  | DNA damage check-point at G2/M | Arrest |
| NBN | 4683 | 1.27 | 2.6E-05 | X | X | X | X | X | X | DNA damage check-points | Arrest |
| SMC3***** | 9126 | 2.09 | 4.5E-06 |  |  |  | X | X |  | DNA damage check-point at G2/M | Arrest |
| AHSG | 197 | 1.28 | 1.6E-05 |  |  |  |  |  | X | Negative regulation at Mitosis | Arrest |
| TXN | 7295 | 1.28 | 4.2E-05 |  |  |  |  |  | X | Positive regulation at Mitosis | Progression |
| RIC8A | 60626 | -1.20 | 2.2E-05 |  |  |  |  |  | X | Positive regulation at Mitosis | Arrest |
| RAD21 | 5885 | 1.30 | 8.4E-06 |  |  |  |  |  | X | Positive regulation at Mitosis | Progression |
| PROS1 | 5627 | 1.32 | 3.9E-05 |  |  |  |  |  | X | Positive regulation at Mitosis | Progression |
| CRK | 1398 | 1.36 | 7.3E-06 |  |  |  |  |  | X | Positive regulation at Mitosis | Progression |
| C12orf11 | 55726 | 1.36 | 1.4E-05 |  |  |  |  |  | X | Positive regulation at Mitosis | Progression |
| EWSR1 | 2130 | 1.13 | 6.2E-04 |  |  |  |  |  | X | Positive regulation at Mitosis | Progression |
| HMG20B | 10362 | -1.19 | 2.3E-04 |  |  |  |  |  | X | Positive regulation at Mitosis | Arrest |
| TUBB | 203068 | -1.19 | 1.1E-03 |  |  |  |  |  | X | Induce mitotic spindle depolimerization | Progression |
| HAUS3 | 79441 | 1.29 | 1.1E-03 |  |  |  |  |  | X | Positive in mitotic spindle formation and integrity | Progression |
| HAUS5 | 23354 | -1.32 | 3.5E-06 |  |  |  |  |  | X | Positive in mitotic spindle formation and integrity | Arrest |
| HAUS6 | 54801 | 1.26 | 1.1E-04 |  |  |  |  |  | X | Positive in mitotic spindle formation and integrity | Progression |
| KPNA2 | 3838 | 1.43 | 5.0E-04 |  |  |  |  |  | X | Normal migration, division, and integrity of nuclei during mitosis | Progression |
